# Liposomes, Niosomes, Ethosomes, and Transethosomes for Curcumin and Chlorogenic Acid Delivery: Formulation Design and Dermal Performance

**DOI:** 10.3390/antiox15091090

**Published:** 2026-08-30

**Authors:** Andrés C. Arana-Linares, Arley Camilo Patiño, Ana Liliana Giraldo, Constain H. Salamanca, Andres F. Olea

**Affiliations:** 1 Grupo QBAB, Instituto de Ciencias Aplicadas, Facultad de Ingeniería, Universidad Autónoma de Chile, Av. del Valle Sur 534, Santiago 8580640, Chile; andres.arana@cloud.uautonoma.cl; 2 Departamento de Farmacia, Facultad de Ciencias Farmacéuticas y Alimentarias, Universidad de Antioquia, Calle 70 No. 52−21, Medellin 050010, Colombia; arley.patino@udea.edu.co (A.C.P.); liliana.giraldo@udea.edu.co (A.L.G.); 3 Grupo de Investigación Cecoltec, Cecoltec Services SAS, Cra. 43A #18Sur−135, Medellin 050034, Colombia; 4 Grupo de Investigación Ciencia de Materiales Avanzados, Departamento de Química, Facultad de Ciencias, Universidad Nacional de Colombia Sede Medellín, Cra. 65 #59a−110, Medellin 050034, Colombia

**Keywords:** curcumin, chlorogenic acid, vesicular systems, dermal delivery, antioxidant nanocarriers

## Abstract

Polyphenolic antioxidants are incorporated into pharmaceutical, dermopharmaceutical, and cosmetic products because of their capacity to modulate oxidative stress, inflammation, microbial imbalance, skin aging, wound repair, and tumor-related processes. However, formulation is constrained by chemical instability, limited bioavailability, insufficient skin permeation, and degradation during processing or storage. This review integrates the chemical characteristics, natural sources, extraction approaches, antioxidant mechanisms, and evaluation of curcumin and chlorogenic acid, and critically examines their delivery through liposomes, niosomes, ethosomes, and transethosomes. Curcumin is lipophilic and poorly water-soluble, whereas chlorogenic acid is hydrophilic but permeability-limited. Their antioxidant activity is discussed through hydrogen atom transfer, single-electron transfer, interruption of lipid peroxidation, metal chelation, and localization within lipid interfaces, together with chemical, biomimetic, and cellular assessment methods. Vesicular carriers can improve encapsulation, stability, release control, skin interaction, biological performance, and incorporation into semisolid dosage forms. However, these benefits are accompanied by formulation-dependent trade-offs involving manufacturing complexity and cost, long-term stability and reproducibility, excipient-related skin tolerability, scale-up, and an application scope that depends on the intended dermal-delivery endpoint. Therefore, efficacy depends on the interplay among antioxidant properties, vesicle architecture, excipient selection, and processing conditions. Curcumin-loaded vesicles are better documented than chlorogenic-acid-loaded systems, particularly for deformable carriers. Future progress requires quality-by-design strategies, standardized characterization, predictive skin models, long-term stability and safety studies, and scalable manufacturing. Overall, antioxidant-loaded vesicles represent multifunctional platforms for developing stable and effective pharmaceutical and cosmetic products.

## 1. Introduction

Polyphenols constitute a broad family of naturally occurring plant-derived compounds characterized by complex chemical structures containing at least one benzene ring and one or more hydroxyl substituents [1,2]. Up until now, more than 8000 polyphenolic compounds have been identified across different plant species, positioning them as the most abundant class of phytochemicals currently reported [3]. According to their chemical structure, polyphenols are primarily classified into flavonoids (e.g., quercetin [4], catechin [5], and proanthocyanidins [6]), phenolic acids (e.g., ferulic acid [7], caffeic acid [8], and chlorogenic acid [9]), stilbenes (e.g., resveratrol [10]), lignans (e.g., secoisolariciresinol [11] and enterolactone [12]), tannins (e.g., tannic acid [13] and procyanidins [14]), curcuminoids (e.g., curcumin [15]), and other non-flavonoid polyphenols. In addition, polyphenols are widely recognized for their broad spectrum of biological activities, including antioxidant, anti-inflammatory, antimicrobial, cardioprotective, and anticancer properties [16]. Furthermore, these compounds are capable of modulating the activity of enzymes involved in the progression of several pathological conditions, which has stimulated growing interest in their pharmaceutical, nutraceutical, and cosmetic applications, particularly in anti-aging formulations [17].

In particular, the antioxidant activity of polyphenols is primarily associated with their ability to neutralize reactive oxygen species (ROS) and chelate transition metals, thereby contributing to biomolecular protection and the prevention of ultraviolet radiation-induced skin damage [1,18]. Moreover, these compounds have attracted considerable attention in topical formulations due to their ability to modulate enzymes responsible for the degradation of the dermal extracellular matrix, including metalloproteinases, elastase, and hyaluronidase, which are directly involved in processes such as photoaging, loss of skin elasticity, and dehydration [2]. Additionally, the increasing demand for environmentally friendly and naturally derived products has promoted the incorporation of phenolic compounds into advanced dermal formulations [19,20]. This trend has been driven by growing consumer awareness regarding the environmental and health concerns associated with conventional cosmetics, positioning polyphenols as strategic bioactive ingredients in sustainable, high-performance cosmetic and dermopharmaceutical products [21,22].

Among the most extensively studied polyphenols are curcumin and chlorogenic acid, both of which exhibit a broad range of biological activities, although they possess markedly distinct physicochemical properties that critically influence their biopharmaceutical behavior. Curcumin is a highly lipophilic curcuminoid (logP ~ 3.3–3.6) with extremely low aqueous solubility (~0.6 μg/mL), which limits its bioavailability and promotes rapid degradation under environmental conditions such as light exposure and pH variations [23,24]. In contrast, chlorogenic acid is a phenolic acid characterized by higher aqueous solubility (~40 mg/mL) and a predominantly hydrophilic nature; however, its application is constrained by low permeability and high susceptibility to thermal, oxidative, and pH-dependent degradation [25]. These properties are reflected in their classification according to the Biopharmaceutics Classification System (BCS): curcumin is generally assigned to BCS classes II–IV, whereas chlorogenic acid is classified as a BCS class III compound. This distinction underscores their complementary biopharmaceutical limitations, mainly poor aqueous solubility for curcumin and limited membrane permeability for chlorogenic acid [26].

Due to the limitations associated with the poor solubility, physicochemical instability, and reduced permeability of polyphenolic compounds such as curcumin and chlorogenic acid, several conventional topical delivery systems were initially developed, including emulsions [27,28,29,30], gels [31,32,33,34], emulgels [35,36], ointments [37,38], lotions [39,40], and hydrogels [41,42]. Although these formulations can facilitate active-compound incorporation, improve stability, and prolong residence on the skin surface, their performance may still be constrained by the poor solubility of certain antioxidants, susceptibility to chemical degradation, insufficient protection from the external environment, and limited ability to promote partitioning, penetration, and retention across the different skin layers. These limitations have driven the development of more advanced nanostructured and vesicular platforms [43], which can provide more favorable microenvironments for antioxidant incorporation and cutaneous delivery. For example, invasomal vesicles increase curcumin ex vivo permeation by approximately 2.6-fold compared with a conventional gel [44], whereas an ethosomal hydrogel containing resveratrol achieved greater skin permeation and retention than a conventional cream [45]. Similarly, the encapsulation in vesicles increased the permeation of chlorogenic acid by almost twofold, as well as promoting distribution in deeper layers of the skin, compared to hydrogels in which the antioxidant remained in superficial layers of the skin [46].

Vesicular systems represent a particularly relevant category of delivery systems due to their small particle size, high surface area, tunable interfacial properties, and, most importantly, their high biocompatibility [43]. These systems promote enhanced interaction with biological membranes, including the skin, thereby enabling a more efficient transport of bioactive compounds [47]. Vesicular carriers are formed through the self-assembly of amphiphilic molecules, such as phospholipids and nonionic surfactants, into lamellar structures capable of simultaneously encapsulating both hydrophilic compounds, such as chlorogenic acid, and lipophilic compounds, such as curcumin, while also providing protection against chemical degradation [48,49,50,51]. In particular, vesicular systems such as liposomes, niosomes, ethosomes, transferosomes, and transethosomes have demonstrated significant improvements in encapsulation efficiency, physicochemical stability, and transdermal transport across the stratum corneum [47,52,53,54]. Their structural versatility and compositional adaptability enable the modulation of drug release kinetics and enhancement of dermal bioavailability, positioning them as highly effective carriers for polyphenolic compounds [55,56,57,58].

Reviews published to date have addressed the delivery of antioxidants such as curcumin and chlorogenic acid from pharmaceutical and nutritional perspectives or have focused on individual vesicular delivery systems. In this review, we present the current state of knowledge on curcumin and chlorogenic acid, selected as model antioxidants with contrasting physicochemical properties, and on different vesicular platforms (niosomes, liposomes, ethosomes, and transethosomes) as delivery systems. Data reported in the reviewed studies are used to analyze how the physicochemical properties of both antioxidants, their localization within the vesicular structure, vesicle composition, and membrane deformability determine encapsulation, physical and chemical stability, release profiles, skin penetration and retention, and the biological performance of the encapsulated polyphenols. Accordingly, this review discusses the proposed structure–composition–performance relationships across the four vesicular systems with particular emphasis on the topical delivery of curcumin and chlorogenic acid. Importantly, there is a marked difference in the available literature relating these two antioxidants when incorporated into vesicular systems. Curcumin-loaded vesicular formulations have been relatively well investigated, whereas substantially less information is available on vesicular formulations containing chlorogenic acid, particularly those based on ethosomes and transethosomes.

In this context, a literature search was conducted using the Web of Science, PubMed, ScienceDirect, and Taylor & Francis Online databases, employing combinations of the terms “curcumin”, “chlorogenic acid”, “liposome”, “niosome”, “ethosome”, “transfer-some”, “transethosome”, “topical”, “dermal”, “transdermal”, “skin”, “permeation”, and “skin retention”. Priority was given to original research articles, published between 2019 and 2026, addressing the formulation, characterization, and dermal performance of antioxidant-loaded vesicular systems. Analyzed studies were chosen for their relevance on the evaluation of physicochemical properties, released profiles, skin permeation and retention, biological activity, and where applicable, the translational potential of curcumin- and chlorogenic acid-loaded vesicular systems. Earlier studies were also included when necessary to support fundamental concepts related to antioxidant chemistry, physicochemical properties, vesicular self-assembly, characterization techniques, and established mechanisms of cutaneous delivery. Accordingly, the present review critically examines advances in the design and application of these systems for topical delivery, with particular emphasis on the relationships among formulation composition, physicochemical characteristics, release behavior, and in vitro dermal performance, aiming to identify current trends, persistent limitations, and opportunities for the development of more effective delivery strategies.

## 2. Antioxidants: Curcumin and Chlorogenic Acid

An antioxidant is defined as a compound capable of inhibiting or delaying the oxidation of other molecules [59]. The antioxidant activity of curcumin and chlorogenic acid have been extensively studied in food [60,61,62,63], pharmaceutical [64,65,66,67,68], nutraceutical [69,70,71], and cosmetic [72,73,74,75] applications. Their biological activity is primarily associated with the scavenging of reactive oxygen species (ROS) [76,77], transition metal chelation [78,79], inhibition of lipid peroxidation [80,81], and modulation of oxidative stress-related signaling pathways, including Nrf2/ARE and NF-κB [82,83].

The incorporation of antioxidants such as curcumin and chlorogenic acid into pharmaceutical and cosmetic formulations is governed by a shared physicochemical and technological framework, in which these multicomponent systems require the careful control of critical variables, including stability, compatibility, bioavailability, and quality, in order to ensure efficacy and safety throughout their shelf life [56,57]. In this context, it is essential to understand the intrinsic properties of each compound, as these determine not only their biological activity and performance, but also their physicochemical behavior within the formulation system, thereby influencing the design, stability, and functional efficacy of pharmaceutical and cosmetic dosage forms.

### 2.1. Curcumin

**Figure 1:**
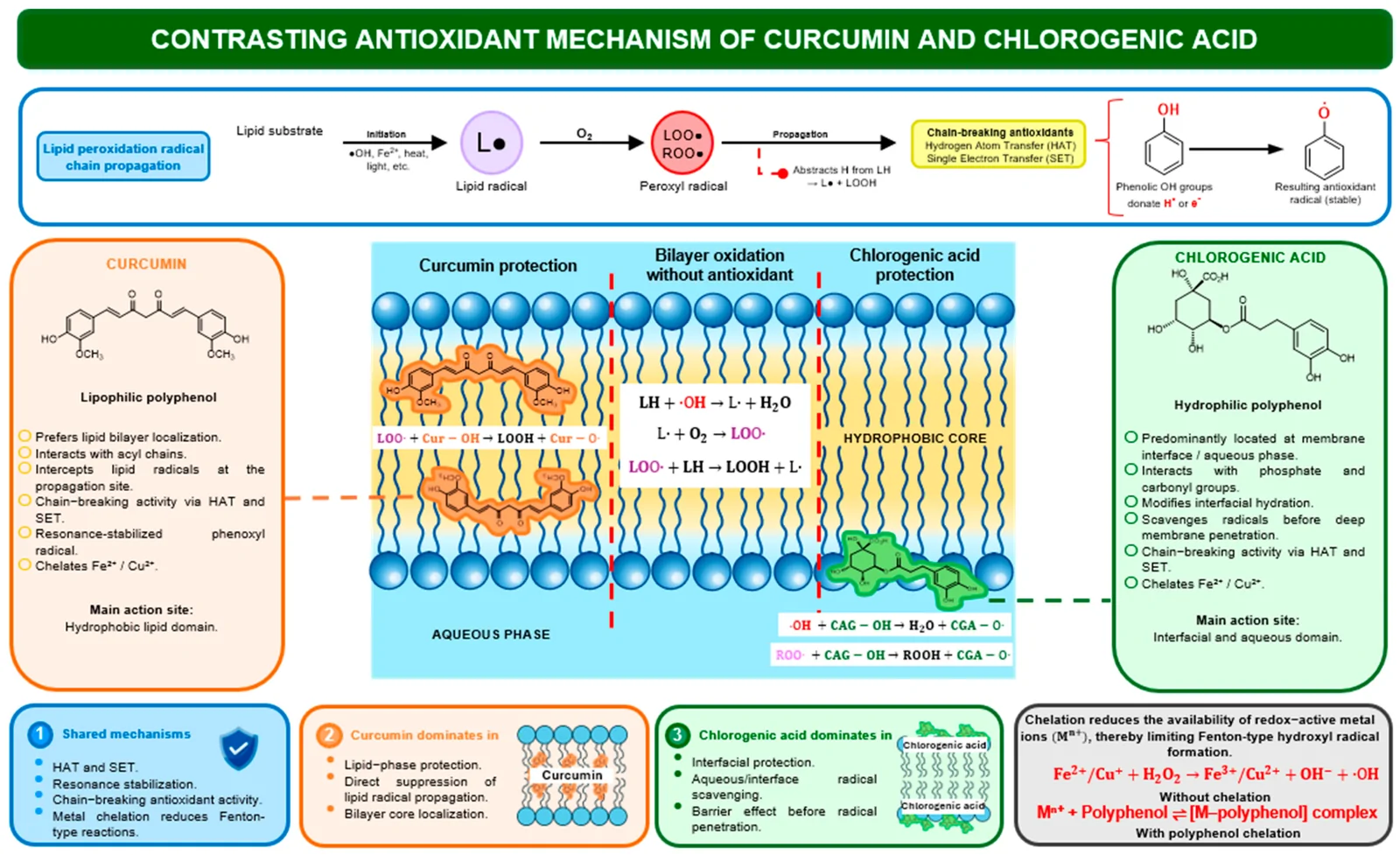
Localization-dependent antioxidant mechanisms of curcumin and chlorogenic acid in phospholipid bilayers. Curcumin (Cur) and chlorogenic acid (CGA) protect phospholipid bilayers through distinct antioxidant mechanisms governed by their physicochemical properties and membrane localization. Curcumin, as a lipophilic polyphenol, preferentially partitions into the hydrophobic acyl-chain region, where it intercepts lipid peroxyl radicals (LOO^●^) and interrupts lipid peroxidation propagation through HAT/SET-mediated chain-breaking activity. In contrast, chlorogenic acid, due to its hydrophilic nature, acts mainly at the membrane interface and aqueous phase, where it interacts with polar head groups, scavenges reactive species before deep membrane penetration, and contributes to metal-chelation-mediated suppression of Fenton-type radical generation.

Curcumin is a lipophilic polyphenolic antioxidant (Figure 1) obtained mainly from *Curcuma longa* rhizomes, whose biological potential is limited by poor aqueous solubility, chemical instability, and low bioavailability. Beyond these constraints, curcumin’s chemical structure and physicochemical characteristics, its natural sources and extraction methodologies, and its wide spectrum of biological and pharmaceutical applications—including antioxidants, anti-inflammatory, antimicrobial, and cytoprotective activities—constitute essential aspects of its scientific relevance. These topics will be expanded in the following subsections, providing a comprehensive overview of curcumin’s properties, limitations, and therapeutic potential.

#### 2.1.1. Chemical Structure and Physicochemical Characteristics of Curcumin

Curcumin is a natural polyphenolic compound belonging to the curcuminoid family and constitutes the principal bioactive metabolite responsible for the characteristic yellow-orange color of turmeric obtained from *Curcuma longa*. Chemically, curcumin is identified as 1,7-bis(4-hydroxy-3-methoxyphenyl)-1,6-heptadiene-3,5-dione, with the molecular formula C_21_H_20_O_6_ and molecular weight of 368.39 g/mol [84]. Structurally, curcumin consists of two aromatic phenolic rings connected through an α,β-unsaturated β-diketone linker, containing hydroxyl and methoxy substituents that contribute to its biological activity and redox behavior [15].

The physicochemical properties of curcumin are strongly governed by its chemical structure. The extended conjugated aromatic system and predominance of hydrophobic moieties confer high lipophilicity, reflected by logP values ranging from 3.3 to 3.6 [26]. In contrast, the limited number of ionizable functional groups significantly restricts its interaction with aqueous environments, resulting in extremely low water solubility (~0.6 μg/mL) [85]. Curcumin contains three labile protons, including one enolic proton associated with the β-diketone moiety and two phenolic protons located on the aromatic rings. Its first apparent dissociation constant has been reported around pKa 8.54, indicating that curcumin remains predominantly non-ionized at neutral and mildly acidic pH values, which further contributes to its poor aqueous solubility and preferential partitioning into hydrophobic domains [86]. Additionally, the β-diketone moiety undergoes keto-enol tautomerism, which influences molecular stability and reactivity depending on environmental conditions such as pH and solvent polarity [87].

Curcumin also exhibits pronounced chemical instability under physiological and environmental conditions. Exposure to light, oxygen, alkaline pH, and elevated temperatures promotes hydrolytic and oxidative degradation, thereby reducing its bioactivity and limiting its incorporation into conventional pharmaceutical and cosmetic formulations [23,24]. From a biopharmaceutical perspective, curcumin is generally classified within Biopharmaceutics Classification System (BCS) classes II–IV, depending on the formulation and experimental conditions, due to its low aqueous solubility and variable permeability [26]. These characteristics substantially compromise its absorption, bioavailability, and therapeutic performance.

#### 2.1.2. Natural Sources and Extraction of Curcumin

Curcumin is primarily obtained from the rhizomes of *Curcuma longa*, a perennial herbaceous species belonging to the Zingiberaceae family and extensively cultivated in tropical and subtropical regions, particularly in India, China, Thailand, and Southeast Asia [88]. The rhizomes of turmeric contain a complex mixture of bioactive compounds, including essential oils, volatile terpenoids, and curcuminoids. Among these, curcumin represents the major curcuminoid, generally accounting for approximately 75–80% of total curcuminoids, followed by demethoxycurcumin and bisdemethoxycurcumin [89]. The total curcuminoid content varies according to geographical origin, cultivation conditions, harvesting time, and postharvest processing [90].

Due to its hydrophobic nature and extremely low aqueous solubility, curcumin is primarily extracted using organic solvents such as ethanol, methanol, acetone, ethyl acetate, and dimethyl sulfoxide. Among these, ethanol has emerged as one of the most suitable extraction solvents because of its high extraction efficiency, low toxicity, and compatibility with pharmaceutical and cosmetic applications [91].

Conventional extraction methods of curcumin include maceration, solvent extraction, hydro-distillation, and Soxhlet extraction, the latter historically regarded as the reference technique for curcuminoid recovery due to its high extraction yield and continuous solvent recirculation process [89]. Nevertheless, these methodologies generally require prolonged extraction times, elevated temperatures, and high energy and solvent consumption, conditions that may promote curcumin degradation through oxidation, thermal decomposition, and photodegradation [91]. To overcome these limitations, several advanced extraction technologies have been developed, including ultrasound-assisted extraction (UAE) [92], microwave-assisted extraction (MAE) [93], enzyme-assisted extraction (EAE) [94], pressurized liquid extraction (PLE) [95], and supercritical fluid extraction (SFE) [96]. These techniques enhance solvent penetration and mass transfer while simultaneously reducing extraction time and solvent usage [91].

#### 2.1.3. Biological and Pharmaceutical Applications of Curcumin 

Curcumin has been extensively investigated for its antioxidant, anti-inflammatory, antimicrobial, immunomodulatory, and cytoprotective activities, which arise from its ability to modulate multiple molecular targets involved in chronic inflammation, oxidative stress, metabolic dysfunction, and tissue damage. It reduces leukocyte recruitment and pro-inflammatory mediators, including COX-2, LOX, iNOS, IL-6, IL-8, and TNF-α, through the regulation of signaling pathways such as NF-κB, MAPK, JNK, and PKC [94]. In chronic inflammatory conditions, curcumin also modulates TLRs, AP-1, JAK/STAT, the NLRP3 inflammasome, and the ROS/Nrf2-Keap1 axis [97,98]. Clinical evidence supports these mechanisms, showing reductions in CRP, TNF-α, IL6, and MDA, together with increased TAC and SOD [99]. Beneficial effects have also been reported in type 2 diabetes, arthritis, renal disorders, and cardiovascular diseases, including improvements in glycemic control, inflammation, pain, mitochondrial function, and lipid metabolism [100,101,102,103]. Its cytoprotective activity is further associated with regulation of the NRF2/Keap1/ARE, AMPK, GPx, SOD, CAT, HO-1, and NQO1 antioxidant systems [104].

The food industry has also taken advantage of the antioxidant and antibacterial properties of curcumin. Curcumin has been reported as a bioactive compound with nutraceutical potential and as a functional component of intelligent food-packaging systems. Xie et al. (2021) described the use of curcumin as a pH-sensitive indicator in the development of pectin/chitosan films for meat packaging [105]. Similarly, Vadivel et al. (2019) incorporated curcumin into biodegradable packaging systems designed to monitor fish freshness [106]. In addition, curcumin has been employed to enhance the antioxidant and antibacterial activities of fermented foods and beverages [107]. Its use as a food colorant [108], a functional additive in food preparation [109], and a preservative for perishable products [110] has also been documented.

Curcumin also has relevant applications in advanced drug delivery systems, biomaterials, and nanotechnological platforms. Nanoparticle-based formulations, lipid nanocarriers, nanofibers, and graphene-based coatings have improved their stability, bioavailability, antimicrobial efficacy, and regenerative capacity compared with the free compound [111,112,113]. These systems have promoted wound healing, collagen synthesis, angiogenesis, antibiofilm activity, neuronal regeneration, and oxidative stress reduction, including effects mediated by M2 microglial polarization, CD74 regulation, and CD44-mediated targeting [112,114]. Overall, these findings consolidate curcumin as a pleiotropic bioactive agent with substantial pharmaceutical, biomedical, and nanotechnological potential.

### 2.2. Chlorogenic Acid

Chlorogenic acid is a hydrophilic polyphenolic antioxidant (Figure 1), chemically identified as a caffeoylquinic acid derivative, and is widely distributed in dietary and medicinal plant sources. Despite its biological relevance, its stability is compromised by hydrolysis, oxidation, pH variations, and processing conditions, which limit its incorporation into conventional formulations. Beyond these constraints, chlorogenic acid’s chemical structure and physicochemical characteristics, its natural sources and extraction strategies, and its broad spectrum of biological and pharmaceutical applications—including antioxidant, anti-inflammatory, metabolic, neuroprotective, nephroprotective, cardioprotective, wound-healing, and antitumor effects—represent central aspects of its scientific importance. These topics will be discussed in detail in the following subsections, providing a comprehensive overview of chlorogenic acid’s properties, limitations, and therapeutic potential.

#### 2.2.1. Chemical Structure and Physicochemical Characteristics of Chlorogenic Acid

Chlorogenic acid is a naturally occurring hydroxycinnamic acid derivative widely found in plants, fruits, vegetables, coffee, and coffee by-products, and is commonly classified within the group of caffeoylquinic acids [115]. Chemically, chlorogenic acid corresponds to an ester formed between caffeic acid and quinic acid, usually described as 5-O-caffeoylquinic acid, although nomenclature may vary depending on the numbering system used for the quinic acid moiety [116]. It has the molecular formula C_16_H_18_O_9_ and a molecular weight of 354.31 g/mol [117]. However, the term chlorogenic acid is often used more broadly to refer to a family of mono- and dicaffeoylquinic acid positional isomers, formed according to the number and esterification site of caffeoyl groups on quinic acid. These include 1-, 3-, 4-, and 5-caffeoylquinic acids, together with 1,3-, 1,4-, 1,5-, 3,4-, 3,5-, and 4,5-dicaffeoylquinic acids. Among them, 3-caffeoylquinic acid and 5-caffeoylquinic acid have received particular attention because of their abundance and biological relevance [115].

The physicochemical behavior of chlorogenic acid differs markedly from that of curcumin. While curcumin is predominantly lipophilic, chlorogenic acid exhibits a predominantly hydrophilic profile, with logP values reported close to neutrality or slightly negative, generally ranging from approximately −0.3 to 0.37 [26,117]. This low lipophilicity is consistent with its high aqueous solubility, which has been reported to be ~40 mg/mL under standard conditions [26]. A critical physicochemical parameter of chlorogenic acid is its dissociation constant, with a pKa value of approximately 3.92, which determines its ionization state, and consequently, its solubility, electrostatic interactions, and affinity for biological or formulation interfaces under different pH conditions [118]. These properties facilitate their incorporation into aqueous media, hydrophilic polymeric matrices [119,120], and the external aqueous phase of dispersed systems, such as emulsion and vesicles [27,46].

From a biopharmaceutical perspective, chlorogenic acid is generally classified as a BCS class III compound, characterized by high solubility but low permeability [26]. This classification is consistent with its predominantly hydrophilic and ionizable nature, which allows chlorogenic acid to be readily dispersed in aqueous and hydrophilic formulation environments. However, this same physicochemical profile limits its affinity for lipid-rich biological domains, particularly the stratum corneum, thereby compromising its passive cutaneous permeation and potentially reducing its bioavailability in deeper skin layers [121].

The chemical stability of chlorogenic acid is also influenced by environmental and formulation-related factors. Chlorogenic acid is particularly prone to hydrolytic degradation due to the presence of an ester bond linking caffeic and quinic acid moieties. Under aqueous conditions, especially in the presence of moisture and varying pH, this bond can be cleaved, leading to the formation of caffeic acid and quinic acid. In addition, chlorogenic acid is susceptible to oxidative degradation in reactive environments, particularly under acidic conditions or in the presence of radical species, resulting in the formation of quinone-type structures with altered chemical reactivity [122,123].

#### 2.2.2. Natural Sources and Extraction of Chlorogenic Acid

Chlorogenic acid is a plant-derived phenolic compound widely distributed in dietary and medicinal plant sources, with green coffee beans representing one of the richest and most extensively studied matrices. In green coffee, chlorogenic acids occur mainly as caffeoylquinic, feruloylquinic, and dicaffeoylquinic acid derivatives, with 5-caffeoylquinic acid generally recognized as the most abundant isomer, accounting for approximately 50% of total chlorogenic acids on a dry-weight basis [115]. Unroasted coffee beans contain particularly high levels of chlorogenic acids; however, their concentration progressively decreases as the degree of roasting increases, indicating that postharvest thermal processing strongly affects their final content [124]. Beyond coffee, relevant amounts of chlorogenic acid have been reported in several fruits and vegetables, including apples [125], prunes [126], tomatoes [127], eggplant [128], carrots [129], potatoes [130], pears [131], berries [132], and artichoke [133]. Among these sources, eggplant is especially notable because chlorogenic acid constitutes the predominant phenolic compound in its pulp and may represent 80–95% of total hydroxycinnamic acids [115].

Due to its hydrophilic nature, chlorogenic acid is mainly extracted with polar solvents such as water, ethanol, methanol, hydroalcoholic mixtures, and aqueous acetone. Conventional techniques, including maceration, infusion, decoction, reflux, and Soxhlet extraction, are simple but often require long times, high solvent volumes, and elevated temperatures, favoring oxidation, hydrolysis, or thermal degradation. Greener alternatives, such as ultrasound, microwave, pressurized-liquid, subcritical-water, and supercritical-fluid extraction, as well as eutectic solvents, ionic liquids, enzymes, and combined methods, improve selectivity and yield [134]. Method selection should balance efficiency, safety, scalability, environmental impact, performance preservation, formulation compatibility, and regulatory acceptability for dermal or pharmaceutical applications [135].

#### 2.2.3. Biological and Pharmaceutical Applications of Chlorogenic Acid

Chlorogenic acid exhibits broad antioxidant, anti-inflammatory, metabolic, neuroprotective, nephroprotective, and cardioprotective activities, largely through the modulation of redox-inflammatory signaling, gut microbiota, epithelial barrier integrity, and apoptotic pathways. In metabolic and renal disease models, it reduced hepatic steatosis, inflammation, serum transaminases, glucose, lipids, serum uric acid, blood urea nitrogen, creatinine, oxidative stress, and fibrosis through pathways including TLR4/NF-κB and PI3K/AKT/mTOR [136,137]. Neuroprotective effects included reductions in infarct volume, neuronal apoptosis, mitochondrial ROS, and NF-κB activation, together with improved cognitive and remyelination-related responses via Sirt1 and Nrf2/HO-1 signaling [138,139]. Chlorogenic acid also attenuated doxorubicin-induced cardiotoxicity by enhancing antioxidant defenses [140] and reduced ROS, COX-2, iNOS, IL-1β, IL-6, and TNF-α in inflammatory models while improving intestinal barrier function and antioxidant status [141,142].

Some studies have presented chlorogenic acid as a possible natural alternative to synthetic preservatives. Chlorogenic acid has antibacterial activity against pathogenic bacteria [143], but probiotic microorganisms used in value-added foods show marked resistance to it [144]. There are also studies describing the use of chlorogenic acid in food preservation; for example, a reduction in anthocyanin degradation in blackberry juice mediated by chlorogenic acid has been reported [145].

In dermal and oncological applications, chlorogenic acid-loaded nanocomposite hydrogels promoted diabetic wound healing through hemostatic, antioxidant, antibacterial, angiogenic, and immunomodulatory effects [146]. In addition, chlorogenic acid showed pro-apoptotic effects in C32 melanoma cells and stronger antiproliferative activity in A375 cells when incorporated into MDM4-targeting PROTACs, inducing p53/p21 activation, G2/M arrest, and apoptosis [147,148]. Finally, chlorogenic acid-loaded chitosan nanoparticles suppressed DMBA-induced skin carcinogenesis by restoring antioxidant markers and detoxification systems [149], whereas in acute pancreatitis, chlorogenic acid reduced MPO, amylase, and inflammatory mediators associated with NF-κB, COX-2, and iNOS [150].

### 2.3. Mechanisms of Antioxidant Action of Curcumin and Chlorogenic Acid

A mechanistic understanding of antioxidant activity in polyphenolic compounds such as curcumin and chlorogenic acid requires considering both their chemical reactivity and their spatial distribution within heterogeneous systems. In biological and biomimetic environments, lipid peroxidation proceeds via a radical chain mechanism, in which peroxyl radicals (LOO^●^, ROO^●^) sustain propagation by abstracting hydrogen atoms from lipid substrates [151]. In this context, polyphenols act primarily as chain-breaking antioxidants, interrupting propagation through hydrogen atom transfer (HAT) and single electron transfer (SET) mechanisms, mediated by phenolic hydroxyl groups and stabilized through resonance delocalization within conjugated π-systems [59].

Both curcumin and chlorogenic acid exhibit these mechanisms (Figure 1); however, their antioxidant efficiency is strongly modulated by their physicochemical properties and microenvironmental distribution. Curcumin, due to its lipophilic character, preferentially localizes within lipid bilayers, where it can directly interact with acyl chains and intercept lipid radicals at the site of propagation [152]. In contrast, chlorogenic acid, being more hydrophilic, is predominantly located at the membrane interface or in the aqueous phase, where it interacts with polar head groups and scavenges radicals before they penetrate deeper into the lipid core [153]. Experimental evidence indicates that chlorogenic acid interacts with the carbonyl and phosphate groups of phospholipids, modifying interfacial hydration and influencing its accessibility to reactive species [154].

Additionally, both compounds contribute to antioxidant protection through metal chelation mechanisms. The presence of catechol or phenolic structures enables the binding of transition metals such as Fe^2+^ and Cu^2+^, thereby reducing the formation of highly reactive hydroxyl radicals via Fenton-type reactions [155]. This complementary mechanism further enhances their protective role in oxidative environments.

Importantly, antioxidant activity cannot be inferred solely from chemical structure or theoretical redox potential. Instead, it depends on a combination of kinetic factors, molecular localization, phase distribution, and system composition [156]. This is particularly relevant in structured systems such as vesicular carriers, where compartmentalization governs the accessibility of antioxidants to oxidizable substrates.

In this context, as a consequence of changes in the physical and chemical stability, release behavior, and bioavailability of curcumin and chlorogenic acid, modifications in the antioxidant activity of the free compounds compared with antioxidants loaded into vesicular systems have been reported. For example, a 129.38% increase in the relative bioavailability of chlorogenic acid was reported when it was delivered in liposomes. In addition, an improved response of antioxidant markers was described by an increase in serum GSH-Px levels from 849.0 ± 54.7 U/mL to 1130.6 ± 298.5 U/mL and hepatic T-AOC levels from 4.1 ± 2.3 U/mL to 4.9 ± 0.5 U/mL, while the MDA levels decreased from 12.7 ± 4.2 nmol/mL to 9.8 ± 1.9 nmol/mL in serum and from 41.5 ± 9.2 nmol/mL to 36.0 ± 4.1 nmol/mL in the liver [157]. On the other hand, in niosomes and proniosomes, the impact on the antioxidant effect depended on the mechanism evaluated. DPPH radical scavenging increased from 83.79 ± 0.62% for free chlorogenic acid to 94.28 ± 0.23% and 92.79 ± 0.55% for niosomes and proniosomes, respectively, whereas Fe^2+^ chelation was slightly lower for the vesicular formulations (78.53 ± 0.43% and 79.58 ± 0.55%) than for free CGA (82.72 ± 0.62%) [56]. Therefore, in this particular case, vesicular encapsulation did not uniformly affect all antioxidant mechanisms.

A similar dependence on carrier architecture was observed for curcumin. For example, in niosomes formulated with eutectic solvents and loaded with curcumin, an increase in the photochemical half-life of the antioxidant from 56.51 days to 158.56 days was observed, together with a reduction in the DPPH IC_50_ from 22.09 to 8.94, corresponding to an ~60% decrease in the concentration required to scavenge 50% of the radicals [158]. Likewise, nanoliposomes derived from glycerol-based proliposomes increased curcumin bioaccessibility from 5.62% to 52.9% compared with free curcumin, and consequently, provided the greatest protection against H_2_O_2_-induced oxidative stress in Lo-2 cells. In particular, the mitochondrial red/green fluorescence ratio was 4.26-fold higher than that observed with free curcumin, while reduced glutathione (GSH) levels were restored to values comparable to those of the untreated cells, and superoxide dismutase (SOD) and catalase (CAT) activities were further increased [159].

Collectively, these findings indicate that the antioxidant response of vesicle-associated polyphenols reflects the interplay among molecular localization, protection against degradation, release and bioavailability, and biological distribution, and therefore cannot be inferred from the intrinsic redox activity of the free antioxidant.

### 2.4. Antioxidant Activity Evaluation of Curcumin and Chlorogenic Acid

The evaluation of antioxidant activity in polyphenolic compounds such as curcumin and chlorogenic acid requires complementary analytical approaches capable of capturing different reaction mechanisms and environmental conditions. Due to the diversity of antioxidant pathways and system complexity, these compounds are commonly assessed using a combination of chemical, biomimetic, and biological models, each providing distinct and context-dependent information [156].

#### 2.4.1. Chemical and Biomimetic Methods

In vitro chemical assays based mainly on SET mechanisms are widely used as initial screening tools. Among them, the DPPH assay evaluates the reduction of the stable nitrogen-centered radical 2,2-diphenyl-1-picrylhydrazyl (DPPH^●^), which exhibits an intense purple color with maximum absorbance at 517 nm. Upon reaction with an antioxidant capable of donating an electron or hydrogen atom, DPPH^●^ is converted into its non-radical form, DPPH-H, producing a decrease in absorbance and a color change from purple to yellow. This response is directly proportional to the radical scavenging capacity of the tested compound, allowing a simple, rapid, and cost-effective estimation of its reducing potential [160].

The ABTS/TEAC assay is another widely used method based on the scavenging of the ABTS^●^^+^ radical cation, generated by chemical or enzymatic oxidation. In its most common form, ABTS^●^^+^ is produced by the oxidation of ABTS with potassium persulfate, yielding a stable blue-green chromophore with maximum absorbance at 734 nm. The addition of antioxidants reduces ABTS^●^^+^ to its non-radical form, leading to a decrease in absorbance proportional to antioxidant capacity. This method is typically expressed as Trolox equivalent antioxidant capacity (TEAC), using Trolox as a reference standard. Its main advantage is its applicability in both aqueous and organic media, which allows the evaluation of hydrophilic and lipophilic antioxidants, as well as complex systems such as biological fluids and nanoformulations [161].

Similarly, the ferric reducing antioxidant power (FRAP) assay measures the ability of antioxidants to reduce Fe^3+^ to Fe^2+^ under acidic conditions. Specifically, the method is based on the reduction of the Fe^3+^–TPTZ complex to Fe^2+^–TPTZ, generating an intense blue-violet chromophore with absorbance at 593 nm. The increase in absorbance is directly proportional to the reducing power of the sample, providing a rapid and reproducible estimation of antioxidant capacity. Although initially developed for plasma antioxidant analysis, FRAP has been widely applied to natural antioxidants, including polyphenols [162].

In the context of vesicular delivery systems, kinetic evaluation of DPPH radical scavenging over time is particularly informative, as it allows antioxidant activity to be correlated with sustained release behavior and the preservation of structural integrity in encapsulated systems. Improvements in solubility, dispersion, and molecular availability induced by nanoencapsulation are often reflected in lower IC_50_ values and increased radical scavenging capacity, especially in vesicular systems such as niosomes [158]. However, these assays are generally performed in homogeneous media and do not fully reproduce the complexity of lipid-rich or biological environments, which may underestimate the contribution of lipophilic antioxidants such as curcumin.

To address these limitations, biomimetic lipid oxidation models have been used to better approximate biological membrane environments. In large unilamellar vesicles (LUVs), lipid peroxidation can be induced using radical initiators such as AAPH and monitored by the spectrophotometric detection of conjugated dienes at 234 nm and secondary oxidation products at 270 nm. These systems enable the kinetic analysis of antioxidant performance through parameters such as lag time (tlag) and propagation rate (k). In this type of model, chlorogenic acid has been shown to prolong tlag and reduce oxidation rates, supporting its role as a chain-breaking antioxidant. Importantly, its activity is influenced by membrane composition, particularly cholesterol content, highlighting the relevance of membrane interactions in antioxidant efficacy [154].

#### 2.4.2. Cellular and Biological Methods

Beyond chemical and biomimetic assays, cellular and biological models provide a more physiologically relevant assessment of antioxidant activity. These approaches allow the evaluation not only of direct radical scavenging, but also of the ability of antioxidants to modulate intracellular redox homeostasis, mitochondrial function, endogenous antioxidant defenses, and cytoprotective signaling pathways under oxidative stress conditions [156].

In cellular models, intracellular ROS are commonly quantified using fluorescent probes such as DCFH-DA, whereas mitochondrial membrane potential is frequently assessed using JC-1. In parallel, biomarkers of endogenous antioxidant defense, including reduced glutathione (GSH) levels and enzymatic activities of superoxide dismutase (SOD) and catalase (CAT), are used to determine the cytoprotective effect of antioxidant compounds. These methods are particularly useful because they distinguish between direct chemical radical scavenging and the modulation of cellular antioxidant systems. For instance, curcumin-loaded nanoliposomes have demonstrated enhanced capacity to regulate ROS levels and antioxidant enzyme activity, contributing to improved cellular resistance to oxidative stress [159].

Chlorogenic acid has also shown multifactorial antioxidant activity in cellular models. In neuronal systems exposed to oxidative stress, chlorogenic acid reduces intracellular ROS levels, restores antioxidant enzyme expression, and modulates mitochondrial function and autophagy-related pathways. These effects indicate that its biological antioxidant role extends beyond direct radical neutralization and involves the regulation of intracellular defense mechanisms and stress-response pathways [163].

From a formulation perspective, encapsulation within vesicular systems significantly influences antioxidant performance in biological environments. Liposomal formulations of chlorogenic acid have shown improved pharmacokinetic behavior, including increased bioavailability and enhanced in vivo antioxidant effects. These responses have been associated with elevated levels of endogenous antioxidant enzymes, such as SOD and GSH-Px, together with reduced lipid peroxidation markers, including malondialdehyde [163]. Likewise, niosomal systems have demonstrated enhanced radical scavenging and metal chelation capacity compared with the free compound, suggesting a potential synergistic effect between the polyphenol and the vesicular matrix [164].

Overall, antioxidant activity should be interpreted according to the experimental model used. Chemical assays provide rapid, reproducible, and useful screening tools; however, they should be complemented with biomimetic, cellular, and in vivo approaches to obtain a more accurate and physiologically relevant assessment. This integrated strategy is particularly important in the development of vesicular delivery systems, where antioxidant efficacy depends not only on the intrinsic reactivity of the polyphenol, but also on its localization, release profile, interaction with the carrier architecture, and biological availability.

## 3. Vesicular Nanocarriers for Dermal Delivery

### Vesicular Systems: Generalities and Self-Assembly

Vesicular systems are self-assembled colloidal platforms composed of one or multiple bilayers formed by amphiphilic lipids or surfactants, enclosing internal aqueous compartments. Their formation is governed by thermodynamic principles driven by the hydrophobic effect and the minimization of Gibbs free energy [48]. In aqueous environments, the structuring of water molecules around hydrophobic chains creates an entropically unfavorable state, promoting aggregation into bilayer structures that reduce interfacial energy and stabilize the system [49].

The structural integrity of these systems results from a balance between van der Waals interactions among hydrophobic chains, repulsive forces between polar headgroups, and entropic contributions of the surrounding medium. These interactions lead to the formation of closed bilayer vesicles with tunable properties depending on composition and processing conditions [48].

From a structural perspective, vesicles are classified according to their lamellarity (unilamellar, oligolamellar, or multilamellar) and size (small, medium, and giant). These parameters determine the internal aqueous volume and the available interfacial area, thereby directly influencing encapsulation efficiency, release kinetics, and physicochemical stability [165,166,167]. In dermal and transdermal applications, nanometric size and controlled polydispersity are critical factors governing interaction with the stratum corneum and penetration pathways, including intercellular, transcellular, and appendageal routes [47].

Cutaneous delivery of antioxidants such as curcumin and chlorogenic acid is limited by two major factors: (i) the highly organized lipid matrix of the stratum corneum—composed primarily of ceramides, cholesterol, and free fatty acids—which restricts molecular transport [168,169]; and (ii) the intrinsic instability of these compounds. In this context, vesicular systems serve not only as delivery carriers, but also as protective microenvironments that modulate redox activity and reduce direct exposure to oxygen and UV radiation [170,171].

**Figure 2:**
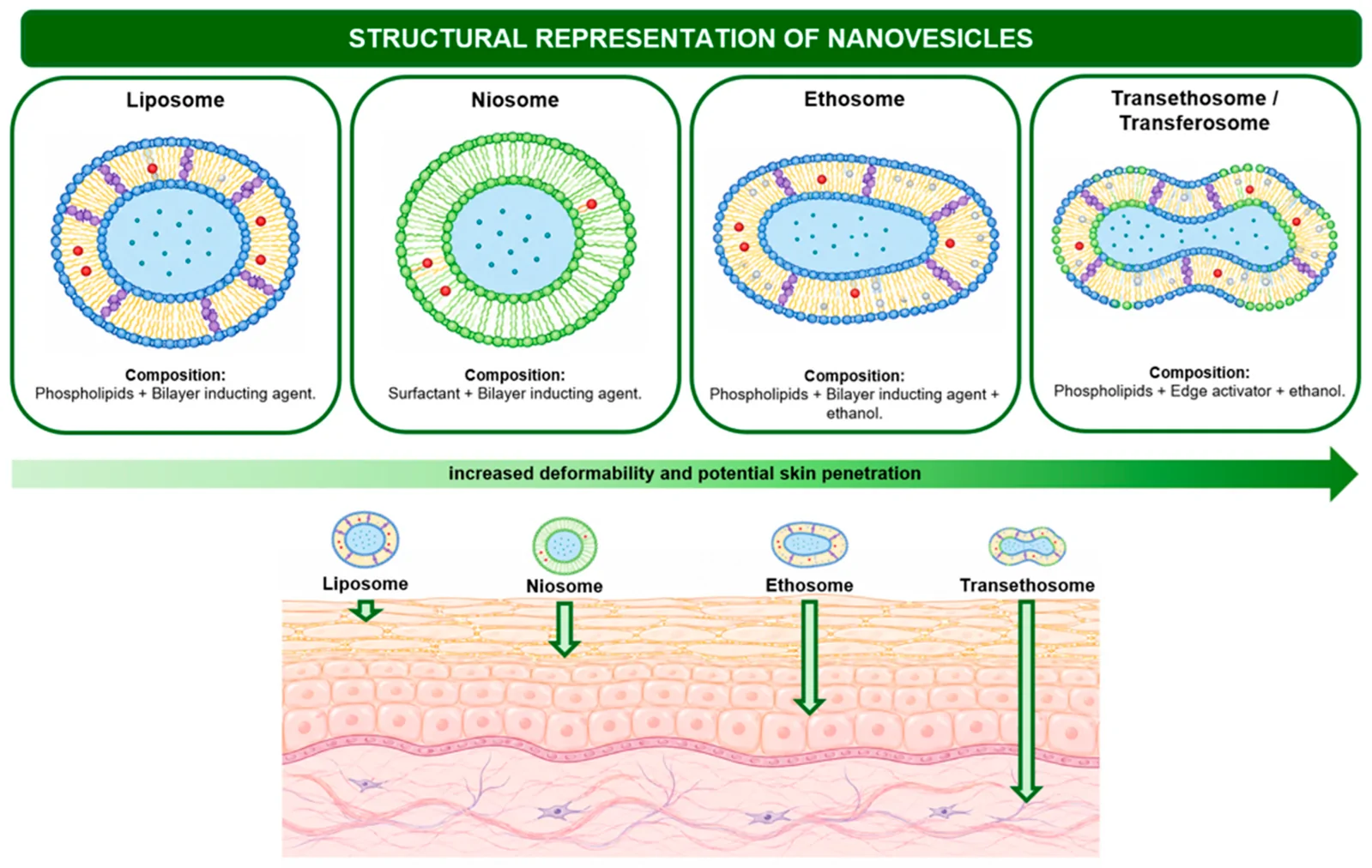
Structural features, deformability gradient, and skin penetration potential of nanovesicular systems. Comparison of structural organization and composition of four nanovesicular systems—liposomes, niosomes, ethosomes, and transethosomes/transferosomes—and illustrates their relative deformability and expected skin penetration behavior. Liposomes and niosomes are represented as more conventional vesicular carriers, whereas ethosomes and transethosomes/transferosomes exhibit enhanced membrane flexibility due to the presence of ethanol and/or edge activators. The lower panel schematically summarizes the general trend of increasing deformability and cutaneous penetration potential from liposomes to transethosomes/transferosomes.

Given the diversity of vesicular platforms and their distinct physicochemical and biopharmaceutical behaviors, a comparative framework is required to rationalize their selection. Table 1 and Figure 2 presents a systematic comparison of niosomes, liposomes, ethosomes, and transethosomes/transferosomes, highlighting a progressive evolution in the design of vesicular systems aimed at optimizing the transdermal delivery of bioactive compounds.

From a structural and compositional perspective, all vesicular systems analyzed derive from a common principle based on the self-assembly of amphiphilic molecules into bilayers enclosing an aqueous core; however, the type of amphiphile and the additional components introduce critical differences in their organization and performance [172,173,174,175,176]. In this context, a progressive transition is observed from simpler systems, such as niosomes based on non-ionic surfactants [172], toward more complex and functionalized systems, such as ethosomes, and particularly, transethosomes, which incorporate phospholipids, high ethanol concentrations, and edge activators that induce lipid disorder, increase bilayer fluidity, and confer high deformability [173]. While liposomes, composed of natural or synthetic phospholipids, exhibit high biocompatibility but are susceptible to oxidation and hydrolysis processes [175], niosomes provide greater chemical stability and economic feasibility by replacing these lipids with non-ionic surfactants, albeit with potential structural variability [177]. In contrast, the incorporation of ethanol in ethosomes and transethosomes enhances interaction with stratum corneum lipids, promoting dermal permeation [173,177], and in the case of transethosomes and transferosomes, the addition of edge activators introduces a higher level of elasticity that optimizes their ability to traverse the skin barrier [178]. This compositional progression thus reflects an evolution from “classical” systems toward platforms specifically engineered to overcome cutaneous permeation limitations; however, this increase in structural complexity is also associated with significant challenges in terms of physicochemical stability, reproducibility, and scalability [177,178,179].

In terms of size, lamellarity, and preparation methods, vesicular systems exhibit partially convergent patterns, as all can form uni- and multilamellar vesicles within comparable nanometric ranges (≈10–1000 nm) using widely established fabrication techniques such as thin-film hydration, ethanol injection, or reverse-phase evaporation [1]. Nevertheless, relevant functional differences arise from both structural organization and methodological complexity. Liposomes display the greatest structural diversity (SUV, LUV, MLV, MVV), enabling the modulation of bioactive compound encapsulation depending on hydrophilic or lipophilic nature [175,177], and they also present the broadest repertoire of preparation methods, including advanced approaches such as supercritical fluid processing or pH-jumping, reflecting their high degree of technological development [175]. Niosomes largely share these methodologies, albeit with lower structural complexity [172]. In contrast, more advanced systems such as ethosomes, and especially transethosomes/transferosomes, tend toward predominantly unilamellar configurations with more controlled sizes (≈100–400 nm) and high deformability, which is critical for transdermal performance [180,181]. This structural feature enhances their ability to dynamically adapt to intercellular spaces within the stratum corneum, facilitating intercellular transport and overcoming the classical limitation of conventional vesicles, whose greater rigidity restricts penetration to superficial skin layers [177,178]. Moreover, these systems employ more specific preparation techniques, such as cold/hot methods or high-pressure homogenization, designed to control ethanol and surfactant incorporation [178,182], highlighting that although these platforms share common technological foundations, process optimization is highly dependent on composition and intended functional performance.

Encapsulation efficiency (EE) in vesicular systems is not an intrinsic property of the nanocarrier type but rather the result of a complex interplay among compositional, structural, and process variables. In this regard, EE shows strong dependence on both preparation method and system composition: niosomes can achieve very high values (>90%), even approaching 97–98% in systems loaded with curcumin or chlorogenic acid, which is associated not only with controlled energy input but also with factors such as vesicle size, surfactant nature, cholesterol presence, and polymer incorporation that promotes dual encapsulation within both the aqueous core and the bilayer [56,183]. Liposomes, in turn, depend more heavily on structural variables such as lamellarity and size, as well as active loading strategies; in this context, surface modifications such as folic acid–TPGS functionalization have enabled encapsulation efficiencies close to 84.85% in chlorogenic acid-loaded systems, underscoring the importance of molecular design in EE optimization [51]. In contrast, ethosomes exhibit consistently high efficiencies due to the solubilizing effect of ethanol, which increases bilayer fluidity and facilitates drug incorporation. In transethosomes/transferosomes, EE is strongly modulated by the phospholipid-to-edge activator ratio, where excess surfactant may induce mixed micelle formation and reduce drug retention [184,185]. Altogether, these findings confirm that encapsulation capacity emerges from the synergy among multiple physicochemical and technological variables, and critically, that strategies aimed at enhancing dermal permeation often involve trade-offs with structural stability and EE.

Characterization methods are largely convergent across systems, reflecting field standardization. Techniques such as dynamic light scattering (DLS) for size determination, zeta potential for surface charge, electron microscopy (TEM/SEM) for morphology, and high-performance liquid chromatography (HPLC) for drug quantification are consistently applied [186,187,188,189]. However, liposomes exhibit a more sophisticated analytical repertoire (e.g., NMR, FFF, SEC) [187,190,191,192], indicating a higher level of technological maturity, likely attributable to their extensive research relevance. In ethosomes and transethosomes, greater emphasis is placed on techniques evaluating skin interaction (e.g., Franz diffusion cells, CLSM), which aligns with their functional orientation [188,193,194].

In terms of stability, a clear gradient emerges. Niosomes demonstrate the highest chemical stability due to the use of non-ionic surfactants and the possibility of formulating proniosomes [177]. Liposomes are more susceptible to degradation, although this can be mitigated through cholesterol incorporation or lyophilization. Ethosomes exhibit intermediate stability: ethanol reduces aggregation but may induce drug leakage [175]. Transethosomes, while relatively stable, are critically dependent on composition and remain sensitive to degradation typical of lipid-based systems [178]. This analysis confirms that the incorporation of permeability-enhancing components (ethanol, surfactants) tends to compromise long-term stability.

The most differentiating aspect lies in dermal delivery mechanisms. Liposomes exhibit limited, predominantly superficial penetration associated with their structural rigidity. Niosomes improve permeation through interaction with skin lipids but still rely on additional strategies. In contrast, ethosomes introduce a dual mechanism: ethanol-induced fluidization of the stratum corneum combined with vesicular penetration [177]. Transethosomes further advance this concept by exploiting ultra-deformability to traverse intact skin via intercellular pathways driven by hydration gradients [178].

From a comparative standpoint, these systems can be organized along a continuum: liposomes (high biocompatibility, low penetration) → niosomes (greater stability and cost-effectiveness) → ethosomes (enhanced permeation due to ethanol effect) → transethosomes/transferosomes (maximized penetration through deformability). This progressive increase in transdermal performance is mechanistically explained by the combination of ethanol-induced stratum corneum fluidization with vesicular penetration in ethosomes, while transethosomes and transferosomes achieve deeper intercellular transport through high deformability and hydration-gradient-driven movement. However, such improvements are accompanied by greater limitations, including formulation complexity, potential skin irritation (particularly due to ethanol or surfactants), reduced stability, and scale-up challenges.

Considering the above, factors such as lipid nature, cholesterol or other stabilizing agent content, surfactant characteristics, and ethanol concentration determine properties such as membrane packing, fluidity, permeability, deformability, and bioactive–bilayer affinity. Likewise, these factors modulate key physicochemical properties, such as vesicle size, encapsulation efficiency, release behavior, and physical and chemical stability, which collectively impact the dermal performance of the encapsulated antioxidants. Therefore, formulation variables should not be interpreted as independent factors, but rather as interconnected determinants within a composition–structure–performance relationship.

Overall, future development of vesicular systems for dermal delivery is expected to depend more on rationally tailoring composition, membrane properties, and processing conditions to the intended dermal-delivery endpoint instead of identifying new and universally superior vesicular systems. In this approach, the comparative analysis requires reported data obtained through similar methodological framework, i.e., standardized characterization, predictive biological models, rational formulation design, and scalable manufacturing strategies [174,176,177,184,195].

**Table 1:** General structural and functional comparison of vesicular systems for dermal delivery.

Parameter	Niosomes	Liposomes	Ethosomes	Transethosomes/Transferosomes
Structure	Single or multiple bilayers of nonionic surfactants + bilayer-inducting agent (membrane stabilizer). Optional: charge inducer agents [172].	Spherical vesicles made of phospholipids enclosing an aqueous core + bilayer-inducting agent [174]. Optional: charger inducer agents [175].	Lipid-based vesicles + ethanol (20–45% *v*/*v*). Optional: penetration enhancer (binary ethosomes) [176].	A lipid bilayer with phospholipids, an edge activator, and an ethanol/aqueous core. Optional: surfactants to increase the skin permeation of drug molecules [173].
Composition	Nonionic surfactants: Polyoxyethlene sorbitan monoesters, Tween 20, 60, 61, 80; polyoxyethlene alkyl ethers, brij30, 35, 52, 58, 72, 76, 92, 97; and sorbitan monoesters, Span 20, 40, 60, and 80 [172]. Bilayer inducting agent: cholesterol, fatty acids [172]. Charge inducer agents: negative (dicetyl phosphate and phosphatidic acid), positive (stearyl amine and stearyl pyridinium chloride) [172].	Phospholipids: natural or synthetic (phosphatidylcholine (PC), phosphatidylethanolamine (PE), phosphatidylserine (PS), phosphatidylinositol (PI), phosphatidylglycerol (PG), and phosphatidic acid) [175]. Bilayer-stabilizer: cholesterol [175]. Optional: polymers (PEG), surfactants, targeting ligands [175].	Phospholipids: Soya phosphatidyl choline, Egg phosphatidyl choline, Dipalmitoyl phosphatidyl choline, Distearyl phosphatidyl choline [182]. Penetration enhancer: Propylene glycol or isopropyl alcohol [176].	Phospholipids: soy lecithin and soy hydrolyzed phosphatidylcholine [196]. Edge activator: 1,2-propanediol/glycerin, 1,2-pentanediol/PEG-40 hydrogenated castor oil/ethoxydiglycol/AH-8,1,3-propanediol, surfactant (Span 80 or Tween 80)/stearylamine [197], Tween 80, Span 80, sodium cholate [176], sodium oleate, sodium deoxycholate, diacetyl phosphate, dipotassium glycyrrhizinate [196].
Lamellarity/Size	Small unilamellar vesicles (SUVs), large unilamellar vesicles (LUVs), and multilamellar vesicles (MLVs) [172]. SUVs: 10–100 nm; LUVs: 100–1000 nm; MLVs: >1000 nm [172]. Typical size range: 50–500 nm [198].	Small unilamellar vesicles (SUVs), large unilamellar vesicles (LUVs), multilamellar vesicles (MLVs) [177], and multivesicular vesicles (MVVs) [175]. SUVs: 10–100 nm; LUVs: 100–250 nm; MLVs: 50–5000 nm [177].	Predominantly unilamellar (LUV-type vesicles) or multilamellar (MLVs) [199]. Typical size range: 120–400 nm [200].	Predominantly unilamellar vesicles (ULVs) with high deformability; may also form multilamellar structures [178]. Typical size range: 100–300 nm [201].
Preparation Methods	Thin Film Hydration Method, Reverse Phase Evaporation, Microfluidics Method, Ethanol Injection Method, Ether Injection Method, Bubble Method, Sonication Method, Transmembrane pH Gradient Method, Heating Method [172].	Thin Film Hydration Method, Reverse Phase Evaporation, Microfluidics Method, Ethanol Injection Method, Ether Injection Method, Bubble Method, Sonication Method, Transmembrane pH Gradient Method [177], Detergent Removal, Dehydration–Rehydration, Heating Method, pH Jumping Method, Supercritical Fluid Method [175].	Cold Method, Hot Method, Thin Film Method [176], Ether Injection Method [182]	Thin Film Hydration Method, Ethanol Injection Method, High-Pressure Homogenization, Sonication Method, Cold and Hot Methods, Reverse Phase Evaporation Method [178].
Encapsulation Efficiency	Method-dependent (~47–99%): higher with increased process control and energy input (e.g., sonication, microfluidics, >90%); intermediate for conventional methods (thin film hydration, reverse phase evaporation, ~70–92%, parameter-dependent); lower for mild/passive approaches (bubble, pH-gradient, ~47–75%) [172].	Dependent on size, lamellarity, and loading method, increases with vesicle size for hydrophilic drugs and is enhanced by active (remote) loading techniques (e.g., pH gradient), while passive loading may result in lower retention and faster release [175].	Formulation-dependent (~30–97%): generally high due to ethanol-enhanced bilayer fluidity and drug solubilization; influenced by ethanol concentration, phospholipid composition, and drug properties, with optimized systems commonly achieving >80% entrapment efficiency [176].	Formulation-dependent (~50–90%): influenced by phospholipid/edge activator ratio and vesicle deformability; optimal compositions achieve high EE (~70–90%), while excess surfactant may reduce entrapment due to membrane destabilization or micelle formation [184,185].
Characterization Methods	Size and distribution size: DLS (Dynamic Light Scattering), SLS (Static Light Scattering), AFM (Atomic Force Microscopy), CLSM (Confocal Laser Scanning Microscopy). Surface properties: Zeta potential (ζ, electrophoretic mobility), probe adsorption, hydrophobic interaction chromatography, contact angle, biphasic partitioning. Morphology/structure: SEM (Scanning Electron Microscopy), TEM (Transmission Electron Microscopy), AFM, CLSM, FTIR (Fourier Transform Infrared Spectroscopy). Composition/bilayer organization: FTIR. Encapsulation efficiency/drug loading: Centrifugation, ultracentrifugation, ultrafiltration (separation-based methods), HPLC (High-Performance Liquid Chromatography). In vitro release: Dialysis (normal/reverse), microdialysis, continuous flow, filtration/centrifugation methods. Permeability/skin penetration: In vitro, ex vivo, in vivo models, organ perfusion, cell culture-based models. Mechanistic/interaction studies: Hydrophobicity and interfacial interaction analyses [186,202].	Size and distribution size: DLS, NTA (Nanoparticle Tracking Analysis), SEC (Size Exclusion Chromatography), FFF (Field Flow Fractionation), CE (Capillary Electrophoresis). Surface properties: Zeta potential (ζ), CE-based charge analysis. Morphology/structure: TEM, Cryo-TEM, FF-TEM (Freeze-Fracture TEM), SEM, AFM, fluorescence microscopy. Composition/bilayer organization: NMR (Nuclear Magnetic Resonance), FTIR, Raman spectroscopy, HPLC. Encapsulation efficiency/drug loading: HPLC, UV–Vis spectroscopy, fluorescence spectroscopy, SEC (free vs. encapsulated separation) In vitro release: Dialysis, ultrafiltration, centrifugation, continuous flow systems Permeability/skin penetration: CE-based permeability, fluorescence assays, diffusion methods Mechanistic/interaction studies: Membrane permeability and interfacial behavior studies [187,190,191,192].	Size and distribution size: DLS (size influenced by ethanol and phospholipid concentration). Surface properties: Zeta potential (ζ) Morphology/structure: TEM, SEM, photomicrography. Composition/bilayer organization: ATR-FTIR (Attenuated Total Reflectance FTIR), Raman spectroscopy, DSC (Differential Scanning Calorimetry). Encapsulation efficiency/drug loading: Ultracentrifugation, HPLC, UV–Vis spectroscopy. In vitro release: Franz diffusion cells. Permeability/skin penetration: Franz diffusion cells, CLSM, fluorescence microscopy. Mechanistic/interaction studies: SEM, TEM, ATR-FTIR, Raman (lipid disordering, ethanol-induced fluidization) [188,193].	Size and distribution size: DLS, PCS (Photon Correlation Spectroscopy), PDI. Surface properties: Zeta potential (ζ, laser Doppler electrophoresis). Morphology/structure: TEM, SEM. Composition/bilayer organization: DSC, FTIR. Encapsulation efficiency/drug loading: HPLC, UV–Vis spectroscopy (after separation of free drug). In vitro release: Franz diffusion cells. Permeability/skin penetration: Franz diffusion cells, CLSM, fluorescence probes. Mechanistic/interaction studies: Vesicle–skin interaction, intercellular vs. transcellular transport, hydration gradient-driven penetration. Deformability (critical parameter): Deformability index (pressure-driven extrusion methods) [178,189,194].
Stability	Resistant to hydrolysis and oxidation; proniosomes (dry precursors) further enhance stability, facilitating storage, transport, and on-demand reconstitution into niosomes [177].	Sensitivity to hydrolysis and oxidation (especially formulations with unsaturated phospholipids) [177]. Moderate stability and improved by cholesterol incorporation and lyophilization techniques for extended shelf-life [175].	Moderate stability. Ethanol improves vesicle fluidity and reduces aggregation, but high alcohol content may compromise long-term stability and cause phospholipid leakage; stability strongly dependent on ethanol concentration and storage conditions [175].	Generally stable systems. Ethanol confers resistance to microbial contamination, while stability depends on phospholipid type (higher with saturated lipids and higher transition temperature) and zeta potential. However, long-term stability may be affected by typical lipid-based vesicle degradation (chemical, physical, biological) [178].
Skin Administration	Niosomes enhance permeation via nanosize, bilayer interaction with skin lipids, and can be further optimized using chemical enhancers (surfactants, fatty acids) or physical methods (microneedles, iontophoresis, ultrasound) [172]. Enhance permeation via multiple mechanisms: temporary disruption of stratum corneum lipid organization, increased hydration and reduced trans epidermal water loss, surface adsorption and fusion generating a thermodynamic gradient, and cellular uptake (fusion and endocytosis) enabling direct drug transfer [177].	Limited by relatively rigid bilayer structure, which in most cases restricts efficient drug delivery across the skin surface [177]. Preferential accumulation in skin appendages (hair follicles, sebaceous glands); however, limited penetration depth due to adhesion and fusion with cell membranes, leading to premature drug release in the upper stratum corneum [174].	Dual mechanism: ethanol fluidizes stratum corneum lipids, while flexible vesicles penetrate and fuse with deeper skin lipids, enabling enhanced drug transport [177].	Ultra-deformable vesicles penetrate intact stratum corneum via intercellular pathways driven by hydration gradient; edge activators enhance membrane flexibility, enabling deep skin penetration and transdermal delivery [178].
Comparative Advantage	Lower production cost and higher industrial feasibility due to inexpensive, widely available non-ionic surfactants; simpler preparation methods with reduced need for specialized equipment or controlled conditions, enabling easier scale-up and application in resource-limited settings [177].	High biocompatibility, biodegradability, and low immunogenicity; ability to encapsulate both hydrophilic and hydrophobic drugs; versatile surface modification (e.g., PEGylation, targeting ligands) enabling controlled, targeted, and sustained drug delivery [175].	Ethanol-rich composition increases bilayer flexibility and stratum corneum permeability, enhancing transdermal delivery [177]; highly deformable vesicles enable deeper penetration and higher drug deposition in skin layers [173]. Improved skin biocompatibility compared to transethosomes (absence of surfactants), while higher alcohol content further enhances vesicle fluidity, stability, and reduces aggregation [176].	Enhanced skin penetration, enabling delivery to deeper skin layers and higher deposition of active compounds, making them highly effective topical carriers [173]; particularly effective for delivery of large molecules such as peptides and proteins [176].
Limitations	Vesicle fusion and surfactant degradation; poor in vitro–in vivo correlation, protein corona formation and rapid clearance; batch variability, limited reproducibility, and challenges in large-scale manufacturing; scarce clinical validation and lack of specific regulatory frameworks [177].	Limited control over penetration depth and drug localization within skin layers; incomplete understanding of penetration mechanisms; variability between in vitro and in vivo performance [174]. Short circulation time due to rapid clearance by the mononuclear phagocyte system [175].	High ethanol content (20–45%) may induce skin irritation and disrupt stratum corneum integrity upon prolonged use. Long-term safety and chronic exposure data remain limited, and tolerability may vary depending on formulation and skin type. Additionally, ethanol-related effects and phospholipid sources may raise stability and regulatory considerations [176].	Sensitive to environmental stress; relatively high formulation complexity [179]. Surfactants may cause skin irritation and drug loss during formulation [178].
Future Perspective	Standardization of preparation and characterization protocols; surface functionalization to improve circulation and targeting; development of predictive in vitro–in vivo models; expansion of clinical studies and implementation of scalable, controlled manufacturing technologies [177].	Elucidation of penetration mechanisms; development of strategies to precisely control penetration depth (via physicochemical tuning and combination with physical enhancement methods); improved predictive in vitro–in vivo models [174].	Future research should focus on improving vesicle stability, loading capacity, and targeting efficiency, as well as developing stimulus-responsive and hybrid systems. Integration with advanced technologies (e.g., microneedles, lipid nanoparticles) and the use of computational tools for formulation optimization are promising. Further clinical validation and long-term safety studies are required for translation [176].	Formulation complexity and strong dependence on composition (edge activators, lipid ratio) affecting stability and reproducibility; risk of vesicle disruption or mixed micelle formation at high surfactant levels; challenges in large-scale manufacturing and high production cost; limited clinical translation and market availability [184,195].

Abbreviation: PC: phosphatidylcholine; PE: phosphatidylethanolamine; PS: phosphatidylserine; PI: phosphatidylinositol; PG: phosphatidylglycerol; PEG: polyethylene glycol; PEG-40: polyethylene glycol 40; AH-8: abbreviated component reported as an edge activator/permeation enhancer, although its full chemical name is not specified in the table; *v*/*v*: volume/volume; SUVs: small unilamellar vesicles; LUVs: large unilamellar vesicles; MLVs: multilamellar vesicles; MVVs: multivesicular vesicles; ULVs: unilamellar vesicles; nm: nanometers; EE: encapsulation efficiency; DLS: dynamic light scattering; SLS: static light scattering; AFM: atomic force microscopy; CLSM: confocal laser scanning microscopy; SEM: scanning electron microscopy; TEM: transmission electron microscopy; FTIR: Fourier transform infrared spectroscopy; HPLC: high-performance liquid chromatography; ζ: zeta potential; NTA: nanoparticle tracking analysis; SEC: size-exclusion chromatography; FFF: field-flow fractionation; CE: capillary electrophoresis; Cryo-TEM: cryogenic transmission electron microscopy; FF-TEM: freeze-fracture transmission electron microscopy; NMR: nuclear magnetic resonance; UV–Vis: ultraviolet–visible spectroscopy; ATR-FTIR: attenuated total reflectance-Fourier transform infrared spectroscopy; DSC: differential scanning calorimetry; PCS: photon correlation spectroscopy; PDI: polydispersity index; pH: potential of hydrogen; e.g.,: for example; vs.: versus/compared with.

## 4. Dermal Application of Antioxidant-Loaded Vesicular Systems: Critical Analysis of Formulation Design, Skin Performance, and Evidence Gaps

The studies summarized in Table 2 and Table 3 reveal a heterogeneous but highly informative landscape in which vesicular systems have been explored as multifunctional platforms for the dermal and transdermal delivery of polyphenolic antioxidants. However, the available evidence is clearly asymmetrical. Curcumin has been investigated across a broader range of vesicular architectures, including liposomes, niosomes, ethosomes, transferosomes, transethosomes, and vesicle-in-hydrogel systems [57,203,204,205,206,207,208,209,210,211,212,213,214]. In contrast, chlorogenic acid remains markedly underrepresented, with direct topical evidence essentially restricted to niosomes/proniosomes and transferosomes/protransferosomes for wound healing [56,215]. In addition, one chlorogenic-acid-loaded liposomal system has been evaluated in systemic melanoma immunotherapy, but not as a topical or dermal delivery platform [216]. Therefore, although chlorogenic acid has strong biological relevance as an antioxidant, anti-inflammatory, wound-healing, and dermato-oncological candidate, its development in classical and advanced vesicular carriers remains at an early stage, particularly for ethosomes, transethosomes, and topical liposomal systems.

### 4.1. Composition–Structure–Performance Relationships in Antioxidant-Loaded Vesicles

Lipid composition provides a clear example of this composition–structure–performance balance. In curcumin-loaded ethosomes, Li et al. reported the highest deformation index (0.66 ± 0.07) for the system with a 1:1 phosphatidylcholine:hydrogenated phosphatidylcholine (PC:HPC) ratio, compared with 0.35 ± 0.03 for ethosomes formulated exclusively with PC. In addition, increasing the HPC content to 75% increased the vesicle particle size from 171.70 ± 1.15 nm to 256.70 ± 11.48 nm and decreased the deformation index to 0.20 ± 0.04, relative to the 1:1 PC:HPC system. However, encapsulation efficiency remained high in all formulations (93.76–97.26%), indicating that phospholipid composition mainly affected the structural and mechanical properties of the vesicles. PC peroxidation decreased upon incorporation of HPC, provided that it was included at concentrations above 25%. Additionally, curcumin release was modified: after 24 h, the PC-based system released approximately 43% of the active compound compared with approximately 20% for the PC:HPC system and approximately 13% for the HPC-based system, which is consistent with the increase in membrane rigidity associated with HPC. However, greater release did not translate into greater skin permeation, since the PC:HPC system achieved the highest transdermal flux (19.54 ± 4.14 ng/cm^2^/h), almost twice that of the PC system (10.18 ± 0.83 ng/cm^2^/h), whereas the HPC system showed a flux of 8.30 ± 2.67 ng/cm^2^/h [211]. These results indicate that a 1:1 PC:HPC ratio provided the most favorable balance among oxidative stability, deformability, and skin transport, demonstrating that maximizing membrane fluidity or bioactive release does not necessarily maximize permeation through the skin.

Surfactant type and cholesterol content exerted similar nonlinear effects on vesicular performance. In a study involving chlorogenic acid, nanotransfersomes (NTs) formulated with Tween 80 exhibited a considerably larger particle size than nanoprotransfersomes (NpTs) containing Span 60 (584.5 ± 27 nm vs. 315.6 ± 13 nm), together with a higher PDI (0.517 vs. 0.423); however, their deformability index was 1.26-fold higher (9.50 ± 0.19 vs. 7.53 ± 0.11). This difference was accompanied by greater cumulative release after 48 h for NTs (99.1 ± 0.07%) than for NpTs (92.1 ± 1.82%; *p* < 0.005), and more importantly, by greater ex vivo permeation: 31.59 ± 1.46% for NTs and 29.03 ± 1.81% for NpTs compared with 14.63 ± 1.15% for the free CGA hydrogel. Steady-state flux followed the same trend, reaching 30.51 ± 1.07 µg/cm^2^/h, 24.04 ± 1.01 µg/cm^2^/h, and 22.58 ± 1.09 µg/cm^2^/h, respectively, corresponding to enhancement ratios of 1.35 ± 0.03 and 1.06 ± 0.08 for NTs and NpTs, respectively [215]. In the curcumin-loaded niosomes, increasing the cholesterol:surfactant ratio from 0:10 to 5:5 reduced the particle size from 444.27 ± 4.86 nm to 383.67 ± 5.03 nm and increased the encapsulation efficiency from 87.42 ± 0.41% to 95.19 ± 0.08%; however, it simultaneously increased the PDI from 0.283 ± 0.009 to 0.493 ± 0.002 and decreased the magnitude of the zeta potential from −9.31 ± 0.97 mV to −0.73 ± 0.31 mV. The formulation with the highest cholesterol content also exhibited the fastest release and the best cutaneous delivery performance, reaching 30.67 ± 0.67 µg/cm^2^ of permeated curcumin and 120.43 ± 1.64 µg/cm^2^ retained within the skin compared with 7.35 ± 3.34 µg/cm^2^ and 72.5 ± 9.32 µg/cm^2^, respectively, for the conventional propylene glycol vehicle [210]. Taken together, these findings indicate that individual physicochemical attributes, such as smaller vesicle size or composition-dependent changes in bilayer organization, cannot independently predict skin transport; rather, dermal delivery arises from the interplay among membrane packing, deformability, release behavior, drug retention, and affinity for the skin barrier.

Ethanol introduces an additional formulation-dependent trade-off that distinguishes ethosomes and transethosomes from conventional phospholipid vesicles. In curcumin-loaded ethosomes, Momekova et al. demonstrated that the effect of ethanol was nonlinear. At a constant phospholipid content, increasing the ethanol concentration from 20% *v*/*v* to 30% *v*/*v* and 40% *v*/*v* progressively reduced the vesicle size from 871.1 ± 16.34 nm to 722.2 ± 7.70 nm and 627.7 ± 15.25 nm, respectively, while the zeta potential changed from −42.14 ± 4.67 mV to −53.24 ± 1.15 mV and −59.28 ± 1.32 mV. However, this size reduction was accompanied by an increase in PDI from 0.16 ± 0.06 to 0.22 ± 0.01 and 0.41 ± 0.03, respectively. In addition, the encapsulation efficiency increased from 69.1 ± 2.21% at 20% *v*/*v* ethanol to a maximum of 81.5 ± 1.61% at 30% *v*/*v*, but subsequently decreased to 75.1 ± 1.19% at 40% *v*/*v*. The authors attributed this decrease to phospholipid solubilization, disruption of the vesicular structure, and the consequent leakage of curcumin. Therefore, although increasing the ethanol content from 20 to 40% *v*/*v* reduced the vesicle size by approximately 28% and increased the magnitude of the zeta potential, the highest ethanol concentration simultaneously affected vesicle homogeneity and bioactive retention [57]. These findings demonstrate that increasing the ethanol concentration does not necessarily improve the overall vesicular performance and that an intermediate concentration or concentration range may be required to balance vesicle size reduction, colloidal stability, vesicle homogeneity, and bioactive antioxidant retention.

### 4.2. Curcumin-Loaded Vesicular Systems

For curcumin, the diversity of reported vesicular systems reflects both its formulation challenges and its high compatibility with lipid-based nanocarriers. Across the analyzed studies, high encapsulation efficiencies were frequently reported, often above 80–90%, especially in liposomes, niosomes, ethosomes, transferosomes, and transethosomes [204,207,209,210,211,212,213,214]. This behavior is consistent with curcumin preferentially partitioning into hydrophobic bilayer regions. However, the studies also report that high efficiency of encapsulation alone is insufficient to predict dermal performance. Vesicle size, PDI, surface charge, bilayer rigidity, deformability, ethanol content, edge activator chemistry, and semisolid vehicle composition collectively determine whether the system behaves as a superficial reservoir, a dermal deposition platform, or a true transdermal carrier [205,207,211,214].

Liposome-based systems illustrate this point clearly. Conventional liposomes improve curcumin dispersion, protect the compound from direct degradation, and support sustained release, but their relatively rigid bilayer structure may restrict deeper skin penetration unless additional formulation strategies are introduced. Curcumin-loaded lipogels demonstrated sustained release, antioxidant activity, antibacterial activity, cytocompatibility, and pro-angiogenic potential in wound-healing-oriented platforms [203]. Likewise, liposomal co-delivery of curcumin and ibrutinib in Carbopol gel enhanced ex vivo skin permeation and improved anti-psoriatic outcomes in an imiquimod-induced psoriasis model. However, in these systems, the final biological effect cannot always be attributed exclusively to curcumin or to the vesicular carrier, because polymeric matrices, co-loaded drugs, and disease-specific inflammatory models contribute substantially to the observed outcomes [204]. Thus, liposomal platforms should be interpreted not merely as passive carriers but as multicomponent delivery systems in which the vesicle and the semisolid matrix jointly regulate release, retention, and biological response.

Niosomes represent a second important strategy, especially from a translational and industrial perspective. Curcumin-loaded niosomes showed high encapsulation efficiency and improved dermal retention or biological activity in several studies [208,209,210]. Nevertheless, their performance was strongly formulation-dependent. Some niosomal systems showed relatively high PDI or even micrometric vesicle size, which may compromise reproducibility, skin penetration, and regulatory acceptability [208]. In contrast, hyaluronan-containing niosomes showed the added value of polymer-associated vesicular stabilization, improving colloidal stability, drug retention, and sustained release [209]. This suggests that niosomes may be particularly useful when engineered as hybrid vesicle–polymer systems rather than as simple surfactant vesicles.

Within curcumin-loaded ethosomes, the available evidence confirms that dermal performance depends on the optimized balance between bilayer fluidity and structural stability rather than on maximal membrane fluidization, as discussed quantitatively in Section 4.1.

Functionalized ethosomes further support the idea that vesicular carriers can integrate pharmacological and technological functions. TPGS-assisted ethosomes co-loaded with curcumin and glycyrrhetinic acid improved percutaneous delivery, dermal exposure, anti-inflammatory activity, and antipsoriatic efficacy [212]. In this case, TPGS was not only a stabilizer or permeation enhancer, but also contributed to antioxidant protection and vesicle functionalization. Similarly, curcumin-loaded gelatin ethosomal gels combined ethanol/phospholipid-mediated skin diffusion with the residence and release-modulating properties of a semisolid matrix [213]. These studies reinforce that ethosomal performance is governed by the interaction between vesicular composition, penetration enhancement, local retention, and disease-relevant biological mechanisms.

In curcumin-loaded transferosomes, surface charge and surfactant type played a central role in determining skin penetration, anti-inflammatory activity, and antibacterial performance. Cationic deformable vesicles enhanced interaction with negatively charged biological membranes and bacterial surfaces, whereas anionic systems showed stronger early penetration in some models, probably due to the membrane-interactive effect of sodium deoxycholate [205]. Curcumin-loaded transferosomal gels also improved ex vivo permeation and systemic exposure compared with conventional curcumin formulations, although their therapeutic target was transdermal systemic delivery rather than local cosmetic or dermal therapy [207]. Therefore, transferosomes should be interpreted according to the intended performance endpoint: local retention, dermal penetration, or systemic transdermal input.

Transethosomes appear especially promising for localized curcumin delivery because they combine ethanol-mediated stratum corneum lipid fluidization with surfactant-induced deformability. In human skin, curcumin-loaded transethosomes increased deposition in the stratum corneum, epidermis, and dermis without detectable receptor-phase permeation. This is highly relevant because it indicates that transethosomes may be more suitable for localized dermal therapy and wound healing than for systemic transdermal delivery. In this case, the absence of receptor-phase permeation should not be interpreted as failure, but as evidence of preferential local retention. Moreover, vesicular encapsulation preserved antimicrobial activity while reducing fibroblast cytotoxicity, suggesting an improved therapeutic window for wound-healing applications [214].

The incorporation of vesicles into hydrogels or semisolid matrices emerges as a recurrent and strategically important feature. Hydrogels convert low-viscosity colloidal dispersions into clinically and cosmetically acceptable topical dosage forms, improving residence time, spreadability, mechanical integrity, and patient or consumer acceptability [203,204,207,208,210,213]. More importantly, they introduce a second diffusional barrier that can modulate burst release, prolong local exposure, and reduce uncontrolled leakage. Therefore, vesicle-in-gel systems should be considered hierarchical delivery platforms rather than simple physical mixtures. Their performance depends on the interplay between vesicle composition, polymer network structure, drug–polymer interactions, and skin contact time [203,204,207,213]. This is particularly relevant for wound healing, psoriasis, acne, and inflammatory skin conditions, where prolonged local exposure may be more therapeutically relevant than rapid transdermal flux [203,204,208,212,213,214].

### 4.3. Chlorogenic Acid-Loaded Vesicular Systems

In contrast to curcumin, the vesicular delivery of chlorogenic acid remains poorly explored. CGA-loaded vesicular systems face a dual challenge: retaining the molecule inside or at the interface of the vesicle while simultaneously promoting its passage through the stratum corneum. For CGA, the available evidence similarly indicates that deformability may outweigh particle size as a determinant factor of skin transport, although this conclusion is currently supported by a very limited number of studies [56,215]. Human or porcine skin studies, layer-specific CGA retention, molecular wound-healing markers, long-term stability, and irritation studies are still needed.

The sialic-acid-modified CGA liposomal system adds a different type of relevance. Although it is not a topical or dermal delivery study, it is valuable for dermato-oncology because it demonstrates that liposomal CGA can be engineered as an immunomodulatory nanocarrier capable of reshaping the tumor microenvironment. In that system, sialic acid modification did not substantially alter vesicle size or encapsulation efficiency but changed biological targeting by promoting tumor-associated macrophage interaction and improving the response to anti-PD-1 therapy. This finding should not be used as evidence of skin delivery, but it supports the broader pharmaceutical relevance of CGA-loaded vesicles in melanoma-related contexts. The distinction is important: CGA vesicular systems may be relevant to dermato-oncology, but the current literature does not yet establish their efficacy as topical melanoma or dermal delivery platforms [216].

### 4.4. Cross-Study Inconsistencies, Methodological Limitations, and Translational Challenges

Overall, the comparative analysis indicates that vesicular performance is not carrier-type dependent in a simplistic sense. Liposomes, niosomes, ethosomes, transferosomes, and transethosomes should not be ranked as universally superior or inferior. Instead, their performance depends on how well the carrier architecture matches the physicochemical limitations of the antioxidant and the intended biological target. For curcumin, the main formulation goal is to improve solubilization, protect the molecule from degradation, and control its partitioning into skin lipids [203,204,205,206,207,208,209,210,211,212,213,214]. For chlorogenic acid, the main challenge is to overcome poor lipid-domain affinity while preserving chemical stability and sufficient local retention [56,215,216]. Thus, curcumin benefits from lipid-domain accommodation, whereas CGA requires interfacial engineering, deformability, and possibly polymer-assisted retention.

From a translational perspective, several limitations remain. Many studies lack formal quality-by-design approaches, standardized deformability measurements, lamellarity analysis, long-term stability, chemical stability of the antioxidant, and quantitative skin-layer distribution [57,203,204,205,206,207,208,209,210,211,212,213,214]. The frequent use of different membranes, receptor media, animal skin models, release methods, and biological assays makes cross-study comparison difficult [57,204,207,210,211,212,213,214]. Moreover, ethanol- and surfactant-rich systems, although effective in enhancing skin delivery, require a careful evaluation of irritation, barrier disruption, chronic tolerability, and regulatory acceptability, especially for cosmetic and dermopharmaceutical applications [57,211,212,213,214]. These issues are particularly relevant for transethosomes and highly deformable vesicles, where improved penetration may come at the cost of increased formulation complexity and potential tolerability concerns [57,214].

Across the studies reviewed, several inconsistencies can be identified that caution against interpreting isolated physicochemical parameters as universal predictors of the dermal performance of the vesicular systems discussed. For example, smaller vesicles did not consistently result in greater skin transport, as illustrated by CGA-loaded systems in which larger but more deformable vesicles exhibited superior permeation [56,215]. Likewise, high EE did not necessarily lead to improved topical performance, because retention and release kinetics of the bioactive compound, membrane flexibility, and/or vesicle-stratum corneum interactions also contributed to the overall outcome. Similarly, rapid release of the bioactive compound should not automatically be interpreted as advantageous, since for locally active formulations, sustained release and preferential retention within skin layers, such as the epidermis or dermis, may be more relevant. This is supported by results obtained with a curcumin-loaded transethosomal system on human skin, which showed the absence of detectable drug in the receptor phase, along with greater deposition within the stratum corneum, epidermis, and dermis [214]. These findings emphasize that the intended application, such as superficial retention, dermal deposition, or systemic transdermal delivery, defines the appropriate performance endpoint.

It is worth mentioning that comparative analysis across studies is severely limited due to substantial methodological heterogeneity. Among the studies reviewed, a wide range of biological models was used to evaluate dermal performance, including rodent, porcine, and human skin, together with differences in membrane thickness, receptor media, drug dose, exposure time, sampling protocols, and analytical methods [57,204,207,210,211,212,213,214,215]. Consequently, apparently superior biopharmaceutical outcomes, such as higher flux, permeability, or retention, cannot always be attributed exclusively to the intrinsic performance of a given vesicular architecture. Heterogeneity among experimental models must also be considered; for example, rodent skin provides a barrier environment that differs from that of human skin, whereas dialysis membranes assess drug release rather than true cutaneous permeation. In addition, several studies relied on UV–Vis quantification without specific determination of the bioactive compound within individual skin layers, and relatively few investigated lamellarity, membrane organization, deformability, or the chemical integrity of the encapsulated antioxidant. These methodological differences substantially limit quantitative cross-study comparisons among liposomes, niosomes, ethosomes, transfersomes, and transethosomes.

Stability and translational readiness also remain insufficiently addressed. Many studies reported short-term changes in vesicle size, PDI, zeta potential, or encapsulation efficiency without simultaneously quantifying curcumin or chlorogenic acid degradation, which implies that colloidal stability cannot necessarily be equated with chemical stability of the antioxidant. Moreover, highly deformable systems often rely on ethanol or surfactant concentrations to enhance interaction with the skin but may also increase drug leakage, membrane destabilization, irritation, or chronic disruption of the skin barrier. Few studies have systematically addressed long-term storage, batch-to-batch reproducibility, residual solvents, scale-dependent processing variables, sterilization or preservation requirements, or manufacturing scalability. Therefore, for vesicular dispersions, the transition from proof-of-concept to reproducible pharmaceutical, dermopharmaceutical, or cosmetic products will require the simultaneous consideration of release performance, antioxidant chemical stability, tolerability, manufacturability, and the properties of the final semisolid dosage form.

Finally, the current evidence supports vesicular systems as promising platforms for antioxidant dermal delivery, but the maturity of the field differs substantially between curcumin and chlorogenic acid. Curcumin-loaded vesicles have reached a more advanced stage, with multiple carrier types, semisolid platforms, ex vivo skin models, and disease-oriented biological evaluations. In contrast, CGA-loaded vesicular systems remain underdeveloped, with very limited information available for niosomes, liposomes, transferosomes, ethosomes, and transethosomes, and almost no direct evidence for CGA ethosomal or transethosomal topical delivery. This gap represents a clear opportunity for future research. Rationally designed CGA vesicular systems, especially deformable vesicles integrated into bioadhesive hydrogels, could address the main limitations of this hydrophilic antioxidant and expand its potential in wound healing, inflammatory skin disorders, cosmetic antioxidant protection, and dermato-oncological applications.

## 5. Challenges and Future Perspectives

Despite the significant progress achieved in the development of antioxidant-loaded vesicular systems, several remaining challenges still limit their application in effective pharmaceutical, dermopharmaceutical, and cosmetic products. From a manufacturing perspective, vesicular system production should migrate from small-scale, batch-dependent preparation toward controlled and scalable processes. Conventional methods such as thin-film hydration, useful at the laboratory scale, may generate heterogeneous vesicle populations limiting their industrial scale-up. On the other hand, extrusion, high-pressure homogenization, microfluidic, and continuous-flow technologies provide tighter control of particle formation and improved reproducibility and scalability [217]. In this context, it would be critical to establish relationships between critical material attributes (CMAs), critical process parameters (CPPs), and critical quality attributes (CQAs) [217]. Additionally, real-time monitoring of variables such as temperature, mixing speed, and flow rate during scale-up can be obtained by applying process analytical technology (PAT). At the same time, scalability must be evaluated together with stability, since liposomal systems remain susceptible to aggregation, phospholipid oxidation or hydrolysis, poor redispersibility, and drug leakage during processing and storage [218]. Lyophilization may mitigate some of these limitations, although freezing and dehydration can compromise bilayer integrity and therefore require formulation-specific optimization [217,218]. Consequently, scale-up studies should demonstrate preservation not only of particle size, PDI, and encapsulation efficiency, but also of release behavior and the chemical integrity of the incorporated antioxidant, particularly because long-term stability and manufacturing reproducibility remain major barriers to industrial translation [219]. Finally, manufacturing strategies should also consider green-chemistry principles and process simplification to reduce production costs and environmental impact [220].

Regulatory requirements must be considered in the early development of antioxidant-loaded vesicular systems. Currently, regulatory guidance for liposomal products requires a detailed physicochemical characterization, control of particle-size distribution, chemical composition, stability, manufacturing conditions, and product performance to demonstrate manufacturing consistency, and where applicable, bioequivalence or pharmacokinetic performance [217,220]. Future dermal vesicular formulations should therefore define route-appropriate CQAs that include relevant structural attributes, free and encapsulated bioactive fractions, release behavior, antioxidant chemical stability, and reproducibility throughout shelf life and scale-up. This is particularly important because increasing structural and compositional complexity can hinder manufacturing reproducibility and compliance of regulatory constraints [218,219,220].

Overall, future advances will likely depend on hybrid systems that combine vesicular carriers with bioadhesive polymers, stimuli-responsive components, sustainable excipients, computational optimization, and clinically relevant biological models. These strategies could enable more effective, stable, and industrially applicable antioxidant formulations for the pharmaceutical and cosmetic sectors.

## 6. Conclusions

This review demonstrates that the dermal performance of antioxidant-loaded vesicular systems, including niosomes, liposomes, ethosomes, and transethosomes containing compounds such as curcumin and chlorogenic acid, arises from the interplay between the physicochemical properties of the bioactive compound, its localization within the nanocarrier, vehicle composition, vesicle deformability, and the intrinsic characteristics of the intended delivery endpoint. Owing to its lipophilic nature, curcumin can be readily accommodated within the hydrophobic domains of vesicular structures and has consequently been investigated across a relatively broad range of liposomes, niosomes, ethosomes, transfersomes, and transethosomes. In contrast, chlorogenic acid remains substantially less explored even though it shows poor cutaneous permeation, but at the same time, exhibits low vesicular retention. It is worth mentioning that the analyzed studies indicate that greater vesicle deformability or faster drug release does not necessarily result in superior dermal performance, particularly when local skin deposition rather than systemic transdermal delivery is the desired outcome. Furthermore, methodological heterogeneity, limited evidence from human skin models, insufficient assessment of chemical and long-term stability, and persistent manufacturing and regulatory restrictions make it difficult for a direct comparison of experimental findings and formulations for their transfer to the pharmaceutical or dermo-cosmetic applications. Thus, future development should prioritize the rational design of vesicle composition, structure, and performance, together with standardized characterization approaches, more predictive skin models, and scalable manufacturing strategies, rather than the pursuit of a universally superior vesicular carrier.

## Figures and Tables

**Table 2:** Formulation design and physicochemical characterization of curcumin- and chlorogenic-acid-loaded vesicular systems for dermal applications.

No.	Dermal System	Dermal Evidence Level	Dermal Application/ Biological Context	Composition	Preparation/Optimization Approach	Vesicular Physicochemical Parameters	Key Vesicular Attributes	Ref.
Curcumin (Cur)								
1	Liposome in hydrogel (lipogel)	Skin-related biological evidence Formulation performance	Wound-healing dermal hydrogel with antioxidant, antibacterial, cytocompatibility, and pro-angiogenic assessment.	Liposome: SPC, SL, Chol. Hydrogel: PAM, sodium alginate, CaCl_2_.	Thin-film hydration method. No formal DoE; formulation comparison based on Cur concentration.	Size: 363.3 ± 2.9 nm–678.4 ± 3.5 nm PDI: 0.557–0.719. ZP: NR. EE: 84.6–88.7%	UV–Vis confirmed Cur encapsulation/solubilization at λ_max_: 417 nm. TGA and FTIR supported hydrogel–liposome integration. Moisture uptake remained relatively slow up to 4 days under high relative humidity. Tensile strength increased with Cur-loaded liposome content, attributed to liposome dispersion and hydrogen bonding within the PAM/alginate matrix	[203]
2	Liposome in hydrogel (lipogel)	Direct dermal evidence Skin-related biological evidence Formulation performance	Topical anti-psoriatic therapy in imiquimod-induced psoriasis; co-delivery strategy based on inhibition by ibrutinib and anti-inflammatory activity of Cur.	Liposome: EL, Chol; co-encapsulation with ibrutinib (Ibr). Hydrogel: Carbopol 940, TEA.	Modified solvent injection method. No formal DoE; empirical/sequential optimization by lipid screening, Chol:lipid ratio screening, Ibr:Cur synergistic ratio selection, polymer screening, and short-term stability assessment.	Size: 112.25 ± 1.48 nm–128.53 ± 1.18 nm. PDI: 0.157 ± 0.01–0.174 ± 0.08. ZP: +36.16 ± 0.35 mV. EE: 86.69 ± 3.80% (Cur) and 83.15 ± 1.91% (Ibr).	TEM confirmed the vesicular morphology. Stability was evaluated for 30 days at 4 °C and 25 °C/65% RH. At 4 °C, vesicle size increased by 6.04% and PDI by 26.44%, whereas EE decreased by 8.72% for Ibr and by 10.30% for Cur. At 25 °C/65% RH, vesicle size increased by 4.72%, PDI by 12.07%, and EE decreased by 4.77% for Ibr and by 6.84% for Cur.	[204]
3	Transferosome (deformable liposome)	Direct dermal evidence Skin-related biological evidence Formulation performance	Multitargeted topical therapy for inflamed and infected skin, especially chronic wounds and burns; focus on anti-inflammatory, antibacterial, skin-cell compatibility, and human skin penetration performance.	SPC/P20 for NDLs, SPC/P20/SA for CDL, SPC/SDC for ADLs.	Thin-film hydration method. No formal DoE; comparative formulation design based on surface charge and surfactant type.	Size: 231.5 ± 68.1 nm–299.3 ± 22.8 nm (main population); 130.8 ± 14.5 nm–392.3 ± 61.8 nm (minor population). PDI: 0.17 ± 0.02–0.22 ± 0.04. ZP: −2.3 ± 0.2 mV (NDLs); +33.7 ± 1.1 mV (CDLs); −34.9 ± 0.5 mV (ADLs). EE: NR. EI: 4.72 (NDLs); 6.38 ± 0.90 (CDLs); 5.46 (ADLs).	All formulations had PDI <0.25, supporting acceptable homogeneity. Membrane elasticity confirmed deformable behavior. Cur decreased elasticity in NDLs and ADLs compared with empty vesicles, but increased elasticity in CDLs compared with empty CDLs, probably by modifying lipid/surfactant packing in the presence of SA.	[205]
4	Liposome	Direct dermal evidence Skin-related biological evidence Formulation performance	Transdermal delivery for psoriasis; designed to improve curcumin solubility, skin permeation, keratinocyte uptake, and anti-inflammatory activity. Native Cur was converted into curcumin succinate and then paired with betaine to form Curcumin-Betain-Ionic liquid (Cur-Bet-IL) before liposomal encapsulation.	SL, Chol.	Ethanol volatilization/film-forming method. No formal DoE; empirical ratio screening plus theoretical calculation. No formal DoE; sequential empirical screening of Cur-Bet-IL molar ratio and SL/Chol/Cur-Bet-IL mass ratio, supported by computational analyses of electrostatic potential and non-covalent interactions.	Size: 35.92–60.64 nm. PDI: 0.1–0.4. ZP: −20.68 to −10.93 mV. LC: 83.24–95.60%.	The optimal Cur-Bet-IL showed the lowest viscosity among the tested molar ratios, approximately 70 mPa·s at room temperature. Thermal analysis showed Tg values around 81–83 °C and T_dec_ values around 266–283 °C depending on Cur-Bet-IL ratio; the 1:1 Cur-Bet-IL showed Tg = 81.12 °C and T_dec_ = 281.17 °C. Cur-Bet-IL-Lip showed a Tyndall effect, TEM-confirmed nanosized morphology and short-term stability for 10 days at room temperature without major size/PDI changes.	[206]
5	Transferosome in hydrogel	Direct dermal evidence Formulation performance	Transdermal systemic delivery for rheumatoid arthritis-oriented anti-inflammatory therapy; designed to overcome oral Cur bioavailability limitations rather than to treat a local dermal disorder.	Transfersomes: SL, Chol, Tween 80/Plurol Oleique CC-497 (PO). Hydrogel: Carbopol 934, polyethylene glycol, methylparaben.	Thin-film hydration method. Formal optimization was performed by central composite design. Factors: phospholipid concentration and edge activator concentration. Responses: particle size, zeta potential, and EE. Twelve experimental runs were generated.	Size: 128–503 nm. PDI: 0.365 (Only for optimal formulation). ZP: −19.2 to −54.5 mV. EE: 74.3 ± 0.14% to 90.6 ± 0.19%.	TEM showed almost spherical, well-distributed vesicles with visible vesicular boundaries. DSC showed disappearance of the Cur endothermic peak in the optimized formulation, suggesting amorphization/entrapment within vesicles. FTIR supported compatibility between Cur and excipients. Gel was yellow, smooth, homogeneous, pH 5.9 ± 0.12, drug content 82.4 ± 2.09%, spreadability 32.19 ± 1.09 g·cm/min, and viscosity 1350 ± 22.2 cP. Stability over 90 days: release decreased by 1.45% at 25 °C and 2.65% at 40 °C, while EE decreased by 0.47% and 1.83%, respectively.	[207]
6	Niosome in hydrogel	Skin-related biological evidence Formulation performance	Acne-oriented topical antibacterial gel. *C. xanthorrhiza* extract-loaded niosomes; not isolated CUR. The extract contains curcumin plus other curcuminoids and xanthorrhizol-rich essential oils.	Niosomes: Chol, Span 60. Hydrogel: Carbopol 940, TEA, propylene glycol, methylparaben.	Thin-film hydration method. No formal DoE; empirical comparison of Span 60 concentration with fixed Chol and extract content. The best formulation was selected based on EE, size, ZP, release profile, and antibacterial activity.	Size: 10.58 ± 0.01 µm to 14.52 ± 0.07 µm. PD: NR. ZP: −79.3 ± 0.1 mV to −72.7 ± 0.1 mV. EE: 94.89 ± 0.08%–95.27 ± 0.11%.	FTIR showed O–H and C=C bands similar to blank niosomes, supporting extract encapsulation without major detectable interaction. SEM showed spherical-to-slightly irregular vesicular morphology, with extract-loaded niosomes showing minor aggregation and less regular shape than blank vesicles. The best formulation showed pH 6.97 ± 0.04. The selected niosomal gel remained organoleptically stable and homogeneous after freeze–thaw cycling. pH showed a minimal increase of 0.38%. In contrast, viscosity decreased by 8.78%, while spreadability increased by 12.36%.	[208]
7	Niosome and hyaluronan niosome (HN)	Skin-related biological evidence Formulation performance	Antioxidant and anti-inflammatory co-delivery system. In vivo biological evaluation used oral administration before carrageenan-induced paw edema, with histological analysis of dermis/hypodermis inflammation.	Span 20, 40 and 60, Chol, Tween 80, hyaluronic acid, co-encapsulation with quercetin (Quer).	Modified thin-film hydration method. Formal Box–Behnken design: 3 factors, 3 levels, 15 runs. Factors: surfactant HLB, Chol concentration, hyaluronan amount. Responses: size, PDI, ZP, EE-Cur, EE-Quer.	Size: 221.70 ± 2.69 nm–788.67 ± 14.57 nm, PDI: 0.32 ± 0.01–0.64 ± 0.04. ZP: −40 ± 1.18 mV to −21.33 ± 1.63 mV. EE: 75.52 ± 4.77%–99.07 ± 0.79% (Cur); 17.33 ± 7.34–95.50 ± 2.46% (Quer).	TEM and AFM showed spherical vesicles. CTAB turbidimetric assay confirmed hyaluronan association with vesicles; HA encapsulation 58.79 ± 1.32% and HA loading 18.64 ± 1.99%. DSC and PXRD indicated disappearance of Cur/Quer crystalline peaks, suggesting drug incorporation in amorphous or molecularly dispersed form. After 90 days at room temperature, hyaluronan-containing niosomes retained size, PDI, ZP and EE reasonably well; conventional niosomes showed drug precipitation after 2 weeks and size increased to about 422.70 nm.	[209]
8	Niosome in hydrogel	Direct dermal evidence Skin-related biological evidence Formulation performance	Dermal/transdermal delivery of Cur as a local anti-inflammatory and antinociceptive agent; compared with conventional vehicles including ethanol, polyethylene glycol, PEG 200 and PEG 400	Niosomes: Span 20, Tween 20, Chol. Hydrogel: Carbopol 941, TEA.	Thin-film hydration method. No formal DoE; formulation optimization was based on Chol:surfactant ratio screening and comparison of size, PDI, ZP, EE, release, permeation and skin retention.	Size: 306.63 ± 6.76–444.27 ± 4.86 nm. PDI: 0.283 ± 0.009–0.499 ± 0.005. ZP: −9.31 ± 0.97 to −0.73 ± 0.31 Mv. EE: 87.42 ± 0.41%–95.19 ± 0.08%.	SEM showed spherical and separated vesicles. ATR-FTIR ruled out major chemical interaction between Cur and excipients. DSC showed disappearance of the Cur melting peak in niosomes, suggesting amorphization. PXRD confirmed disappearance of most diagnostic crystalline Cur peaks, indicating that Cur was molecularly dispersed or present in an amorphous state within niosomes.	[210]
9	Ethosome	Direct dermal evidence Skin-related biological evidence Formulation performance	Transdermal Cur delivery; designed to balance ethosomal flexibility, skin permeability and vesicle stability through PC/HPC phospholipid composition	PC, HPC, Chol, ethanol.	Ethanol injection method. No formal DoE; rational comparative design based on PC/HPC ratio, supported by molecular simulation, DSC and MDA peroxidation assay.	Size: 132.37 ± 1.53 nm to 256.70 ± 11.48 nm. PDI: 0.04 ± 0.01–0.50 ± 0.07 ZP: −13.40 ± 0.56 mV to −8.47 ± 1.02 mV. EE: 93.76 ± 1.25%–97.26 ± 1.33%.	PC contributed membrane fluidity and faster Cur release but increased susceptibility to phospholipid peroxidation. HPC increased bilayer rigidity and reduced PC peroxidation, improving vesicle stability; however, excessive HPC reduced deformability, increased vesicle size/PDI and impaired skin permeation. The intermediate PC/HPC ratio provided a rigidity–flexibility balance that favored stable nanosized ethosomes, sustained Cur release and enhanced SC interaction.	[211]
10	Ethosome, functionalized with TPGS and glycyrrhetinic acid (GA)	Direct dermal evidence Skin-related biological evidence Formulation performance	Topical psoriasis therapy; designed to integrate Cur-mediated anti-inflammatory/antioxidant activity, GA glucocorticoid-like action and TPGS-assisted anti-lipid-peroxidation/permeation enhancement.	S100 phospholipid, ethanol, GA-TPGS.	Ethanol injection method. No formal DoE. The formulation logic was mechanistic rather than statistical: first, GA was conjugated to TPGS to solve the polarity mismatch between CUR and GA; second, the CUR:GA-TPGS ratio was selected using an IL-6-induced HaCaT inflammatory proliferation model; third, the optimized vesicles were evaluated for transdermal delivery and antipsoriatic efficacy.	Size: 168.9 ± 15.6 nm. PDI: 0.130 ± 0.009. ZP: NR. EE: NR.	The main formulation event is the conversion of GA into a membrane-compatible amphiphilic conjugate. This allowed GA to function as a surface-associated therapeutic/permeation modifier rather than only as a co-dissolved drug. Cur crystallinity disappeared by DSC/XRD, indicating amorphization or molecular dispersion inside the vesicular matrix, which likely favored release and skin partitioning.	[212]
11	Ethosome in hydrogel	Direct dermal evidence Skin-related biological evidence Formulation performance	Topical anti-inflammatory therapy; designed to improve Cur solubilization, skin diffusion, semisolid residence, and local anti-inflammatory activity.	Ethosome: SL, ethanol. Hydrogel: gelatin type A, Carbopol 940, TEA.	Hydroalcoholic dispersion/probe-sonication method. No formal DoE; empirical screening of lecithin content and gel composition.	Size: 122.08 ± 9.66 nm PDI: NR. ZP: −36.2 Mv. EE: 89.52 ± 0.1–0.33%.	The formulation integrates a nanosized ethosomal compartment with a gelatin/Carbopol semisolid matrix. Ethanol and lecithin likely improved Cur solubilization and lipid-domain partitioning, whereas gelatin may have reduced drug leakage and contributed to sustained release. Carbopol converted the dispersion into a topical dosage form with skin-compatible pH, high viscosity, and adequate spreadability.	[213]
12	Transethosome	Direct dermal evidence Skin-related biological evidence Formulation performance	Topical wound-healing platform; designed to increase local Cur deposition in skin layers while reducing cytotoxicity and preserving antimicrobial activity against wound-associated bacteria.	SPC, Tween 80, ethanol.	Thin-film hydration method. No formal DoE; the study used a fixed optimized composition based on the transethosome rationale: phospholipid bilayer + ethanol + Tween 80 as edge activator.	Size: 115.75 ± 4.88 nm. PDI: 0.127 ± 0.0219. ZP: −5.74 ± 0.47 mV. EE: 99.7 ± 0.09%.	The system combines ethosome-like ethanol-mediated lipid fluidization with transferosome-like deformability from Tween 80. Although ZP was only mildly negative, the low PDI, nanosize, and Tween 80-mediated steric stabilization supported colloidal stability over refrigerated storage. DSC showed disappearance of the Cur endothermic peak, indicating amorphization or molecular dispersion in the lipid matrix. TEM supported spherical vesicles with no evident fusion, reinforcing the role of surfactant/ethanol organization in maintaining a deformable nanosized carrier. After 3 months at 4 °C, vesicle size increased by 13.99%, while PDI increased by 13.39%. Zeta potential became less negative, with its absolute magnitude decreasing by 16.20%.	[214]
13	Ethosome and transferosome	Skin-related biological evidence Formulation performance	Dermal/topical antineoplastic platform for Cur delivery, with biological evaluation in HUT-78 cutaneous T-cell lymphoma cells. The study is relevant to skin oncology but does not provide direct skin delivery evidence.	Ethosomes: Lipoid S75, ethanol. Transferosomes: Lipoid S75, Tween 20, Tween 60, Tween 80.	Thin-film hydration method. No formal DoE; comparative formulation screening based on ethanol concentration, phospholipid concentration, edge activator type, and edge activator concentration.	Ethosome: Size 578.6 ± 4.54 nm–871.1 ± 16.34 nm. PDI: 0.16 ± 0.06–0.41 ± 0.03. ZP: −42.14 ± 4.67 to −65.96 ± 3.60 mV. EE: 69.1 ± 2.21%–81.5 ± 1.61%. Transferosomes: Size: 787 ± 10.46 nm–1383 ± 58.62 nm. PDI: 0.31 ± 0.05–0.75 ± 0.14. ZP: −56.12 ± 1.18 to −70.31 ± 1.45 mV. EE: 57.5 ± 2.17%–70.7 ± 2.35%.	Ethanol concentration controlled ethosomal size, charge, and Cur accommodation. Increasing ethanol reduced vesicle size and made ZP more negative, but excessive ethanol likely destabilized the vesicular membrane and reduced EE through drug leakage. Increasing phospholipid content reduced ethosome size and improved charge stabilization, but Cur entrapment did not increase linearly, suggesting a saturation limit in the lipid bilayer. In transfersomes, edge activator chemistry was decisive: Tween 60 favored Cur entrapment, whereas Tween 80 favored smaller vesicles, probably due to unsaturation-driven changes in bilayer packing.	[57]
Chlorogenic acid (CGA)								
14	Transferosome and protransferosome, in hydrogel	Direct dermal evidence Skin-related biological evidence Formulation performance	Topical/transdermal wound-healing platform for CGA, with antioxidant, antibacterial and excision wound-healing evaluation.	Transferosome: Lipoid S100, Tween 80. Protransferosome: Lipoid S100, Span 60, ethanol. Hydrogel: Carbopol 934, propylene glycol, TEA.	Thin-film hydration method. Central composite design; 20 runs for each system. Variables included phospholipid, surfactant and sonication time or ethanol amount.	Transferosome: Size: 584.5 ± 27 nm. PDI: 0.517. ZP: −52.3 ± 0.06 to −20.9 ± 0.49. EE: 55.6 ± 0.99–99.41 ± 0.77. Protransferosome: Size: 315.6 ± 13 nm. PDI: 0.423. ZP: −39.4 ± 0.29 to −31.2 ± 0.04. EE: 81.13 ± 0.58–98.25 ± 0.04.	Tween 80-containing transfersomes were larger and more polydisperse, but functionally more deformable and permeation-efficient. Span 60-containing protransferosomes generated smaller, more negatively charged vesicles, but their higher bilayer rigidity appears to reduce flux relative to Tween 80 systems.	[215]
15	Niosomes and proniosomes in hydrogel.	Direct dermal evidence Skin-related biological evidence Formulation performance	Topical wound-healing system for CGA; combines permeation enhancement, antioxidant activity, antibacterial action and wound contraction.	Niosomes: Chol, Tween 80. Proniosomes: Chol, Lipoid S100, Span 60. Hydrogel: Carbopol 934, propylene glycol, TEA.	Niosomes by thin-film hydration method and proniosomes by phase-separation coacervation. Central composite design; 20 runs for each system. Optimization focuses on lipid/surfactant composition and sonication or phospholipid-related variables.	Niosomes: Size: 490.1 ± 43.0 nm. PDI: 0.526. ZP: −29.80 ± 0.74 to −21.00 ± 0.46. EE: 78.00 ± 0.54–99.72 ± 0.25. Deformability: 8.77 ± 0.22. Proniosomes: Size: 280.0 ± 22.0 nm. PDI: 0.173. ZP: −39.90 ± 0.98 to −26.00 ± 0.29. EE: 31.60 ± 0.32–97.27 ± 0.25. Deformability 6.87 ± 0.17.	Proniosomes generated smaller and much more monodisperse vesicles, supporting better physical organization and topical handling. Niosomes showed a higher deformability and permeability coefficient, suggesting that flexibility, not only size, governed skin transport.	[56]
16	Liposomes, modified with sialic acid (SA) and combined with anti-PD-1.	Skin-related biological evidence Formulation performance	Systemic melanoma immunotherapy. Relevant to dermato-oncology because the tumor model is melanoma, but not a dermal/topical delivery study.	HSPC, Chol, and SA-ODA.	Ethanol injection method. Sialic acid was coupled to octadecylamine to generate a lipophilic targeting motif for TAM-associated uptake.	Liposome: Size: 87.00 ± 1.29 nm. PDI: 0.251 ± 0.005. ZP: +14.6 ± 0.6 mV. EE: 51.47 ± 3.08%. Liposome modified: Size: 90.36 ± 0.54 nm. PDI: 0.254 ± 0.006. ZP: +13.8 ± 0.1 mV. EE 49.84 ± 2.96%.	Sialic acid modification did not meaningfully change size or morphology but changed biological targeting. The vesicle surface became a TAM-interaction module rather than only a CGA carrier.	[216]

Abbreviations: Cur: curcumin; CGA: chlorogenic acid; SPC: soy phosphatidylcholine; SL: soy lecithin; Chol: cholesterol; PAM: polyacrylamide; CaCl_2_: calcium chloride; DoE: design of experiments; PDI: polydispersity index; ZP: zeta potential; NR: not reported; EE: encapsulation efficiency; UV–Vis: ultraviolet–visible spectroscopy; λmax: maximum absorption wavelength; TGA: thermogravimetric analysis; FTIR: Fourier transform infrared spectroscopy; EL: egg lecithin; Ibr: ibrutinib; TEA: triethanolamine; TEM: transmission electron microscopy; RH: relative humidity; NDLs: neutral deformable liposomes; P20: Polysorbate 20/Tween 20; SA: stearylamine/sialic acid, depending on context; CDL/CDLs: cationic deformable liposome(s); SDC: sodium deoxycholate; ADL/ADLs: anionic deformable liposome(s); EI: elasticity index; Cur-Bet-IL: curcumin–betaine ionic liquid; LC: loading capacity; Tg: glass transition temperature; T_dec_: decomposition temperature; Lip: liposome; PO: Plurol Oleique CC-497; DSC: differential scanning calorimetry; cP: centipoise; PD: polydispersity; SEM: scanning electron microscopy; HN: hyaluronan niosome; Quer: quercetin; HLB: hydrophilic–lipophilic balance; AFM: atomic force microscopy; CTAB: cetyltrimethylammonium bromide; HA: hyaluronic acid/hyaluronan; PXRD: powder X-ray diffraction; PEG: polyethylene glycol; ATR-FTIR: attenuated total reflectance Fourier transform infrared spectroscopy; PC: phosphatidylcholine; HPC: hydrogenated phosphatidylcholine; MDA: malondialdehyde; SC: stratum corneum; TPGS: D-α-tocopheryl polyethylene glycol succinate; GA: glycyrrhetinic acid; S100: Lipoid S100 phospholipid; IL-6: interleukin-6; HaCaT: human immortalized keratinocyte cell line; XRD: X-ray diffraction; HUT-78: human cutaneous T-cell lymphoma cell line; TAM: tumor-associated macrophage; anti-PD-1: anti-programmed cell death protein 1 antibody; HSPC: hydrogenated soy phosphatidylcholine; SA-ODA: sialic acid–octadecylamine conjugate.

**Table 3:** Release, skin delivery performance, biological activity, and translational limitations of antioxidant-loaded vesicular systems.

N°	Release Behavior	Dermal/Biological Performance	Main Physicochemical or Mechanistic Insight	Translational Relevance/Limitation	Ref.
1	Lipogel showed sustained Cur release compared with bare Cur hydrogel. Release increased with Cur loading. Higuchi and Korsmeyer–Peppas provided the best fit, and reported Fickian diffusion	DPPH radical scavenging increased with Cur loading (~70.87–91.02%). Antibacterial activity increased with Cur-loaded lipogel concentration, with maximum inhibition zone reported as 25 mm against both *E. coli* and *S. aureus*. WST-1 assay showed >50% viability for all formulations, suggesting non-cytotoxicity under the tested conditions. CAM assay with one formulation showed enhanced angiogenic response, with 203 veins, 8090 total nodes, and 295 secondary nodes reported.	Liposomal encapsulation improved Cur dispersion/solubility and enabled sustained diffusion from a crosslinked hydrogel matrix. The PAM/alginate network acted as a secondary release-regulating and wound-dressing scaffold, while the liposomal compartment contributed to Cur loading, antioxidant availability, and antibacterial performance.	Relevant as a semisolid dermal vesicular platform for wound healing; however, evidence is limited by absence of zeta potential, morphology by TEM/SEM of vesicles, skin permeation/retention studies, ex vivo or in vivo wound model, long-term stability, and irritation testing. The vesicles were relatively large and polydisperse, and organic solvent removal/scalability should be considered for translation.	[203]
2	Plain drugs showed complete release within 8 h, whereas liposomes delivered approximately 60% of the drug in a controlled manner over 12 h. Lipogel diffusion exceeded 60% within 12 h. Release from gel followed zero-order kinetics and Korsmeyer–Peppas model for Cur, and reported diffusion-controlled release.	Skin model assay: Ex vivo permeation using BALB/c mouse dorsal skin in Franz diffusion cells. Lipogel increased permeation by 3.87-fold for Ibr and 3.4-fold for Cur compared with plain drug gel. Reported 24 h flux (J) values were 6.13 ± 0.43 g/h.cm^2^ and 7.43 ± 0.76 g/h.cm^2^ for Ibr and Cur from lipogel, compared with 2.49 ± 0.01 g/h.cm^2^ and 1.85 ± 0.199 g/h.cm^2^ for Ibr and Cur from plain drug gel, respectively. Ibr/Cur liposomes showed stronger synergism than free drug combination. In HaCaT cells, IC50 decreased from 3.09 ± 0.77 μM to 1.395 ± 0.95 μM for Ibr after liposomal loading and from 6.90 ± 0.36 μM to 3.86 ± 0.67 μM for Cur. In IMQ-induced psoriasis, the Cur-Ibr lipogel reduced PASI scores, erythema, scaling, skin thickening, ear thickness, spleen/body weight ratio, epidermal hyperplasia, acanthosis, and inflammatory cytokines. Compared with the negative control, IBC liposomal gel reduced IL-22, TNF-α, IL-17, IL-23, IL-6, and IL-2 by 2.00-, 3.69-, 2.34-, 3.78-, 4.00-, and 3.21-fold, respectively	Small liposome size, low PDI, high EE, and Carbopol-mediated bioadhesion collectively improved topical delivery. Liposomal co-encapsulation favored controlled release, enhanced permeation, better dermal fluorescence distribution, and stronger anti-inflammatory efficacy than plain drug gel or individual liposomal drugs. The study supports a structure–performance relationship in which nanosized EL/Chol liposomes improve drug partitioning and penetration, while Carbopol prolongs residence on psoriatic skin.	Highly relevant as direct dermal evidence for Cur-containing lipogel in psoriasis. However, Cur was not evaluated as the only active in the final optimized therapeutic system; the main efficacy depends on co-delivery with Ibr. Limitations include short stability study, no quantitative skin retention, no deformability or lamellarity analysis, limited chronic irritation assessment, and need for clinical validation, especially because topical Ibr introduces regulatory and safety considerations beyond cosmetic antioxidant delivery.	[204]
3	No conventional in vitro release assay was reported. Performance was inferred from ex vivo human skin penetration profiles over 24 h. CDLs produced the most sustained Cur penetration, whereas ADLs showed the highest early penetration enhancement.	Skin model assay: ex vivo full-thickness human abdominal skin in Franz diffusion cells. Biological assays included LPS-induced RAW 264.7 macrophages for NO inhibition, HFF fibroblast viability, and antibacterial testing against Staphylococcus aureus and Streptococcus pyogenes Cur in propylene glycol solution (control) produced 6.80 ± 0.77 µg/cm^2^ penetration. All Cur-transferosomes enabled measurable Cur penetration through full-thickness human skin. ADLs showed the highest penetration enhancement, while CDLs showed the most sustained profile. ADLs reached the highest cumulative levels during the 4–8 h interval, whereas CDLs maintained a more gradual penetration profile up to 24 h. All Cur-transferosomes inhibited NO production in LPS-induced macrophages in a concentration-dependent manner and were more active than control. CDLs produced the strongest anti-inflammatory effect, inhibiting NO production by approximately 10% more than NDLs and ADLs at the highest lipid concentration. All formulations were non-cytotoxic to HFF fibroblasts after 12 and 24 h and showed proliferative effects. All Cur-transferosomes inhibited *S. aureus* and *S. pyogenes* growth, with CDLs showing the strongest antibacterial effect, superior to free Cur solution.	Surface charge was the main differentiating variable because Cur-transferosomes had similar deformability but different skin penetration and biological activity. Cationic vesicles likely improved electrostatic interaction with negatively charged skin cells and bacterial membranes, explaining sustained dermal penetration, stronger NO inhibition, and superior antibacterial activity. ADLs enhanced penetration more strongly, possibly due to SDC-mediated membrane interaction and high Cur/lipid loading.	Strong direct dermal evidence using full-thickness human skin and mechanistic comparison of surface charge. Relevant for wound-oriented dermal therapy. Limitations include no formal release assay, no quantitative skin retention by layer, no in vivo wound model, no long-term storage stability, and no clinical tolerability assessment. The term “deformable liposomes” should be interpreted as transferosome-like ultra deformable vesicles due to the presence of edge activators.	[205]
4	pH-dependent release. At pH 5.0, cumulative curcumin release was approximately 60%; at pH 7.4, cumulative release |was approximately 40%, indicating faster release under mildly acidic conditions.	Skin model assay: Franz diffusion cells using treated animal skin (mouse and *Sus scrofa domestic* skin). Skin was evaluated by fluorescence imaging, H&E, immunofluorescence, SEM, and layer-specific curcumin content. Cellular assays used HaCaT keratinocytes and HSF fibroblasts. At 24 h, cumulative permeation was 49% for Cur-Bet-IL-Lip versus 36% for Cur-Bet-IL. Fluorescence intensity after 24 h was 2.64-fold higher than PBS. Cur-Bet-IL-Lip penetrated the stratum corneum and delivered CUR to epidermis and dermis. Cur was detected in stratum corneum, epidermis, and dermis after 24 h. Cur-Bet-IL-Lip showed higher total skin deposition than Cur-Bet-IL and PBS, with appreciable distribution into epidermis and dermis. H&E showed preserved skin integrity after permeation, and SEM revealed small particles on the skin surface after Cur-Bet-IL and Cur-Bet-IL-Lip treatment, interpreted as Cur-Bet-IL-related residues. Cur-Bet-IL-Lip increased HaCaT uptake compared with Cur-Bet-IL; fluorescence uptake was 1.87-fold higher. Uptake increased after 12 h. Cur-Bet-IL-Lip inhibited HaCaT proliferation more strongly than Cur-Bet-IL, especially under TNF-α-stimulated psoriasis-like conditions. It reduced TNF-α, IL-1β, IL-17A, IL-17F, and IL-22 expression, and increased collagen-I expression. HSF viability indicated comparatively low toxicity/acceptable biocompatibility.	The system integrates ionic-liquid derivatization and liposomal encapsulation. Curcumin succinate–betaine interaction improved Cur-Bet-IL formation, while liposomal incorporation improved nanoscale delivery, colloidal stability, skin permeation, and HaCaT uptake. The 1:1:1 SL/Chol/Cur-Bet-IL ratio offered the best balance between loading capacity, size, and negative zeta potential. Increasing Cur-Bet-IL proportion increased vesicle size and made zeta potential less negative while reducing the loading capacity after the 1:1:1 optimum.	Relevant as a hybrid chemical–vesicular strategy for psoriasis-oriented transdermal Cur delivery. However, it uses a Cur derivative rather than native Cur, so it should not be compared directly with conventional Cur-loaded liposomes without clarification. Limitations include no in vivo psoriasis model, no quantitative flux/Jss or Kp, short-term stability only.	[206]
5	Transfersome released ~62–77% Cur over 24 h. For gel comparison, drug-loaded gel released 24.2% in 24 h, whereas transfersome-loaded gel released 54.4% in 24 h.	Skin model assay: ex vivo permeation using full-thickness abdominal skin from healthy male Wistar rats mounted in modified Franz diffusion cells. At 24 h, cumulative permeation was 62.4% for transfersome-loaded gel versus 24.3% for drug-loaded gel. The authors report ~2.5-fold higher transdermal flux. Skin irritation study showed no erythema, edema, or inflammation after transfersome gel application for one week. Pharmacokinetic study showed enhanced systemic exposure compared with oral Cur suspension: C_max_ 3.823 vs. 4.001 µg/mL; T_max_ 2 vs. 1 h; t1/2 4 vs. 1.5 h; AUC_0–t_ 24.30 vs. 13.46 µg·h/mL; AUC_0–∞_ 30.69 vs. 14.11 µg·h/mL; MRT_0–∞_ 7.777 vs. 3.48 h for transdermal transfersome gel versus oral suspension, respectively.	The combination of SL, Chol, and Tween 80/PO generated flexible vesicles with electrostatic and steric stabilization. Edge activators likely increased bilayer deformability and acted as permeation enhancers by modifying SC lipid organization. Carbopol gel provided a semisolid vehicle suitable for skin residence and application. Improved permeation and prolonged plasma exposure suggest that transfersomes converted Cur delivery from rapid oral exposure into slower, sustained transdermal systemic input.	Strong formulation and transdermal evidence because the study includes CCD optimization, ex vivo permeation, 90-day stability, skin irritation, and in vivo pharmacokinetics. However, it is less directly aligned with cosmetic or local dermal therapy because the target is systemic delivery for rheumatoid arthritis. Limitations include absence of direct rheumatoid arthritis efficacy testing in this study, no skin-layer retention data, no deformability index.	[207]
6	The selected formulation showed the most prolonged release and best fit to the Higuchi model. For gels, cumulative curcumin release after 8 h was 30.60 ± 2.84 µg/cm^2^ for niosomal gel and 36.57 ± 7.99 µg/cm^2^ for extract gel. Niosomal gel showed lower total release but a more stable and sustained profile. Release kinetics for niosomal gel followed Korsmeyer–Peppas and reported non-Fickian transport involving diffusion and polymer relaxation.	No skin model. In vitro release was evaluated using dialysis membrane in Franz diffusion cells. Antibacterial activity was evaluated by broth microdilution for niosomes and disc diffusion for gels against *Propionibacterium acnes*, *Staphylococcus epidermidis*, and *Staphylococcus aureus*. Not reported as dermal permeation. The study reports release/flux across dialysis membrane, not skin. Niosomal gel showed a more stable flux profile than extract gel, which showed burst release followed by rapid decline. Niosome selected formula showed MIC/MBC values of 78.12/156.25 µg/mL against *P. acnes*, 312.50/312.50 µg/mL against *S. epidermidis*, and 312.50/312.50 µg/mL against *S. aureus*. Blank niosomes showed no antibacterial activity. Extract alone showed MIC/MBC values of 156.25/156.25 µg/mL for *P. acnes*, 156.25/312.50 µg/mL for *S. epidermidis*, and 312.50/1250 µg/mL for *S. aureus*. Niosomal gel inhibited *P. acnes*, *S. epidermidis*, and *S. aureus*, with inhibition zones reported as ~14.75, ~11.5, and ~9.83 mm, respectively.	Span 60 and cholesterol formed stable non-ionic surfactant vesicles with very high EE and strongly negative ζ-potential. The selected formulation offered the best balance between size, EE, release control, and antibacterial performance. The micrometric multilamellar structure and Chol-containing bilayer likely slowed extract diffusion and reduced burst release. Incorporation into Carbopol 940 gel further converted the dispersion into a topical platform with improved handling, sustained release, and antibacterial activity.	Relevant as a topical antibacterial niosomal gel for acne-associated pathogens. However, it should not be treated as direct dermal permeation evidence because no skin or skin-equivalent membrane was used. Limitations include micrometric vesicle size, absence of PDI, absence of skin permeation/retention, no in vivo acne or irritation model, short-term freeze–thaw stability only, lack of curcuminoid standardization by HPLC in the formulation, and use of a botanical extract rather than purified curcumin.	[208]
7	HN released both drugs more slowly than conventional niosomes. Quer release from both systems showed an initial burst followed by slower release; complete Quer release occurred over about 41 h from HN versus about 18 h from conventional niosomes. Cur release was slower and more prolonged than Quer. Best-fit models: Quer from HN and conventional niosomes; Cue from HN, non-conventional order 2, and Cur from conventional niosomes, three-second root-of-mass model.	No dermal permeation model. Antioxidant activity was evaluated by DPPH assay. Anti-inflammatory activity was assessed in female rats using carrageenan-induced paw edema after oral administration of formulations at 10 mL/kg. Paw volume was measured up to 24 h; paw skin/tissue samples were fixed, sectioned, H&E-stained and evaluated for edema and inflammatory cell infiltration in dermis and hypodermis. HN showed stronger DPPH scavenging than conventional niosomes, reaching approximately complete scavenging at lower formulation volume. In Vivo, HN produced significantly higher inhibition of carrageenan-induced edema than conventional niosomes and simple Cur/Quer suspension across all time points. Histology showed lower edema and inflammatory cell infiltration in the HN-treated group than in untreated carrageenan, conventional niosome, empty HN, or simple suspension groups.	Hyaluronan improved vesicular performance by reducing particle size, increasing Cur/Quer entrapment, improving colloidal stability, reducing drug leakage, and slowing release. The HA gel network likely hosted hydrophobic drugs during hydration and contributed to bilayer stiffness/membrane stabilization. Improved antioxidant and anti-inflammatory activity were associated with higher drug entrapment, better stability, and more sustained release.	Valuable as a formulation and biological precedent for Cur/Quer co-loaded niosomes with hyaluronic acid, especially for antioxidant and anti-inflammatory applications. However, it is not direct dermal delivery evidence: no topical gel, no Franz skin permeation, no skin retention, no follicular delivery, no irritation/tolerability assay, and the in vivo anti-inflammatory study used oral administration.	[209]
8	In vitro release was evaluated using immersion cells with a cellulose acetate membrane. Increasing cholesterol content increased the Cur dissolution/release from niosomes. All Cur-niosome formulations enhanced Cur release compared with the Cur solution control.	Skin model assay: ex vivo permeation through excised abdominal skin from male Wistar rats mounted in Franz diffusion cells. Niosomes produced significantly higher Cur permeation than conventional vehicles. Maximum receptor amount among controls was obtained with Cur-PG solution: 0.27 ± 0.23% or 7.35 ± 3.34 µg/cm^2^. The best formulation produced the highest transdermal permeation: 1.16 ± 0.018% or 30.67 ± 0.67 µg/cm^2^. Niosomes increased Cur deposition in skin compared with conventional vehicles. Maximum skin retention among controls was Cur-PG solution: 2.85 ± 0.24% or 72.5 ± 9.32 µg/cm^2^. The best formulation produced the highest dermal retention: 4.57 ± 0.05% or 120.43 ± 1.64 µg/cm^2^. HFF fibroblast viability remained around or above 90% after 24 h exposure to free Cur, blank niosomes, or the optimized formulation. The optimized niosomal gel was classified as non-irritant, with a skin irritation score of 1. It also showed stronger antinociceptive/anti-inflammatory activity than Cur gel in the late phase of the formalin test and significantly increased tail-flick latency, especially after 30 min.	Chol: surfactant ratio was the main driver of performance. Increasing Chol increased EE and improved release, dermal retention and transdermal permeation, probably by modifying bilayer rigidity, Cur localization, vesicle–stratum corneum interaction and membrane permeability. The optimized formulation gave the best functional performance despite its near-neutral ZP and relatively high PDI.	Strong direct dermal evidence because the study includes rat-skin permeation, skin retention, irritation, cytotoxicity and in vivo topical antinociceptive/anti-inflammatory assays. Limitations include no human or porcine skin model, no long-term stability data, no formal DoE, no lamellarity analysis, relatively high PDI for the optimized formulation, and near-neutral zeta potential that may compromise colloidal stability before gel incorporation.	[210]
9	In vitro release was evaluated for 24 h using dialysis membrane in Franz diffusion cells. PC increased membrane fluidity and Cur release, whereas HPC slowed release by increasing bilayer rigidity. However, excessive HPC reduced deformability and permeation; thus, controlled release was beneficial only when coupled with sufficient vesicle flexibility.	Skin model assay: ex vivo permeation through full-thickness rat abdominal skin using Franz diffusion cells. Flux: free Cur 8.88 ± 0.93 ng/cm^2^/h; Formulations: Transdermal flux ranged from 8.30 ± 2.67 to 19.54 ± 4.14 ng/cm^2^/h, with ER values between 0.93 and 2.20. No quantitative dermis retention was reported. Fluorescence imaging suggests a composition-dependent deposition pattern: PC-rich ethosomes mainly favored superficial SC localization, HPC-rich ethosomes showed limited skin penetration, and the balanced PC/HPC system promoted deeper and follicular-associated delivery by combining bilayer stability with sufficient deformability. HaCaT uptake data suggest that balanced PC/HPC ethosomes enhanced skin transport without promoting excessive keratinocyte internalization, supporting a preferential intercellular/follicular pathway and potentially lower local cytotoxicity.	The dermal performance was governed by the balance between ethanol-induced vesicle flexibility and HPC-induced bilayer stabilization. A moderate HPC fraction protected unsaturated PC from oxidative degradation and stabilized the ethosomal bilayer, while preserving enough deformability to disturb SC lipid domains and promote follicular/deep-skin penetration. PC-rich vesicles favored faster release but lower oxidative stability, whereas HPC-rich vesicles became larger, less deformable and less efficient for permeation.	Strong direct dermal evidence for Cur-loaded ethosomes because it combines permeability, flux, deformability, stability and mechanistic SC analysis. Limitations include no in vivo pharmacodynamic efficacy model, no long-term stability beyond 28 days, no quantitative skin-layer retention, use of rat rather than human/porcine skin, and no direct antioxidant or anti-inflammatory biological assay.	[211]
10	No isolated in vitro release profile was central to the study. The relevant release evidence comes from in vivo dermal microdialysis: the vesicles delayed Tmax and increased MRT for both drugs, indicating that the ethosomal system behaved as a dermal reservoir rather than as a simple ethanol vehicle. This sustained input is important because psoriasis requires prolonged local modulation of inflammation, not only rapid permeation.	Skin model assay: ex vivo permeation through full-thickness rat abdominal skin in Franz cells. Functionalized ethosomes increased 24 h percutaneous penetration relative to ethanol solution: Cur 2.88-fold and GA 1.93-fold. In vivo microdialysis confirmed stronger dermal exposure: C_max_ increased 4.09-fold for Cur and 2.14-fold for GA; AUC_0–t_ increased 4.43-fold for Cur and 2.06-fold for GA. The smaller enhancement for GA suggests that the GA-TPGS conjugate improved co-delivery, but its hydrophilic/PEGylated character still limited its skin transport relative to Cur. CLSM showed stronger fluorescence throughout epidermal and dermal regions than free probes, suggesting that the functionalized ethosomes increased both penetration depth and local deposition. The simultaneous presence of shell-associated and payload-associated fluorescence supports carrier-mediated co-distribution rather than independent diffusion of free compounds. In IL-6-stimulated HaCaT cells, the functionalized ethosomes reduced inflammatory proliferation, downregulated p-IκBα, p-p65 and p-STAT3, and lowered ROS more effectively than non-functionalized or ethanol-based controls. In IMQ-induced psoriasis, topical treatment reduced PASI progression, epidermal hyperplasia, inflammatory infiltration, NF-κB/ROR-γt expression, IL-17 and IL-23, while increasing GR-α and antioxidant defenses such as SOD and CAT.	The performance arises from functional integration of carrier and pharmacology. Ethanolized phospholipid vesicles increased SC interaction and penetration. TPGS improved deformability/permeation and contributed antioxidant protection. GA-TPGS localized a glucocorticoid-like anti-inflammatory motif at the vesicle interface. Cur supplied intracellular anti-inflammatory/antioxidant activity. The system therefore acts as a co-delivery platform and not merely as a solubility enhancer.	Strong dermal evidence because it links physicochemical design, ex vivo permeation, in vivo dermal exposure and antipsoriatic efficacy. Limitations: no human/porcine skin, no zeta potential or EE reported, receptor medium contained ethanol/PEG 400, long-term stability was not conducted, and the use of a synthetic GA-TPGS conjugate adds regulatory/safety complexity.	[212]
11	The release profile suggests a dual-control system: an initially accessible Cur fraction associated with phospholipid/ethanol domains, followed by slower diffusion through the vesicular bilayer and polymeric gel network. Reported 24 h release was high, although values varied depending on the comparison: 95.19% in the formulation series and 85.72% when compared with diclofenac gel. This supports sustained topical availability, but the inconsistency reduces quantitative comparability.	Skin model assay: ex vivo diffusion/permeation through porcine ear skin using Franz diffusion cells. Cumulative permeation ranged from 45.91% to 64.3% at 12 h; the optimized gel reached 88.13% after 24 h. In carrageenan-induced rat paw edema, the optimized ethosomal gel showed 96.74% inhibition, comparable to diclofenac gel at 95.65%.	The study supports the principle that ethosome–gel hybridization can combine Cur solubilization/permeation with semisolid residence and controlled release. The vesicular phase likely facilitates Cur diffusion across the skin barrier, while the gelatin/Carbopol network moderates release and improves topical handling. The system is interpreted as a permeation-enhancing anti-inflammatory gel rather than as a confirmed dermal-reservoir platform.	Relevant as a recent Cur ethosomal gel with porcine skin permeation and topical anti-inflammatory evidence. Limitations include no PDI, no flux/Jss/Kp, no skin retention or layer-specific deposition, no molecular inflammatory biomarkers, short and inconsistently reported stability. The carrageenan model supports acute anti-inflammatory activity but does not represent chronic dermal diseases such as psoriasis, eczema, or acne.	[213]
12	No conventional in vitro release assay was performed. The relevant performance indicator was skin-layer deposition rather than receptor-phase release. Cur was undetectable in the receptor compartment after 24 h, suggesting that the formulation favored local skin retention over systemic transdermal passage. Therefore, the system should be interpreted as a localized dermal delivery platform, not as a systemic transdermal formulation.	Skin model assay: ex vivo full-thickness human skin from cosmetic surgery, Franz diffusion cells, tape stripping of SC and separation of viable epidermis/dermis. Cur deposition increased by 93.4% in SC, 116.4% in epidermis and 86.5% in dermis compared with Cur solution. Cur was not detected in receptor fluid, indicating minimal systemic permeation. ATR-FTIR showed blue shifts in C–H stretching bands, consistent with disruption/fluidization of SC lipid ordering. Antibacterial activity against *S. aureus* was preserved: MIC/MBC 50/100 µg/mL, same as free Cur. HSF cytotoxicity was reduced: IC_50_ 12.76 µg/mL for Cur-transethosomes versus 6.2 µg/mL for free Cur. Scratch assay showed complete wound closure at 72 h with Cur-transethosomes at 5 µg/mL, while control required 96 h; wound closure was accelerated by ~25%.	The improved wound-related performance appears to arise from localized skin deposition rather than systemic permeation. Ethanol disrupted SC lipid packing, Tween 80 increased membrane deformability and steric stabilization, and the nanosized vesicles facilitated intercellular penetration. Vesicular encapsulation also widened the therapeutic window: Cur antimicrobial activity was retained, but fibroblast toxicity decreased, allowing effective wound closure at a lower, safer concentration than free Cur.	Strong dermal relevance because the study uses human skin, layer-specific Cur deposition, ATR-FTIR barrier analysis, antimicrobial testing, cytotoxicity and scratch wound healing. However, it lacks no in vivo wound model, no conventional release kinetics, no flux/Jss/Kp because Cur was not detected in receptor fluid, no room-temperature/accelerated stability, no chemical stability of Cur over time, and no evaluation in infected or chronic wound models. Best interpreted as a promising localized transethosomal Cur platform for wound healing, pending formulation into a clinically usable topical vehicle.	[214]
13	Both systems showed biphasic Cur release, with an initial faster phase followed by slower diffusion-controlled release. Ethosomes released Cur faster than transferosomes, consistent with ethanol-induced membrane fluidization and higher bilayer permeability. Transfersomes released Cur more slowly, suggesting stronger retention within surfactant-modified lipid bilayers. Release followed Higuchi kinetics and Korsmeyer–Peppas, with Fickian diffusion.	Cytotoxicity was evaluated in HUT-78 cutaneous T-cell lymphoma cells after 72 h exposure. IC_50_ decreased from 0.031 mg/mL for free Cur to 0.025 mg/mL for transferosomal Cur and 0.020 mg/mL for ethosomal Cur. Modulation index: 1.24 for transfersomes and 1.55 for ethosomes. Empty vesicles showed low intrinsic toxicity, with <25% viability reduction at equivalent lipid concentrations.	The comparative data suggests that vesicular encapsulation improved Cur antiproliferative activity mainly by improving drug dispersion, apparent availability, and possibly cellular interaction. Ethosomes were more active than transfersomes, likely because ethanol increased membrane permeability and because selected ethosomes were smaller, less polydisperse, and more negatively charged than selected transfersomes. However, this remains a cellular-performance inference, not a demonstrated dermal-delivery mechanism.	Useful as a formulation-performance and skin-oncology biological precedent for Cur-loaded ethosomes/transferosomes. However, its relevance to dermal delivery is indirect because no skin permeation or retention experiment was performed. Limitations include large vesicle size, especially for transfersomes, no semisolid topical dosage form, no skin barrier model, short 1-month stability, no normal skin-cell safety comparison, and no mechanistic uptake assay. Best interpreted as a comparative formulation study showing how ethanol, phospholipid concentration, and edge activator type modulate Cur loading, release, and cytotoxic performance.	[57]
Chlorogenic acid (CGA)					
14	Sustained release/permeation was linked to the lipid–surfactant bilayer plus Carbopol gel. Transferosomes produced higher cumulative permeability, ≥85%, than protransferosomes, ~80%, and CGA hydrogel. Flux enhancement was higher for transferosomes, 1.35 ± 0.03, than protransferosomes, 1.06 ± 0.08.	Skin model assay: Ex vivo rat-skin permeation. CLSM, antioxidant assays, antibacterial assays and excision wound model. Transferosomes gave higher wound contraction on day 9, 99.43%, than protransferosomes, 98.45%, and CGA hydrogel, 88.89%. Histology showed thicker collagen fibers, more fibroblasts and complete epithelial layer with vesicular hydrogels.	The main phenomenon is not particle size alone, but surfactant-driven deformability. Tween 80 appears to intercalate more efficiently with Lipoid S100, generating deformable vesicles that compensate for larger size and higher PDI. This improves flux and wound closure. Span 60 improves structural compactness and charge stabilization but may restrict deformability.	Strong CGA dermal evidence. However, PDI values are high for both systems, especially transferosomes. The study is useful for wound healing but still lacks human/porcine skin, detailed layer-specific CGA quantification and molecular wound biomarkers.	[215]
15	In vitro diffusion was high for both systems: Niosomes 97.96 ± 1.67% and proniosomes 91.00 ± 1.32%. Permeability coefficient was higher for Niosomes, 67 × 10^−3^ ± 1.72, than proniosomes, 52 × 10^−3^ ± 1.09.	Skin model assay: ex vivo permeability, DPPH/Fe^2+^ chelation, antibacterial assays and rat excision wound model. Antioxidant capacity followed proniosomes > niosomes. MIC values were 8× and 16× lower than plain CGA. Wound contraction on day 9 was proniosomes 99.56% > niosomes 98.44% > marketed gel 92.89% > CGA hydrogel 88.89%.	This study shows a relevant trade-off: niosomes improved permeation through higher deformability, whereas proniosomes improved monodispersity, stability and biological performance. For CGA, which is hydrophilic and permeability-limited, the optimal dermal effect depends on both deformable transport and local antioxidant/antibacterial retention.	Strong topical CGA evidence. Main limitations: high PDI for niosomes, rat skin only, UV–Vis quantification, no human/porcine skin, no molecular wound-healing markers, and limited mechanistic separation between permeation, antimicrobial effect and antioxidant contribution.	[56]
16	Free CGA released rapidly, 78.82% at 0.5 h and 98.70% at 8 h. Modified liposome slowed release, 33.22% at 0.5 h and 62.20% at 24 h, consistent with systemic sustained exposure rather than topical retention.	RAW264.7 uptake increased with SA modification and was reduced by free-SA competition, supporting receptor-mediated macrophage uptake. In B16F10 melanoma mice, tumor inhibition was 10.42% for liposome modified, 28.01% for anti-PD-1 and 50.16% for the combination. Combination therapy increased M1/M2 TAM ratio, increased CD8^+^ T cells and reduced CD4^+^Foxp3^+^ T cells.	The mechanistic value is immunological: modified liposomes redirected CGA toward TAM modulation, improving checkpoint blockade. The system is not acting primarily through direct melanoma cytotoxicity but through TME reprogramming.	Useful for the dermato-oncology discussion, but not for dermal delivery. No topical route, no skin permeation, no tumor-skin localization, no semisolid formulation and no direct comparison with dermal systems.	[216]

Abbreviations: Cur: curcumin; CGA: chlorogenic acid; DPPH: 2,2-diphenyl-1-picrylhydrazyl; *E. coli*: *Escherichia coli*; *S. aureus*: *Staphylococcus aureus*; WST-1: water-soluble tetrazolium salt-1; CAM: chorioallantoic membrane; PAM: polyacrylamide; TEM: transmission electron microscopy; SEM: scanning electron microscopy; Ibr: ibrutinib; BALB/c: BALB/c laboratory mouse strain; J: permeation flux; IC_50_: half-maximal inhibitory concentration; HaCaT: human immortalized keratinocyte cell line; IMQ: imiquimod; PASI: Psoriasis Area and Severity Index; IBC: ibrutinib–curcumin combination/liposomal gel; IL-22: interleukin-22; TNF-α: tumor necrosis factor alpha; IL-17: interleukin-17; IL-23: interleukin-23; IL-6: interleukin-6; IL-2: interleukin-2; PDI: polydispersity index; EE: encapsulation efficiency; EL: egg lecithin; Chol: cholesterol; CDLs: cationic deformable liposomes; ADLs: anionic deformable liposomes; NDLs: neutral deformable liposomes; LPS: lipopolysaccharide; RAW 264.7: murine macrophage cell line; NO: nitric oxide; HFF: human foreskin fibroblasts; *S. pyogenes*: *Streptococcus pyogenes*; SDC: sodium deoxycholate; pH: potential of hydrogen; H&E: hematoxylin and eosin staining; HSF: human skin fibroblasts; PBS: phosphate-buffered saline; Cur-Bet-IL: curcumin–betaine ionic liquid; Cur-Bet-IL-Lip: curcumin–betaine ionic liquid liposome; SL: soy lecithin; PO: Plurol Oleique CC-497; SC: stratum corneum; CCD: central composite design; C_max_: maximum plasma concentration; T_max_: time to maximum plasma concentration; t1/2: elimination half-life; AUC_0–t_: area under the concentration–time curve from time zero to the last measured time point; AUC_0–∞_: area under the concentration–time curve from time zero to infinity; MRT_0–∞_: mean residence time from time zero to infinity; MIC: minimum inhibitory concentration; MBC: minimum bactericidal concentration; *P. acnes*: *Propionibacterium acnes*; *S. epidermidis*: *Staphylococcus epidermidis*; HN: hyaluronan-containing niosomes; Quer: quercetin; HA: hyaluronic acid/hyaluronan; PG: propylene glycol; PC: phosphatidylcholine; HPC: hydrogenated phosphatidylcholine; ER: enhancement ratio; GA: glycyrrhetinic acid; TPGS: D-α-tocopheryl polyethylene glycol succinate; PEG: polyethylene glycol; CLSM: confocal laser scanning microscopy; p-IκBα: phosphorylated inhibitor of κB alpha; p-p65: phosphorylated p65 NF-κB subunit; p-STAT3: phosphorylated signal transducer and activator of transcription 3; ROS: reactive oxygen species; NF-κB: nuclear factor kappa-light-chain-enhancer of activated B cells; ROR-γt: retinoic acid receptor-related orphan receptor gamma t; GR-α: glucocorticoid receptor alpha; SOD: superoxide dismutase; CAT: catalase; Jss: steady-state flux; Kp: permeability coefficient; ATR-FTIR: attenuated total reflectance Fourier transform infrared spectroscopy; HUT-78: human cutaneous T-cell lymphoma cell line; Fe^2+^: ferrous ion; UV–Vis: ultraviolet–visible spectroscopy; SA: sialic acid; B16F10: murine melanoma cell line; anti-PD-1: anti-programmed cell death protein 1 antibody; M1/M2: classically activated/pro-inflammatory and alternatively activated/anti-inflammatory macrophage phenotypes; TAM: tumor-associated macrophage; CD8^+^ T cells: cluster of differentiation 8-positive T cells; CD4^+^Foxp3^+^ T cells: cluster of differentiation 4-positive forkhead box P3-positive T cells; TME: tumor microenvironment.

## Data Availability

No new data were created or analyzed in this study. Data sharing is not applicable to this article.

## References

[1] Aguiar B., Carmo H., Garrido J., Sousa Lobo J.M., Almeida I.F. (2022). In Vitro Evaluation of the Photoreactivity and Phototoxicity of Natural Polyphenol Antioxidants. Molecules.

[2] Ferreira M.S., Magalhães M.C., Oliveira R., Sousa-Lobo J.M., Almeida I.F. (2021). Trends in the Use of Botanicals in Anti-Aging Cosmetics. Molecules.

[3] Eseberri I., Trepiana J., Léniz A., Gómez-García I., Carr-Ugarte H., González M., Portillo M.P. (2022). Variability in the Beneficial Effects of Phenolic Compounds: A Review. Nutrients.

[4] Aghababaei F., Hadidi M. (2023). Recent Advances in Potential Health Benefits of Quercetin. Pharmaceuticals.

[5] Ferrari E., Naponelli V. (2025). Catechins and Human Health: Breakthroughs from Clinical Trials. Molecules.

[6] Prokop A., Magiera A., Olszewska M.A. (2025). Proanthocyanidins as Therapeutic Agents in Inflammation-Related Skin Disorders. Int. J. Mol. Sci..

[7] Roux J., Horton L., Babadjouni A., Kincaid C.M., Mesinkovska N.A. (2025). Ferulic Acid Use for Skin Applications: A Systematic Review. J. Clin. Aesthet. Dermatol..

[8] Magnani C., Isaac V.L.B., Correa M.A., Salgado H.R.N. (2014). Caffeic acid: A review of its potential use in medications and cosmetics. Anal. Methods.

[9] Nguyen V., Taine E.G., Meng D., Cui T., Tan W. (2024). Chlorogenic Acid: A Systematic Review on the Biological Functions, Mechanistic Actions, and Therapeutic Potentials. Nutrients.

[10] Salehi B., Mishra A.P., Nigam M., Sener B., Kilic M., Sharifi-Rad M., Fokou P.V.T., Martins N., Sharifi-Rad J. (2018). Resveratrol: A Double-Edged Sword in Health Benefits. Biomedicines.

[11] Rom S., Zuluaga-Ramirez V., Reichenbach N.L., Erickson M.A., Winfield M., Gajghate S., Christofidou-Solomidou M., Jordan-Sciutto K.L., Persidsky Y. (2018). Secoisolariciresinol diglucoside is a blood-brain barrier protective and anti-inflammatory agent: Implications for neuroinflammation. J. Neuroinflamm..

[12] Mali A.V., Padhye S.B., Anant S., Hegde M.V., Kadam S.S. (2019). Anticancer and antimetastatic potential of enterolactone: Clinical, preclinical and mechanistic perspectives. Eur. J. Pharmacol..

[13] Ding X., Zhang G., Yiu C.K.Y., Li X., Shan Z. (2025). Unleashing the Potential of Tannic Acid in Dentistry: A Scoping Review of Applications. Bioengineering.

[14] Zhang Y., Li M., Liu H., Fan Y., Liu H.H. (2025). The application of procyanidins in diabetes and its complications: A review of preclinical studies. Front. Pharmacol..

[15] El-Saadony M.T., Saad A.M., Mohammed D.M., Alkafaas S.S., Ghosh S., Negm S.H., Salem H.M., Fahmy M.A., Mosa W.F.A., Ibrahim E.H. (2025). Curcumin, an active component of turmeric: Biological activities, nutritional aspects, immunological, bioavailability, and human health benefits—A comprehensive review. Front. Immunol..

[16] Ciupei D., Colişar A., Leopold L., Stănilă A., Diaconeasa Z.M. (2024). Polyphenols: From Classification to Therapeutic Potential and Bioavailability. Foods.

[17] Parisi O.I., Puoci F., Restuccia D., Farina G., Iemma F., Picci N., Watson R.R., Preedy V.R., Zibadi S. (2014). Chapter 4—Polyphenols and Their Formulations: Different Strategies to Overcome the Drawbacks Associated with Their Poor Stability and Bioavailability. Polyphenols in Human Health and Disease.

[18] Anunciato Casarini T.P., Frank L.A., Pohlmann A.R., Guterres S.S. (2020). Dermatological applications of the flavonoid phloretin. Eur. J. Pharmacol..

[19] Mamta, Prakash G., Singhal A.B., Albishri N., Gupta B. (2026). Psychological drivers of customer citizenship buying behaviour in green cosmetics: Evidence from a mixed-methods approach. Acta Psychol..

[20] Koay K.Y., Ahmed H.I. (2025). Understanding consumers’ purchase intentions for green cosmetic products: An integrated perspective. Soc. Responsib. J..

[21] Suphasomboon T., Vassanadumrongdee S. (2022). Toward sustainable consumption of green cosmetics and personal care products: The role of perceived value and ethical concern. Sustain. Prod. Consum..

[22] Mamta, Prakash G., Agarwal R., Alghafes R., Sharma V.K. (2025). Navigating consumer intentions and actual transitions to green cosmetics in emerging retail markets. Int. J. Retail. Distrib. Manag..

[23] Górnicka J., Mika M., Wróblewska O., Siudem P., Paradowska K. (2023). Methods to Improve the Solubility of Curcumin from *Turmeric*. Life.

[24] Dhingra D., Bisht M., Bhawna B., Pandey S. (2021). Enhanced solubility and improved stability of curcumin in novel water-in-deep eutectic solvent microemulsions. J. Mol. Liq..

[25] Yuan Q., Liu C., Zhang Z., Chen F., Xiao Q., Zhang L., Pan X., He F., Xiao M. (2025). Pharmacological advances of the chlorogenic acids family: Current insights and future research directions. Front. Pharmacol..

[26] Truzzi F., Tibaldi C., Zhang Y., Dinelli G., D’Amen E. (2021). An Overview on Dietary Polyphenols and Their Biopharmaceutical Classification System (BCS). Int. J. Mol. Sci..

[27] Paredes-Toledo J., Herrera J., Morales J., Robert P., Oyarzun-Ampuero F., Giménez B. (2024). Bioaccessibility of chlorogenic acid and curcumin co-encapsulated in double emulsions with the inner interface stabilized by functionalized silica nanoparticles. Food Chem..

[28] Ren J., Fan Y., Xiao X., Cao Y., Zou Y., Luo X., Liu F. (2026). Construction of a gelatinized starch-based chlorogenic acid emulsion: Transcriptomic insights into antibacterial mechanism and application in cod preservation. Food Hydrocoll..

[29] Guo Q., Qi X., Chen X., Yang Y., Wang S. (2025). Development of a stable curcumin-loaded emulsion with desired properties under a low oil phase proportion using starch-palmitic acid-β-lactoglobulin complexes. Int. J. Biol. Macromol..

[30] Tan L., Sha K., Jia X., Yin L., Li L., Liu H. (2026). Regulating the structure of soybean protein isolate-dextran maillard conjugate through cold plasma treatment: Enhancing the storage and processing stability of oil-in-water emulsion loaded with curcumin. Food Res. Int..

[31] Jana S., Hicks W., Garbus-Grant H., Goel Y., Prince R., Friedman J., Gupta K., Alayash A.I. (2026). A novel transdermal curcumin gel shows potential in improving cardiac bioenergetic functions in Berkeley sickle cell mice. Blood Vessels Thromb. Hemost..

[32] Duan J., Chen M., Wang M., He R., Li K., Shi L. (2025). Hierarchically assembled curcumin-vitamin E-zinc thermo-gel for multi-synergistic prevention and treatment of radiation-induced oral mucositis. Mater. Today Bio.

[33] Luo Y., Cao J., Wang J., Zhang Y., Teng W., Wang Y. (2025). The influence of sodium tripolyphosphate, chlorogenic acid and sodium alginate on the structure and properties of fibrin gel. Int. J. Biol. Macromol..

[34] Niu L., Ren X., Guan H., He X., Lin Q., Zhao W., Liang W., Li W. (2025). Complexation of chitosan-modified porous starch with chlorogenic acid to form ternary gels: Multiscale structural changes and retrogradation behavior. Food Chem..

[35] Keramat M., Golmakani M.-T. (2024). Antioxidant potency and inhibitory mechanism of curcumin and its derivatives in oleogel and emulgel produced by linseed oil. Food Chem..

[36] Das J., Das I.J., Giri Y., Jena R., Swain S.S., Jena K., Chowdary N.B., Samal H.B. (2025). Development and characterization of curcumin-loaded modified emulgels with natural silk sericin: A novel approach for effective wound healing. Next Res..

[37] Yu Q., Shen Y., Xiao F., Zhao Y., Piao S., Li G., Yan M. (2023). Yuhong ointment ameliorates inflammatory responses and wound healing in scalded mice. J. Ethnopharmacol..

[38] Chaiwangrach N., Waranuch N., Temkitthawon P., Wongwad E., Nuengchamnong N., Usuwanthim K., Saesong T., Rakkhetkorn Y., Pisutthanan S., Ingkaninan K. (2024). Quality assessment, stability and in-vitro anti-inflammatory activities of a topical ointment containing cannabis and turmeric extracts for haemorrhoids and skin diseases. Phytomed. Plus.

[39] Cai M., Li W., Sun G. (2025). A multidimensional quality control strategy for traditional Chinese medicine lotions: HPLC, B-Z oscillation fingerprinting, and in vitro antioxidant activity of Jinsong antipruritic lotion. Microchem. J..

[40] Suhaimi H., Ahmad F.B.H., Friberg S.E. (1995). Curcumin in a Model Skin Lotion Formulation. J. Pharm. Sci..

[41] Yang Y., Wu S., Li K., Wang X., Huo H., Zhang P., Wang Q., Lin Z., Cao L. (2026). UV curable antibacterial chitosan/chlorogenic acid/polyacrylamide hydrogel packaging film. Colloids Surf. A Physicochem. Eng. Asp..

[42] Thach U.D., Tran N.C.T., Phan H.L., Le Q.-V., Do B.H., Atayde E.C., Wu K.C.W., Jamnongkan T. (2026). Injectable PEG-based poly(β-amino ester) hydrogels with tunable biodegradability for stimuli-responsive curcumin delivery. J. Taiwan. Inst. Chem. Eng..

[43] Lens M. (2025). Phospholipid-Based Vesicular Systems as Carriers for the Delivery of Active Cosmeceutical Ingredients. Int. J. Mol. Sci..

[44] Kumar B., Sahoo P.K., Manchanda S. (2022). Formulation, characterization and ex vivo study of curcumin nano-invasomal gel for enhanced transdermal delivery. OpenNano.

[45] Arora D., Nanda S. (2019). Quality by design driven development of resveratrol loaded ethosomal hydrogel for improved dermatological benefits via enhanced skin permeation and retention. Int. J. Pharm..

[46] Trivedi H.R., Puranik P.K. (2023). Chlorogenic acid-optimized nanophytovesicles: A novel approach for enhanced permeability and oral bioavailability. Future J. Pharm. Sci..

[47] Antonara L., Triantafyllopoulou E., Chountoulesi M., Pippa N., Dallas P.P., Rekkas D.M. (2025). Lipid-Based Drug Delivery Systems: Concepts and Recent Advances in Transdermal Applications. Nanomaterials.

[48] Sun Q. (2022). The Hydrophobic Effects: Our Current Understanding. Molecules.

[49] Israelachvili J.N., Mitchell D.J., Ninham B.W. (1977). Theory of self-assembly of lipid bilayers and vesicles. Biochim. Biophys. Acta (BBA) Biomembr..

[50] Aye K.C., Pengnam S., Pamornpathomkul B., Charoenying T., Patrojanasophon P., Opanasopit P., Pornpitchanarong C. (2026). A dual-strategy nanocomposite hydrogel platform of nanosuspensions and deformable liposomes for enhanced curcumin delivery against skin cancer cells. Eur. J. Pharm. Sci..

[51] Li Q.-Q., Yan J.-H., Zhou Z.-E., Geng X., Xiong J.-H. (2024). Enhanced anti-inflammatory activity of chlorogenic acid via folic acid-TPGS-modified liposomes encapsulation: Characterization and In vivo evaluation on colitis mice. Front. Pharmacol..

[52] Righeschi C., Bergonzi M.C., Isacchi B., Bazzicalupi C., Gratteri P., Bilia A.R. (2016). Enhanced curcumin permeability by SLN formulation: The PAMPA approach. LWT-Food Sci. Technol..

[53] Yugatama A., Chao F.-C., Hsu M.-J., Huang S.-W., Liou J.-P., Hsieh C.-M. (2026). Design and development of dual drug-loaded liposomes encapsulating gentamicin and curcumin via microfluidic synthesis: A Box-Behnken experimental approach. J. Drug Deliv. Sci. Technol..

[54] Sailaja A.K., Meghana T. (2024). Formulation and Evaluation of Invasomal and Ethosomal Gel for Curcumin and Determination of Anti-Fungal Activity. Curr. Nanomater..

[55] Aboali F.A., Habib D.A., Elbedaiwy H.M., Farid R.M. (2020). Curcumin-loaded proniosomal gel as a biofreindly alternative for treatment of ocular inflammation: In-vitro and in-vivo assessment. Int. J. Pharm..

[56] Trivedi H.R., Puranik P.K. (2023). Chlorogenic acid loaded niosomes and proniosomes: In vitro antioxidant and antibacterial activities with efficacy in wound healing. Digit. Chin. Med..

[57] Momekova D., Gugleva V., Petrov P. (2024). Development and evaluation of curcumin-loaded vesicular carriers: Impact of formulation variables. Pharmacia.

[58] Zhang J.-J., Luo Q.-S., Li Q.-Q., Xu Q., Geng X., Xiong J.-H. (2024). Fabrication and characterization of TPGS-modified chlorogenic acid liposomes and its bioavailability in rats. RSC Adv..

[59] Gulcin İ. (2025). Antioxidants: A comprehensive review. Arch. Toxicol..

[60] Zhang P., Li M., Li X., Lu X., Wang W., Sun J., Ma Q., Zhu Y. (2026). Curcumin-mediated photodynamic inactivation for smart food preservation: Antimicrobial mechanisms, nano-enabling strategies, and application challenges. Trends Food Sci. Technol..

[61] Huang W.-j., Dai W.-h., Amonsou E.O., Yao G.-q., Liu Y.-y., Zhang J.-h., Jin W.-p., Shen W.-y., Hu Z.-z., Wang Z. (2026). Characterization of electrospinned halloysite-curcumin active nanofiber films and its application in food preservation. LWT.

[62] Tavassoli M., Aslam R., Bahramian B., Abedi-Firoozjah R., Maqsood S. (2026). Chlorogenic acid as a novel bioactive and functional ingredient in the fabrication of biodegradable food packaging films. Trends Food Sci. Technol..

[63] Ilea M.I.M., Guillén F., Zapata P.J., Fernández-Picazo C., Dobón-Suárez A., Díaz-Mula H.M. (2026). Chlorogenic acid as an innovative strategy to delay postharvest senescence and maintain quality in strawberries during cold storage. Appl. Food Res..

[64] Somaida A., Abdelsalam A.M., Mariey S., Mohamed M.S., Amin M.A., Zayed G.M., Al-Sawahli M.M. (2026). Micellar zein versus PEGylated liposome encapsulating curcumin: A comparative study towards Human U-87 glioblastoma spheroids. J. Drug Deliv. Sci. Technol..

[65] Zhang C., Sui Y., Liu S., Yang M. (2025). Pharmaceutical targets of curcumin in liver cancer treatment and molecular mechanisms: A bioinformatics analysis and computer-aided study. Silico Res. Biomed..

[66] Ma D., Xu Y., Liang R., Meng Q., Liu Y., Hu D., Zhu B., Zheng Y., Luo Q. (2026). Chlorogenic acid modulates gut microbiota and metabolites to alleviate intrahepatic cholestasis of pregnancy: Insights from 16S rRNA sequencing and metabolomics. Biochem. Biophys. Rep..

[67] Zhang Q., Wang M., Deng S., Sidike G., Zhao Z., Zhang Q., Liu Y., Qin W. (2026). Whey protein/chlorogenic acid/high methoxy pectin water-in-oil-in-water emulsion alleviates DSS-induced ulcerative colitis in mice by regulating gut microbiota and LPS/TLR4/MyD88/NF-κB pathways. J. Future Foods.

[68] Yao H., Shi S., Zhao R. (2026). Protective Effect of Chlorogenic Acid Complex against Intestinal Oxidative Stress in Broilers and Screening of Its Key Isomer. Poult. Sci..

[69] Ma Z.-X., Yin Z.-Q., Feng Z.-A., Xie X.-P., Li A., Fu S.-H., Yue Q., Wang S., Ma L.-K., Duan Y.-J. (2026). Food-derived chlorogenic acid prevents aortic aneurysm and dissection by nutritional restore branched-chain amino acid dyshomeostasis. J. Nutr. Biochem..

[70] Chen J., Xie K., Yu B., Luo Y., Yan H., Zheng P., Mao X., Yu J., Wang Q., He J. (2026). Dietary supplementation of curcumin improves growth performance, carcass traits and meat quality in growing-finishing pigs. J. Funct. Foods.

[71] Asghari A., Gharekhani M., Asefi N., Ostadrahimi A. (2026). Utilization of chia–canola oleogels with curcumin for fat replacement in gluten-free cakes: A strategy for healthier bakery products. LWT.

[72] Arct J., Ratz-Łyko A., Mieloch M., Witulska M. (2014). Evaluation of skin colouring properties of curcuma longa extract. Indian. J. Pharm. Sci..

[73] Bikiaris N.D., Kapourani A., Pantazos I., Barmpalexis P. (2025). Enhanced Bioavailability and Stability of Curcumin in Cosmeceuticals: Exploiting Droplet Microfluidics for Nanoemulsion Development. Cosmetics.

[74] Rodrigues R., Oliveira M.B.P.P., Alves R.C. (2023). Chlorogenic Acids and Caffeine from Coffee By-Products: A Review on Skincare Applications. Cosmetics.

[75] Girsang E., Ginting C.N., Lister I.N.E., Gunawan K.Y., Widowati W. (2021). Anti-inflammatory and antiaging properties of chlorogenic acid on UV-induced fibroblast cell. PeerJ.

[76] Barzegar A., Moosavi-Movahedi A.A. (2011). Intracellular ROS protection efficiency and free radical-scavenging activity of curcumin. PLoS ONE.

[77] Kavi Rajan R., Hussein M.Z., Fakurazi S., Yusoff K., Masarudin M.J. (2019). Increased ROS Scavenging and Antioxidant Efficiency of Chlorogenic Acid Compound Delivered via a Chitosan Nanoparticulate System for Efficient In Vitro Visualization and Accumulation in Human Renal Adenocarcinoma Cells. Int. J. Mol. Sci..

[78] Wang X., Fan X., Yuan S., Jiao W., Liu B., Cao J., Jiang W. (2017). Chlorogenic acid protects against aluminium-induced cytotoxicity through chelation and antioxidant actions in primary hippocampal neuronal cells. Food Funct..

[79] Mary C.P.V., Vijayakumar S., Shankar R. (2018). Metal chelating ability and antioxidant properties of Curcumin-metal complexes—A DFT approach. J. Mol. Graph. Model..

[80] Cheng M., Ding F., Li L., Dai C., Sun X., Xu J., Chen F., Li M., Li X. (2025). Exploring the role of curcumin in mitigating oxidative stress to alleviate lipid metabolism disorders. Front. Pharmacol..

[81] Cheng D., Wang G., Wang X., Tang J., Li C. (2020). Chlorogenic acid improves lipid membrane peroxidation and morphological changes in nitrite-induced erythrocyte model of methemoglobinemia. J. Food Biochem..

[82] Lin J., Saeed U., Piracha Z.Z., Ma Z. (2026). Curcumin modulates NF-κB and Nrf2 pathways to mitigate inflammation, oxidative stress, and virulence in Pseudomonas aeruginosa-infected bronchial epithelial cells. Arch. Microbiol..

[83] Siswanto F.M., Sakuma R., Oguro A., Imaoka S. (2022). Chlorogenic Acid Activates Nrf2/SKN-1 and Prolongs the Lifespan of Caenorhabditis elegans via the Akt-FOXO_3_/DAF16a-DDB1 Pathway and Activation of DAF16f. J. Gerontol. A Biol. Sci. Med. Sci..

[84] Sharifi-Rad J., Rayess Y.E., Rizk A.A., Sadaka C., Zgheib R., Zam W., Sestito S., Rapposelli S., Neffe-Skocińska K., Zielińska D. (2020). *Turmeric* and Its Major Compound Curcumin on Health: Bioactive Effects and Safety Profiles for Food, Pharmaceutical, Biotechnological and Medicinal Applications. Front. Pharmacol..

[85] Kaur H., Kaur G. (2014). A Critical Appraisal of Solubility Enhancement Techniques of Polyphenols. J. Pharm..

[86] Kotha R.R., Luthria D.L. (2019). Curcumin: Biological, Pharmaceutical, Nutraceutical, and Analytical Aspects. Molecules.

[87] Hu A., Lin S.-Y., Cheng S.-C., Shih T.-L. (2026). Determine of pH-dependent diketo and keto-enol tautomers of curcumin analogs by ultraperformance liquid chromatography-mass spectrometry. Microchem. J..

[88] Ciuca M.D., Racovita R.C. (2023). Curcumin: Overview of Extraction Methods, Health Benefits, and Encapsulation and Delivery Using Microemulsions and Nanoemulsions. Int. J. Mol. Sci..

[89] Manasa P.S.L., Kamble A.D., Chilakamarthi U. (2023). Various Extraction Techniques of Curcumin-A Comprehensive Review. ACS Omega.

[90] Slaček G., Kotnik P., Osmić A., Postružnik V., Knez Ž., Finšgar M., Knez Marevci M. (2023). The Extraction Process, Separation, and Identification of Curcuminoids from *Turmeric Curcuma longa*. Foods.

[91] Nisoa M., Kaewpradit S., Nahar L., Sarker S.D., Charoensup R., Puttarak P., Yusakul G. (2025). Extraction of curcumin and curcuminoids: From conventional methods to innovative extraction using deep eutectic solvents. Microchem. J..

[92] Shekaari H., Zafarani-Moattar M.T., Mokhtarpour M. (2022). Effective ultrasonic-assisted extraction and solubilization of curcuminoids from turmeric by using natural deep eutectic solvents and imidazolium-based ionic liquids. J. Mol. Liq..

[93] Sahlan M., Rosarina D., Ratna Suminar H.F., Diatama Pohan Y., Hidayatullah I.M., Narawangsa D.R., Putri D.N., Sari E., Perdani M.S., Wibowo Y.G. (2025). Microwave–Ultrasound-Assisted Extraction Coupled with Natural Deep Eutectic Solvent Enables High-Yield, Low-Solvent Recovery of Curcumin from *Curcuma longa* L. Pharmaceutics.

[94] Kurmudle N., Kagliwal L.D., Bankar S.B., Singhal R.S. (2013). Enzyme-assisted extraction for enhanced yields of turmeric oleoresin and its constituents. Food Biosci..

[95] Schieffer G.W. (2002). Pressurized liquid extraction of curcuminoids and curcuminoid degradation products from turmeric (*Curcuma longa*) with subsequent HPLC assays. J. Liq. Chromatogr. Relat. Technol..

[96] Widmann A.K., Wahl M.A., Kammerer D.R., Daniels R. (2022). Supercritical Fluid Extraction with CO_2_ of *Curcuma longa* L. in Comparison to Conventional Solvent Extraction. Pharmaceutics.

[97] Peng Y., Ao M., Dong B., Jiang Y., Yu L., Chen Z., Hu C., Xu R. (2021). Anti-Inflammatory Effects of Curcumin in the Inflammatory Diseases: Status, Limitations and Countermeasures. Drug Des. Devel Ther..

[98] Sadeghi M., Dehnavi S., Asadirad A., Xu S., Majeed M., Jamialahmadi T., Johnston T.P., Sahebkar A. (2023). Curcumin and chemokines: Mechanism of action and therapeutic potential in inflammatory diseases. Inflammopharmacology.

[99] Dehzad M.J., Ghalandari H., Nouri M., Askarpour M. (2023). Antioxidant and anti-inflammatory effects of curcumin/turmeric supplementation in adults: A GRADE-assessed systematic review and dose–response meta-analysis of randomized controlled trials. Cytokine.

[100] Mokgalaboni K., Mashaba R.G., Phoswa W.N., Lebelo S.L. (2024). Curcumin Attenuates Hyperglycemia and Inflammation in Type 2 Diabetes Mellitus: Quantitative Analysis of Randomized Controlled Trial. Nutrients.

[101] Zeng L., Yang T., Yang K., Yu G., Li J., Xiang W., Chen H. (2022). Efficacy and Safety of Curcumin and *Curcuma longa* Extract in the Treatment of Arthritis: A Systematic Review and Meta-Analysis of Randomized Controlled Trial. Front. Immunol..

[102] D’andurain J., López V., Arazo-Rusindo M., Tiscornia C., Aicardi V., Simón L., Mariotti-Celis M.S. (2023). Effect of Curcumin Consumption on Inflammation and Oxidative Stress in Patients on Hemodialysis: A Literature Review. Nutrients.

[103] Cox F.F., Misiou A., Vierkant A., Ale-Agha N., Grandoch M., Haendeler J., Altschmied J. (2022). Protective Effects of Curcumin in Cardiovascular Diseases—Impact on Oxidative Stress and Mitochondria. Cells.

[104] Hu P., Li K., Peng X.-X., Kan Y., Yao T.-J., Wang Z.-Y., Li Z., Liu H.-Y., Cai D. (2023). Curcumin derived from medicinal homologous foods: Its main signals in immunoregulation of oxidative stress, inflammation, and apoptosis. Front. Immunol..

[105] Xie Q., Zheng X., Li L., Ma L., Zhao Q., Chang S., You L. (2021). Effect of Curcumin Addition on the Properties of Biodegradable Pectin/Chitosan Films. Molecules.

[106] Vadivel M., Sankarganesh M., Raja J.D., Rajesh J., Mohanasundaram D., Alagar M. (2019). Bioactive constituents and bio-waste derived chitosan/xylan based biodegradable hybrid nanocomposite for sensitive detection of fish freshness. Food Packag. Shelf Life.

[107] Khazi M.I., Liaqat F., Liu X., Yan Y., Zhu D. (2024). Fermentation, functional analysis, and biological activities of turmeric kombucha. J. Sci. Food Agric..

[108] Amchova P., Kotolova H., Ruda-Kucerova J. (2015). Health safety issues of synthetic food colorants. Regul. Toxicol. Pharmacol..

[109] Scartezzini P., Speroni E. (2000). Review on some plants of Indian traditional medicine with antioxidant activity. J. Ethnopharmacol..

[110] Gul P., Bakht J. (2015). Antimicrobial activity of turmeric extract and its potential use in food industry. J. Food Sci. Technol..

[111] Kandaswamy K., Prasad Panda S., Subramanian R., Khan H., Rafi Shaik M., Althaf Hussain S., Guru A., Arockiaraj J. (2024). Synergistic berberine chloride and Curcumin-Loaded nanofiber therapies against Methicillin-Resistant Staphylococcus aureus Infection: Augmented immune and inflammatory responses in zebrafish wound healing. Int. Immunopharmacol..

[112] Elkhateeb O., Badawy M.E.I., Tohamy H.G., Abou-Ahmed H., El-Kammar M., Elkhenany H. (2023). Curcumin-infused nanostructured lipid carriers: A promising strategy for enhancing skin regeneration and combating microbial infection. BMC Vet. Res..

[113] Oves M., Ansari M.O., Ansari M.S., Memić A. (2023). Graphene@Curcumin-Copper Paintable Coatings for the Prevention of Nosocomial Microbial Infection. Molecules.

[114] Chen T., Wan L., Xiao Y., Wang K., Wu P., Li C., Huang C., Liu X., Xue W., Sun G. (2024). Curcumin/pEGCG-encapsulated nanoparticles enhance spinal cord injury recovery by regulating CD74 to alleviate oxidative stress and inflammation. J. Nanobiotechnology.

[115] Wang L., Pan X., Jiang L., Chu Y., Gao S., Jiang X., Zhang Y., Chen Y., Luo S., Peng C. (2022). The Biological Activity Mechanism of Chlorogenic Acid and Its Applications in Food Industry: A Review. Front. Nutr..

[116] Behne S., Franke H., Schwarz S., Lachenmeier D.W. (2023). Risk Assessment of Chlorogenic and Isochlorogenic Acids in Coffee By-Products. Molecules.

[117] Dima C., Assadpour E., Dima S., Jafari S.M. (2020). Bioavailability and bioaccessibility of food bioactive compounds; overview and assessment by in vitro methods. Compr. Rev. Food Sci. Food Saf..

[118] Rajabi H., Zhang W., Wu D., Bo P., Jung Y.H., Rawdkuen S. (2026). Multifunctional Roles of Chlorogenic Acid in Food Packaging Films: Linking Structural Modulation with Active and Intelligent Performance. Foods.

[119] Chanaj-Kaczmarek J., Paczkowska M., Osmałek T., Kaproń B., Plech T., Szymanowska D., Karaźniewicz-Łada M., Kobus-Cisowska J., Cielecka-Piontek J. (2020). Hydrogel Delivery System Containing Calendulae flos Lyophilized Extract with Chitosan as a Supporting Strategy for Wound Healing Applications. Pharmaceutics.

[120] Harwansh R.K., Bhati H., Deshmukh R., Rahman M.A. (2025). Preparation, Characterization, and In Vitro Evaluation of Chlorogenic Acid Loaded Hydrogel Beads for the Management of Ulcerative Colitis. Assay. Drug Dev. Technol..

[121] Schoellhammer C.M., Blankschtein D., Langer R. (2014). Skin permeabilization for transdermal drug delivery: Recent advances and future prospects. Expert. Opin. Drug Deliv..

[122] Chang T., Guo Y., Qu H., Ding L., Zhao Z., Zhang Y., Chu W., Lu X. (2025). Evaluating degradation performance of chlorogenic acid by FeC activated persulfate with magnetic stirring and mechanical stirring under magnetic field. J. Water Process Eng..

[123] Badmos S., Kuhnert N. (2025). Stability and degradation of chlorogenic acids in green and roasted coffee beans during long-term storage11This contribution id dedicated to Prof. W.A. Schenk on the occasion of his 80th birthday. Food Res. Int..

[124] Maiyah N., Kerdpiboon S., Kerr W.L., Klaypradit W., Smithisukul C., Supapvanich S. (2026). Impact of roasting levels and brewing cycles on bioactive compounds in spent coffee grounds. Food Chem. X.

[125] Liao L., Zhang W., Zhang B., Cai Y., Gao L., Ogutu C., Sun J., Zheng B., Wang L., Li L. (2021). Evaluation of chlorogenic acid accumulation in cultivated and wild apples. J. Food Compos. Anal..

[126] Gill S.K., Lever E., Emery P.W., Whelan K. (2019). Nutrient, fibre, sorbitol and chlorogenic acid content of prunes (Prunus domestica): An updated analysis and comparison of different countries of origin and database values. Int. J. Food Sci. Nutr..

[127] Piccolo V., Maisto M., Schiano E., Iannuzzo F., Keivani N., Manuela Rigano M., Santini A., Novellino E., Carlo Tenore G., Summa V. (2024). Phytochemical investigation and antioxidant properties of unripe tomato cultivars (*Solanum lycopersicum* L.). Food Chem..

[128] Villanueva G., Vilanova S., Plazas M. (2024). Characterization of Browning, Chlorogenic Acid Content, and Polyphenol Oxidase Activity in Different Varietal Types of Eggplant (Solanum melongena) for Improving Visual and Nutritional Quality. Plants.

[129] Jacobo-Velázquez D.A., Guerrero-Analco J.A., Monribot-Villanueva J.L., Bojorquez-Rodríguez E.M. (2025). Storage of shredded carrots induces accumulation of chlorogenic acid and other phenolics without generating toxic or anti-nutritional metabolites: A metabolomics study. LWT.

[130] Yang H., Luo W., Gao D. (2025). Chlorogenic Acid Content and Metabolism-related Gene Regulation of Potato Tuber Flesh Induced by Sucrose and Phytohormones. Potato Res..

[131] Basit A., Shahzad R., Mueed A., Muhammad A., Rong W., Ya L., Xin G., Shahrokh K., Arshad M., Shutian T. (2025). A critical review on pear Fruit’s polyphenols and its chlorogenic acid: Composition, bioavailability, and pharmacological potential. Food Biosci..

[132] Lim D.W., Han T., Jung J., Song Y., Um M.Y., Yoon M., Kim Y.T., Cho S., Kim I.H., Han D. (2018). Chlorogenic Acid from Hawthorn Berry (*Crataegus pinnatifida* Fruit) Prevents Stress Hormone-Induced Depressive Behavior, through Monoamine Oxidase B-Reactive Oxygen Species Signaling in Hippocampal Astrocytes of Mice. Mol. Nutr. Food Res..

[133] Rotondo R., Bürgi M., Masin M., Adalid-Martínez A., Prohens J., Rodríguez G.R., Escalante A.M. (2025). Myricetin and chlorogenic acid from globe artichoke (*Cynara cardunculus* var. *scolymus*) as negative modulators of human Interferon I-α. Heliyon.

[134] Ramadan M., Fadel A., Bahi A., Alajlani S., Al Neyadi M.S.S., Alshamsi E.S.H., Naser S., Shahbaz H.M., Samuel R., Ranneh Y. (2025). Optimizing Chlorogenic Acid Extraction From Spent Coffee Grounds: A Comparative Review of Conventional and Non-Conventional Techniques. Food Sci. Nutr..

[135] Bouhzam I., Cantero R., Margallo M., Aldaco R., Bala A., Fullana-i-Palmer P., Puig R. (2023). Extraction of Bioactive Compounds from Spent Coffee Grounds Using Ethanol and Acetone Aqueous Solutions. Foods.

[136] Shi A., Li T., Zheng Y., Song Y., Wang H., Wang N., Dong L., Shi H. (2021). Chlorogenic Acid Improves NAFLD by Regulating gut Microbiota and GLP-1. Front. Pharmacol..

[137] Zhou X., Zhang B., Zhao X., Lin Y., Zhuang Y., Guo J., Wang S. (2022). Chlorogenic Acid Prevents Hyperuricemia Nephropathy via Regulating TMAO-Related Gut Microbes and Inhibiting the PI3K/AKT/mTOR Pathway. J. Agric. Food Chem..

[138] Zheng Y., Li L., Chen B., Fang Y., Lin W., Zhang T., Feng X., Tao X., Wu Y., Fu X. (2022). Chlorogenic acid exerts neuroprotective effect against hypoxia-ischemia brain injury in neonatal rats by activating Sirt1 to regulate the Nrf2-NF-κB signaling pathway. Cell Commun. Signal..

[139] La Rosa G., Sozio C., Pipicelli L., Raia M., Palmiero A., Santillo M., Damiano S. (2023). Antioxidant, Anti-Inflammatory and Pro-Differentiative Effects of Chlorogenic Acid on M03-13 Human Oligodendrocyte-like Cells. Int. J. Mol. Sci..

[140] Cicek B., Hacimuftuoglu A., Yeni Y., Danisman B., Ozkaraca M., Mokhtare B., Kantarci M., Spanakis M., Nikitovic D., Lazopoulos G. (2023). Chlorogenic Acid Attenuates Doxorubicin-Induced Oxidative Stress and Markers of Apoptosis in Cardiomyocytes via Nrf2/HO-1 and Dityrosine Signaling. J. Pers. Med..

[141] Gu T., Zhang Z., Liu J., Chen L., Tian Y., Xu W., Zeng T., Wu W., Lu L. (2023). Chlorogenic Acid Alleviates LPS-Induced Inflammation and Oxidative Stress by Modulating CD36/AMPK/PGC-1α in RAW264.7 Macrophages. Int. J. Mol. Sci..

[142] Zhang B., Tian M., Wu J., Qiu Y., Xu X., Tian C., Hou J., Wang L., Gao K., Yang X. (2024). Chlorogenic Acid Enhances the Intestinal Health of Weaned Piglets by Inhibiting the TLR4/NF-κB Pathway and Activating the Nrf2 Pathway. Int. J. Mol. Sci..

[143] Muthuswamy S., Rupasinghe H.P.V. (2007). Fruit phenolics as natural antimicrobial agents: Selective antimicrobial activity of catechin, chlorogenic acid and phloridzin. J. Food Agric. Environ..

[144] Bajko E., Kalinowska M., Borowski P., Siergiejczyk L., Lewandowski W. (2016). 5-O-Caffeoylquinic acid: A spectroscopic study and biological screening for antimicrobial activity. LWT.

[145] Kopjar M., Jakšić K., Piližota V. (2012). Influence of Sugars and Chlorogenic Acid Addition on Anthocyanin Content, Antioxidant Activity and Color of Blackberry Juice During Storage. J. Food Process. Preserv..

[146] Wang S., Liu Y., Wang X., Chen L., Huang W., Xiong T., Wang N., Guo J., Gao Z., Jin M. (2024). Modulating macrophage phenotype for accelerated wound healing with chlorogenic acid-loaded nanocomposite hydrogel. J. Control. Release.

[147] Mei Z., Sun J., Zhao P., Luo Y., Niu J., Huang D. (2026). Preliminary Anti-Melanoma Activity of a Chlorogenic Acid-Based PROTAC Targeting MDM4, a Candidate Protein Identified by Proteomics. Foods.

[148] Kimsa-Dudek M., Synowiec-Wojtarowicz A., Krawczyk A., Kosowska A., Kimsa-Furdzik M., Francuz T. (2022). The Apoptotic Effect of Caffeic or Chlorogenic Acid on the C32 Cells That Have Simultaneously Been Exposed to a Static Magnetic Field. Int. J. Mol. Sci..

[149] Neelakandan M., Manoharan S., Muralinaidu R., Thara J.M. (2023). Tumor preventive and antioxidant efficacy of chlorogenic acid–loaded chitosan nanoparticles in experimental skin carcinogenesis. Naunyn-Schmiedeberg’s Arch. Pharmacol..

[150] Tarasiuk A., Bulak K., Talar M., Fichna J. (2021). Chlorogenic acid reduces inflammation in murine model of acute pancreatitis. Pharmacol. Rep..

[151] Valgimigli L. (2023). Lipid Peroxidation and Antioxidant Protection. Biomolecules.

[152] Fatima N., Górecki A., Wiśniewska-Becker A. (2026). Curcumin–Lipid Interactions in PEGylated vs. Conventional Liposomes: A Combined Fluorescence and EPR Study. Membranes.

[153] Bonarska-Kujawa D., Cyboran-Mikołajczyk S., Kleszczyńska H. (2015). Molecular mechanism of action of chlorogenic acid on erythrocyte and lipid membranes. Mol. Membr. Biol..

[154] Cejas J.d.P., Disalvo E.A., Frias M.d.l.A. (2025). Effect of cholesterol on the antioxidant action of chlorogenic acid in lipid membranes. Arch. Biochem. Biophys..

[155] Lakey-Beitia J., Burillo A.M., La Penna G., Hegde M.L., Rao K.S. (2021). Polyphenols as Potential Metal Chelation Compounds Against Alzheimer’s Disease. J. Alzheimer’s Dis..

[156] Shahidi F., Samarasinghe A. (2025). How to assess antioxidant activity? Advances, limitations, and applications of in vitro, in vivo, and ex vivo approaches. Food Prod. Process. Nutr..

[157] Feng Y., Sun C., Yuan Y., Zhu Y., Wan J., Firempong C.K., Omari-Siaw E., Xu Y., Pu Z., Yu J. (2016). Enhanced oral bioavailability and in vivo antioxidant activity of chlorogenic acid via liposomal formulation. Int. J. Pharm..

[158] Bashkeran T., Harun A., Umakoshi H., Watanabe N., Mohd Nadzir M. (2024). Enhanced Curcumin Delivery and Stability through Natural Deep Eutectic Solvent-Based Niosomes. J. Mol. Liq..

[159] Xu H., Fu D., Zhang Y., Wang H., Su W., Song Y., Tan M. (2025). Curcumin-loaded proliposomes via glycerol-infused: Mechanism, stability and antioxidant activities. Food Chem..

[160] Baliyan S., Mukherjee R., Priyadarshini A., Vibhuti A., Gupta A., Pandey R.P., Chang C.-M. (2022). Determination of Antioxidants by DPPH Radical Scavenging Activity and Quantitative Phytochemical Analysis of Ficus religiosa. Molecules.

[161] Silvestrini A., Meucci E., Ricerca B.M., Mancini A. (2023). Total Antioxidant Capacity: Biochemical Aspects and Clinical Significance. Int. J. Mol. Sci..

[162] Nwachukwu I.D., Sarteshnizi R.A., Udenigwe C.C., Aluko R.E. (2021). A Concise Review of Current In Vitro Chemical and Cell-Based Antioxidant Assay Methods. Molecules.

[163] Sharma A., Kumar P., Islam A., Bhardwaj M., Kumar V., Prakash H. (2025). The neuroprotective role of chlorogenic acid and Fisetin in differentiated neuronal cell line-SHSY5Y against amyloid-β-induced neurotoxicity. Toxicol. Vitr..

[164] Ghodsi R., Sadr Tahouri S.K., Amjad F., Nasiri M.R., Toutounchi A., Salehi H., Moeinzadeh A., Sadeghi S., Soleimani N., Onagh B. (2025). Chitosan/alginate@niosome-curcumin: High-performance wound dressing with enhanced antibacterial activity. J. Drug Deliv. Sci. Technol..

[165] Nele V., Holme M.N., Kauscher U., Thomas M.R., Doutch J.J., Stevens M.M. (2019). Effect of Formulation Method, Lipid Composition, and PEGylation on Vesicle Lamellarity: A Small-Angle Neutron Scattering Study. Langmuir.

[166] Koehler J.K., Gedda L., Wurster L., Schnur J., Edwards K., Heerklotz H., Massing U. (2023). Tailoring the Lamellarity of Liposomes Prepared by Dual Centrifugation. Pharmaceutics.

[167] Andra V.V.S.N.L., Pammi S.V.N., Bhatraju L.V.K.P., Ruddaraju L.K. (2022). A Comprehensive Review on Novel Liposomal Methodologies, Commercial Formulations, Clinical Trials and Patents. BioNanoScience.

[168] Ahlström M.G., Bjerre R.D., Ahlström M.G., Skov L., Johansen J.D. (2024). Stratum Corneum Lipids in Non-Lesional Atopic and Healthy Skin following Moisturizer Application: A Randomized Clinical Experiment. Life.

[169] Brandner J.M. (2016). Importance of Tight Junctions in Relation to Skin Barrier Function.

[170] Chaves M.A., Ferreira L.S., Baldino L., Pinho S.C., Reverchon E. (2023). Current Applications of Liposomes for the Delivery of Vitamins: A Systematic Review. Nanomaterials.

[171] Zazuli Z., Hartati R., Rowa C.R., Asyarie S., Satrialdi (2025). The Potential Application of Nanocarriers in Delivering Topical Antioxidants. Pharmaceuticals.

[172] Mawazi S.M., Ge Y., Widodo R.T. (2025). Niosome Preparation Techniques and Structure—An Illustrated Review. Pharmaceutics.

[173] Raina N., Rani R., Thakur V.K., Gupta M. (2023). New Insights in Topical Drug Delivery for Skin Disorders: From a Nanotechnological Perspective. ACS Omega.

[174] Cui M., Wiraja C., Chew S.W.T., Xu C. (2021). Nanodelivery Systems for Topical Management of Skin Disorders. Mol. Pharm..

[175] Nsairat H., Khater D., Sayed U., Odeh F., Al Bawab A., Alshaer W. (2022). Liposomes: Structure, composition, types, and clinical applications. Heliyon.

[176] Almuqbil R.M., Aldhubiab B. (2025). Ethosome-Based Transdermal Drug Delivery: Its Structural Components, Preparation Techniques, and Therapeutic Applications Across Metabolic, Chronic, and Oncological Conditions. Pharmaceutics.

[177] Vallala R., Daravath B. (2026). Niosomes in drug delivery: A comprehensive review and therapeutic perspectives. Next Nanotechnol..

[178] Raj A., Dua K., Nair R.S., Sarath Chandran C., Alex A.T. (2023). Transethosome: An ultra-deformable ethanolic vesicle for enhanced transdermal drug delivery. Chem. Phys. Lipids.

[179] Lila A.S.A., Mostafa M., Katamesh A.A., Ibrahim M., Hassoun S.M., El Sayed M.M., Subaiea G.M., Abdallah M.H., Qelliny M.R. (2025). Vesicular drug delivery systems: A breakthrough in wound healing therapies. Colloids Surf. B Biointerfaces.

[180] Blume G.D., Sacher M., Teichmüller D., Schäfer U.F. (2003). The role of liposomes and their future perspective. SOFW J..

[181] Verma D.D., Verma S., Blume G., Fahr A. (2003). Particle size of liposomes influences dermal delivery of substances into skin. Int. J. Pharm..

[182] Devaki J., Pavuluri S., Suma N. (2023). Ethosomes: A Vesicular Carrier as a Novel Tool for Transdermal Drug Delivery System. J. Drug Deliv. Ther..

[183] Abasi M., Ghadi Z.S., Pilehvar Y., Ebrahimnejad P. (2025). Curcumin loaded hyaluronan modified niosomes: Preparation, characterization and anti-cancer activity on triple-negative breast cancer cells. J. Drug Deliv. Sci. Technol..

[184] Mohaddish, Jilani M., Rehman U., Ramaiah R., Hani U., Gupta G., Goh K.W., Kesharwani P. (2025). Recent advances in the clinical application of transferosomes for skin cancer management. Colloids Surf. B Biointerfaces.

[185] Jacob S., Varkey N.R., Nair A.B. (2026). Ultradeformable Vesicles for Wound Healing: Ethosomes, Transferosomes, and Transethosomes in Topical Drug Delivery. Pharmaceutics.

[186] Alshawwa S.Z., Kassem A.A., Farid R.M., Mostafa S.K., Labib G.S. (2022). Nanocarrier Drug Delivery Systems: Characterization, Limitations, Future Perspectives and Implementation of Artificial Intelligence. Pharmaceutics.

[187] Lombardo D., Kiselev M.A. (2022). Methods of Liposomes Preparation: Formation and Control Factors of Versatile Nanocarriers for Biomedical and Nanomedicine Application. Pharmaceutics.

[188] Zhan B., Wang J., Li H., Xiao K., Fang X., Shi Y., Jia Y. (2024). Ethosomes: A Promising Drug Delivery Platform for Transdermal Application. Chemistry.

[189] Ascenso A., Raposo S., Batista C., Cardoso P., Mendes T., Praça F.G., Bentley M.V., Simões S. (2015). Development, characterization, and skin delivery studies of related ultradeformable vesicles: Transfersomes, ethosomes, and transethosomes. Int. J. Nanomed..

[190] Giordani S., Marassi V., Zattoni A., Roda B., Reschiglian P. (2023). Liposomes characterization for market approval as pharmaceutical products: Analytical methods, guidelines and standardized protocols. J. Pharm. Biomed. Anal..

[191] Tomnikova A., Orgonikova A., Krizek T. (2022). Liposomes: Preparation and characterization with a special focus on the application of capillary electrophoresis. Monatsh Chem..

[192] Chen C., Zhu S., Huang T., Wang S., Yan X. (2013). Analytical techniques for single-liposome characterization. Anal. Methods.

[193] Chauhan N., Vasava P., Khan S.L., Siddiqui F.A., Islam F., Chopra H., Emran T.B. (2022). Ethosomes: A novel drug carrier. Ann. Med. Surg..

[194] Seenivasan R., Halagali P., Nayak D., Tippavajhala V.K. (2025). Transethosomes: A Comprehensive Review of Ultra-Deformable Vesicular Systems for Enhanced Transdermal Drug Delivery. AAPS PharmSciTech.

[195] Fernández-García R., Lalatsa A., Statts L., Bolás-Fernández F., Ballesteros M.P., Serrano D.R. (2020). Transferosomes as nanocarriers for drugs across the skin: Quality by design from lab to industrial scale. Int. J. Pharm..

[196] Bindhani S., Nayak A.K. (2025). Nanovesicles as Potential Carriers for Delivery of Antiviral Drugs: A Comprehensive Review. Curr. Drug Deliv..

[197] Wang C., Wang Y., Lan X., Zhu L., Gu J., Hou W., Zhai B., Li D., Tian H., Xu W. (2025). Multidimensional Delivery Strategies for Liposomal Transdermal Drug Delivery Systems. ACS Mater. Lett..

[198] Rahma S., Amalia E., Kusuma S.A.F. (2026). Surfactant-Engineered Niosomal Antibiotic Systems for Biofilm-Associated Infections: Design Principles and Translational Perspectives. Int. J. Nanomed..

[199] Shabreen O.R., Sangeetha S. (2020). Ethosomes: A Novel Drug Delivery System And Their Therapeutic Applications—A Review. Res. J. Pharm. Technol..

[200] Alquraisy A., Ramadhani K., Mohammed A.F.A., Wilar G., Osman W., Elamin K.M., Wathoni N. (2026). Nanostructured Lipid Carrier-Gels for Wound Healing: A Narrative Review of Formulation Strategies, Mechanisms, and Translational Potential. Nanotechnol. Sci. Appl..

[201] Katsarov P., Simeonov P., Apostolova E., Gvozdeva Y., Boyuklieva R., Lukova P., Bivolarski I., Koleva M., Delattre C., Kokova V. (2026). Design, Characterization, and Wound-Healing Evaluation of Sodium Humate Transferosome-Loaded Alginate/HPMC Dermal Patches. Pharmaceutics.

[202] Bautista-Solano A.A., Dávila-Ortiz G., Perea-Flores M.D.J., Martínez-Ayala A.L. (2025). A Comprehensive Review of Niosomes: Composition, Structure, Formation, Characterization, and Applications in Bioactive Molecule Delivery Systems. Molecules.

[203] Poornima G., Deepa M., Devadharshini M., Gopan G., Mani M., Kannan S. (2024). In-situ synthesis and evaluation of anti-bacterial efficacy and angiogenesis of curcumin encapsulated lipogel dermal patch for wound healing applications. Biomater. Adv..

[204] Jain H., Geetanjali D., Dalvi H., Bhat A., Godugu C., Srivastava S. (2022). Liposome mediated topical delivery of Ibrutinib and Curcumin as a synergistic approach to combat imiquimod induced psoriasis. J. Drug Deliv. Sci. Technol..

[205] Ternullo S., Gagnat E., Julin K., Johannessen M., Basnet P., Vanić Ž., Škalko-Basnet N. (2019). Liposomes augment biological benefits of curcumin for multitargeted skin therapy. Eur. J. Pharm. Biopharm..

[206] Lu B., Zhang J., Zhang J. (2023). Enhancing Transdermal Delivery of Curcumin-Based Ionic Liquid Liposomes for Application in Psoriasis. ACS Appl. Bio Mater..

[207] Balasubramanyam P., Sudheer P., Sreeharsha N., Nair A.B., Ramachandra D.P. (2025). Transfersomes Mediated Transdermal Delivery of Curcumin: In Vitro and In Vivo Evaluation. Pharm. Sci..

[208] Luthfi M.A., Husna I.T., Dahlizar S., Meliana Y., Septama A.W. (2025). Innovative Niosome Gel Loaded Curcuma Xanthorrhiza Extract: Development Formulation, Characterization, and Evaluation of Their Antibacterial Activity. J. Pharm. Innov..

[209] Sadeghi Ghadi Z., Dinarvand R., Asemi N., Talebpour Amiri F., Ebrahimnejad P. (2019). Preparation, characterization and in vivo evaluation of novel hyaluronan containing niosomes tailored by Box-Behnken design to co-encapsulate curcumin and quercetin. Eur. J. Pharm. Sci..

[210] Akbari J., Saeedi M., Enayatifard R., Morteza-Semnani K., Hassan Hashemi S.M., Babaei A., Rahimnia S.M., Rostamkalaei S.S., Nokhodchi A. (2020). Curcumin Niosomes (curcusomes) as an alternative to conventional vehicles: A potential for efficient dermal delivery. J. Drug Deliv. Sci. Technol..

[211] Li Y., Xu F., Li X., Chen S.-Y., Huang L.-Y., Bian Y.-Y., Wang J., Shu Y.-T., Yan G.-J., Dong J. (2021). Development of curcumin-loaded composite phospholipid ethosomes for enhanced skin permeability and vesicle stability. Int. J. Pharm..

[212] Guo T., Lu J., Fan Y., Zhang Y., Yin S., Sha X., Feng N. (2021). TPGS assists the percutaneous administration of curcumin and glycyrrhetinic acid coloaded functionalized ethosomes for the synergistic treatment of psoriasis. Int. J. Pharm..

[213] Rawat V., Dewangan S., Sarwa K.K., Dhara M., Prusty S.K. (2025). Curcumin-loaded gelatin ethosomal gel: A novel approach for anti-inflammatory efficacy. Pharm. Sci. Adv..

[214] Rezigue M., Aljabali A.A.A., Alshaer W. (2025). Transethosome-mediated curcumin delivery promotes superior wound healing with reduced toxicity. Pharmacia.

[215] Trivedi H.R., Puranik P.K. (2025). Comparative Study of Chlorogenic Nanotransfersomes and Nanoprotransfersomes for Enhanced Transdermal Therapeutic Application. BioNanoScience.

[216] Li X., Zhu S., Yin P., Zhang S., Xu J., Zhang Q., Shi S., Zhang T. (2021). Combination immunotherapy of chlorogenic acid liposomes modified with sialic acid and PD-1 blockers effectively enhances the anti-tumor immune response and therapeutic effects. Drug Deliv..

[217] Atre P., Rizvi S.A.A. (2024). A brief overview of quality by design approach for developing pharmaceutical liposomes as nano-sized parenteral drug delivery systems. RSC Pharm..

[218] Chelliah R., Rubab M., Vijayalakshmi S., Karuvelan M., Barathikannan K., Oh D.-H. (2025). Liposomes for drug delivery: Classification, therapeutic applications, and limitations. Next Nanotechnol..

[219] Zhang X., Liu W., Chu H., Zong W. (2026). Liposomes: A breakthrough in advanced drug delivery systems. Adv. Colloid. Interface Sci..

[220] Balash M., Boucetta H., He W. (2025). Factors affecting drug delivery system translation: A focus on advanced technologies, biological barriers, and regulatory challenges. J. Control. Release.

